# Regiospecific, Diastereo- and Enantioselective Allylboration with Methylenecyclobutylboranes: Experimental and Computational Study

**DOI:** 10.3390/ijms27188277

**Published:** 2026-09-17

**Authors:** Oleg A. Mikhaylov, Angelina Kolchina, Ivan I. Murygin, Albert G. Nigmatov, Ivan S. Arteev, Ilya D. Gridnev

**Affiliations:** 1 N. D. Zelinsky Institute of Organic Chemistry, Russian Academy of Sciences, Leninsky Prosp. 47, Moscow 119991, Russia; mikhaylov.o@ioc.ac.ru (O.A.M.); kol4inaaa@yandex.ru (A.K.); ivanmurygin642@gmail.com (I.I.M.); nig@ioc.ac.ru (A.G.N.); i.arteev@ioc.ac.ru (I.S.A.); 2 Faculty of Science, Mathematics and Natural Sciences, Peoples’ Friendship University of Russia (RUDN University), Miklukho-Maklay Str. 6, Moscow 117198, Russia

**Keywords:** allylboration, medicinal chemistry, boratropic rearrangement, asymmetric synthesis, DFT calculations, Soai reaction

## Abstract

New methylenecyclobutane-derived allylboranes, diisopropyl(2-methylenecyclobutyl)borane and chiral (+)-diisopinocampheyl(2-methylenecyclobutyl)borane undergo rapid equilibration via facile [1,3]-B sigmatropic shifts that interconvert the endocyclic and exocyclic isomers. Both endocyclic isomers react with the Soai aldehyde, 2-(3,3-dimethylbut-1-yn-1-yl)pyrimidine-5-carbaldehyde, affording a single diastereomer of the corresponding homoallylic alcohol. With the achiral reagent the reaction is regio- and diastereoselective, yielding exclusively one diastereomer of the racemic alcohol in 83% yield. With the chiral reagent, the reaction is enantioselective, providing optically active (*RR*)-alcohol in 87% yield and 89% ee, demonstrating the synthetic potential of the new chiral organoboron reagent. The regio- and diastereospecific allylboration with the achiral reagent and the regio-, diastereo- and enantioselective allylboration with the novel chiral reagent, as well as their reactions with pyrimidinic aldehyde yielding new alcohol, were analyzed computationally, revealing the origins of high selectivity in dynamic multicomponent reaction mixtures. The exocyclic isomers are unreactive due to the universally high barriers of allylboration reactions with their participation. Relative stabilities of all intermediates and transition states are regulated by the effectiveness of weak non-covalent interactions in the corresponding species. X-Ray crystallographic analysis of the racemate, consisting of (*RR*) and (*SS*) enantiomers, confirmed its constitution and relative configuration, whereas the absolute configuration of the chiral compound was assigned on the basis of the DFT calculation results. The chiral alcohol was tested as a catalyst in the Soai reaction, which afforded a racemic product.

## 1. Introduction

Addition of allylboranes to carbonyl compounds is one of the most reliable methods for the stereocontrolled construction of carbon–carbon bonds [1,2,3].

**Scheme 1:**
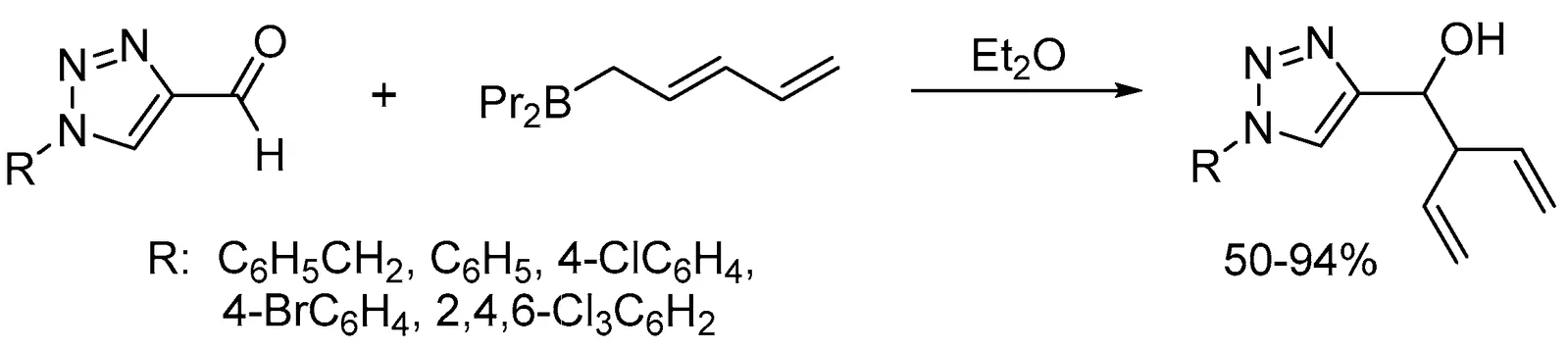
Reactions of azole carbaldehydes with 2,4-pentadienyl(dipropyl)borane [4].

Recently, we reported the allylboration of azole carbaldehydes with 2,4-pentadienyl(dipropyl)borane that exclusively furnished the products formed with allylic rearrangement, the corresponding 2-butenyl-substituted homoallylic alcohols, confirming that γ-attack operates exclusively under the non-catalytic conditions (Scheme 1) [4]. The same study assigned the (*R*) configuration to the products obtained with (+)-*B*-allyldiisopinocampheylborane. Computations demonstrated that the enantioselection is driven by an extended network of attractive CH···HC contacts rather than the previously assumed destabilizing steric interactions. This interpretation is consistent with the broadly documented role of London dispersion as an attractive, structure-determining force in sterically congested molecules [5,6].

Methylenecyclobutane (MCB) scaffold represents an attractive target for this methodology. Cyclobutane derivatives are found in numerous biologically active natural products [7,8,9] and, owing to their conformational rigidity and pronounced three-dimensional character, are increasingly exploited as saturated scaffolds and bioisosteres in medicinal chemistry [10,11]. The inherent strain of the four-membered ring, in turn, makes MCB derivatives valuable substrates for ring-expansion and ring-cleavage transformations [12], while unsaturated four-membered rings have evolved from synthetic curiosities into isolable and broadly useful building blocks [13]. Allylboration has already been enlisted in this area: merging boron homologation with boron allylation delivers methylenecyclobutanes as essentially single diastereomers [14], and cyclobutenyl metal species provide one-pot access to both cyclobutenes and alkylidenecyclobutanes [15]. An asymmetric single-pot variant of the reaction furnished alkylidenecyclobutanes bearing a quaternary stereocenter with excellent stereoisomeric ratios [16]. However, these methods rely on multistep procedures and the intrinsic mechanism of stereoselection in these reactions has remained unexplored so far. Here we report the synthesis of new allylboranes, diisopropyl (2-methylenecyclobutyl)borane **1** and chiral (+)-diisopinocampheyl(2-methylenecyclobutyl)borane **2**, capable of stereoselective introduction of the 2-methylenecyclobutyl group in various frameworks via the reaction with corresponding carbonyl compounds.

As a continuation of our recent search for possible cases of autoamplifying catalysis and spontaneous chirality generation in the reactions of organometallic reagents with Soai-like substrates [17,18], we decided to explore the reactions of the boranes derived from methylenecyclobutanes with one of the Soai aldehydes, viz. 2-(3,3-dimethylbut-1-yn-1-yl)pyrimidine-5-carbaldehyde **3**. Since secondary alcohols with this scaffold are potential asymmetric autocatalysts, their functionalized derivatives attract significant interest extending beyond the immediate scope of this methodology.

## 2. Results and Discussion

### 2.1. Synthesis and Properties of New Methylenecyclobutylboranes

**Scheme 2:**
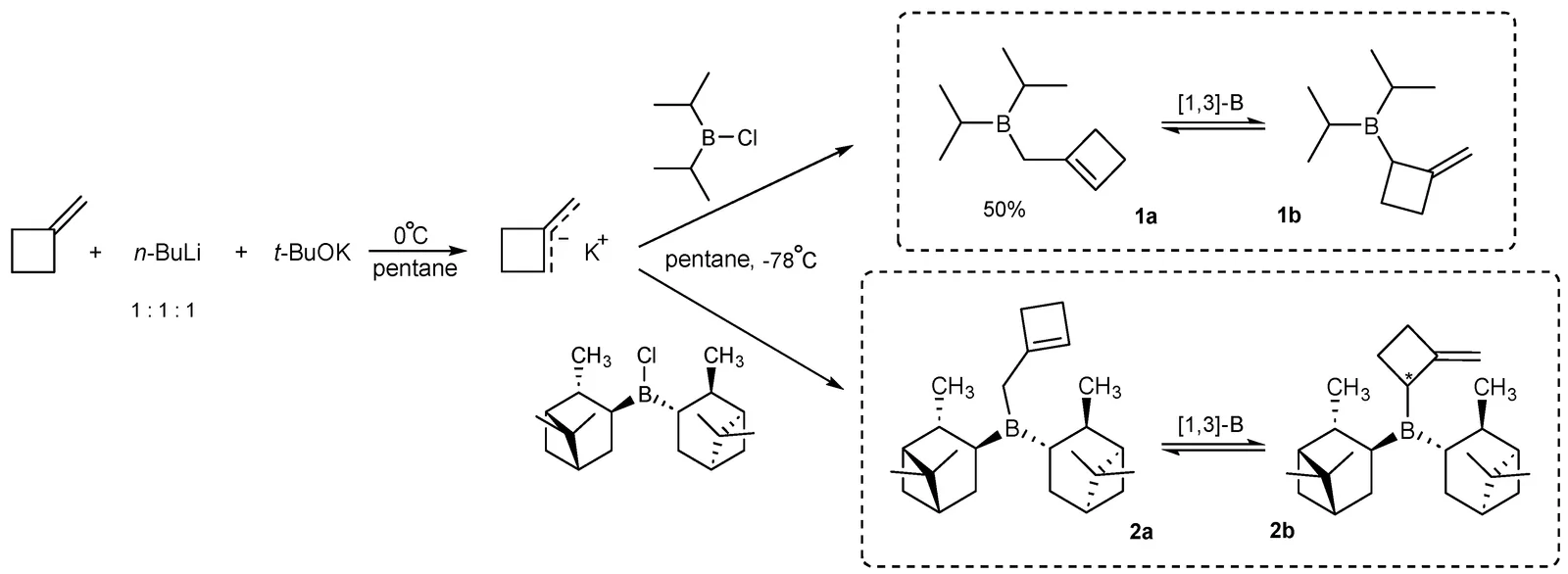
Synthesis of methylenecyclobutylboranes **1** and **2**.

Equilibrium mixtures of **1a**,**b** and **2a**,**b** were prepared by treatment of the potassium–lithium reagent derived from methylenecyclobutane with *i*-Pr_2_BCl or (+)-Ipc_2_BCl at −78 °C following the reported procedure for aliphatic and bicyclic boranes (Scheme 2) [19].

The NMR spectra of the mixture of **1a** and **1b** at room temperature demonstrate very broad signals due to their rapid equilibration via [1,3]-B shifts. At −85 °C, resolved ^1^H NMR spectra were observed, allowing assignment of the signals. Rapid equilibration of **1a** and **1b** implies that either of them is kinetically available in a broad temperature range.

**Figure 1:**
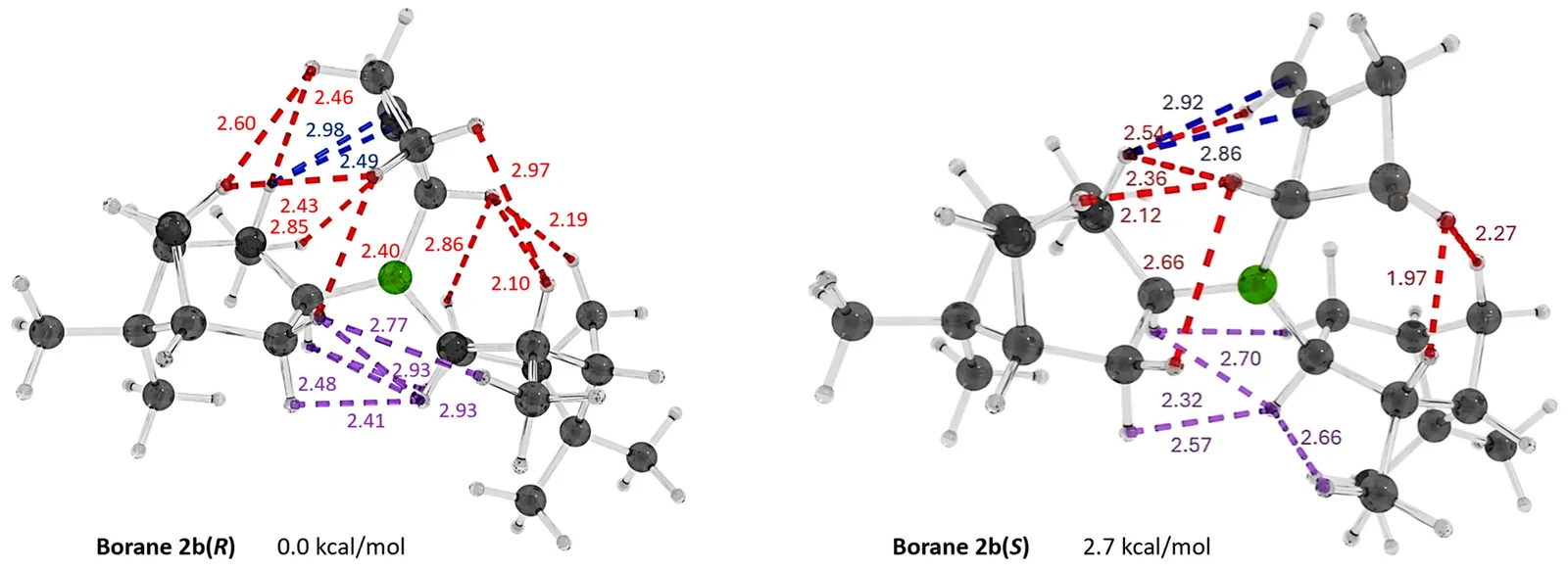
Optimized structures, relative energies and close contacts of the boranes **2b**(*R*) and **2b**(*S*) (TPSSh/def2-SVP/SMD(diethyl ether)). Atoms: C, black; H, gray; B, green. Non-covalent contacts: CH^…^HC between methylenecyclobutane unit and isopinocampheyl groups, red; CH^…^π contacts between CH_2_ group of methylenecyclobutane and isopinocampheyl groups, blue; CH^…^HC contacts between two isopinocampheyl groups, violet.

The chiral center in the exocyclic isomer **2b** renders the existence of a diastereomeric pair **2b**(*R*) and **2b**(*S*) (Figure 1). This fact significantly complicates the analysis of the ^1^H and ^13^C spectra that remain significantly broadened in the wide temperature interval. This prompted us to perform more detailed computational studies (vide infra).

Noteworthy is the significant difference in energy between **2b**(*R*) and **2b**(*S*), that appears due to the relatively small size of the molecules and the extended network of close CH^…^HC and CH^…^π contacts in both diastereomers. The diastereomer **2b**(*R*) has three more CH^…^HC contacts between the methylenecyclobutane unit and isopinocampheyl groups and one additional CH^…^HC contact between isopinocampheyl groups that provides a reasonable estimation of ~0.7 kcal/mol for each non-covalent interaction.

### 2.2. Reaction of Boranes ***1a***,***b*** with Pyrimidine-5-Carbaldehyde ***3***

**Scheme 3:**
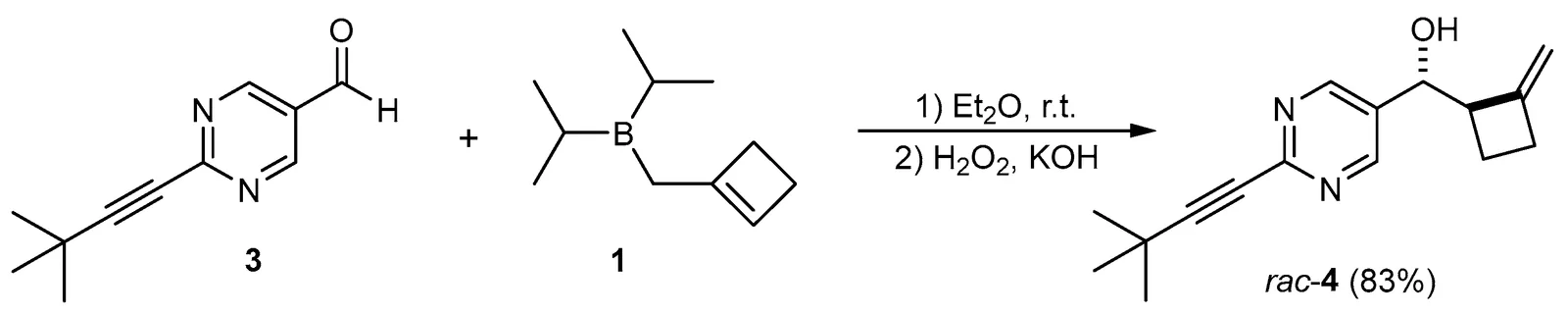
Reaction of pyrimidine-5-carbaldehyde **3** with borane **1**.

The reaction of **1** with aldehyde **3** proceeded to completion within minutes at ambient temperature under conditions similarly to the allylboration of azole carbaldehydes [4,17], affording the homoallylic alcohol **4** in 83% yield after chromatography (Scheme 3).

The constitution of the product definitively identifies the reacting allylic system. Consequently, the formation of a product featuring an exocyclic double bond establishes that C–C bond formation occurs at the ring carbon of the endocyclic isomer, accompanied by an exocyclic shift in the double bond via the allylic rearrangement.

Analysis of the product by 1D and 2D NMR spectroscopy revealed three key spectral features that unambiguously establish its constitution. A two-proton multiplet at *δ* 4.86–4.93 is characteristic of an exocyclic methylene group; the carbinol proton appears at *δ* 4.73 as a doublet (*J* = 7.8 Hz), indicating coupling to a single methine proton rather than a methylene group; and the ring methine resonates at *δ* 3.24–3.30. This resonance pattern exclusively corresponds to the 2-methylenecyclobutyl carbinol **4** and rules out the alternative cyclobutenylmethyl carbinol, wherein the carbinol proton would be coupled to a methylene group, and the ring would bear a vinylic proton.

**Figure 2:**
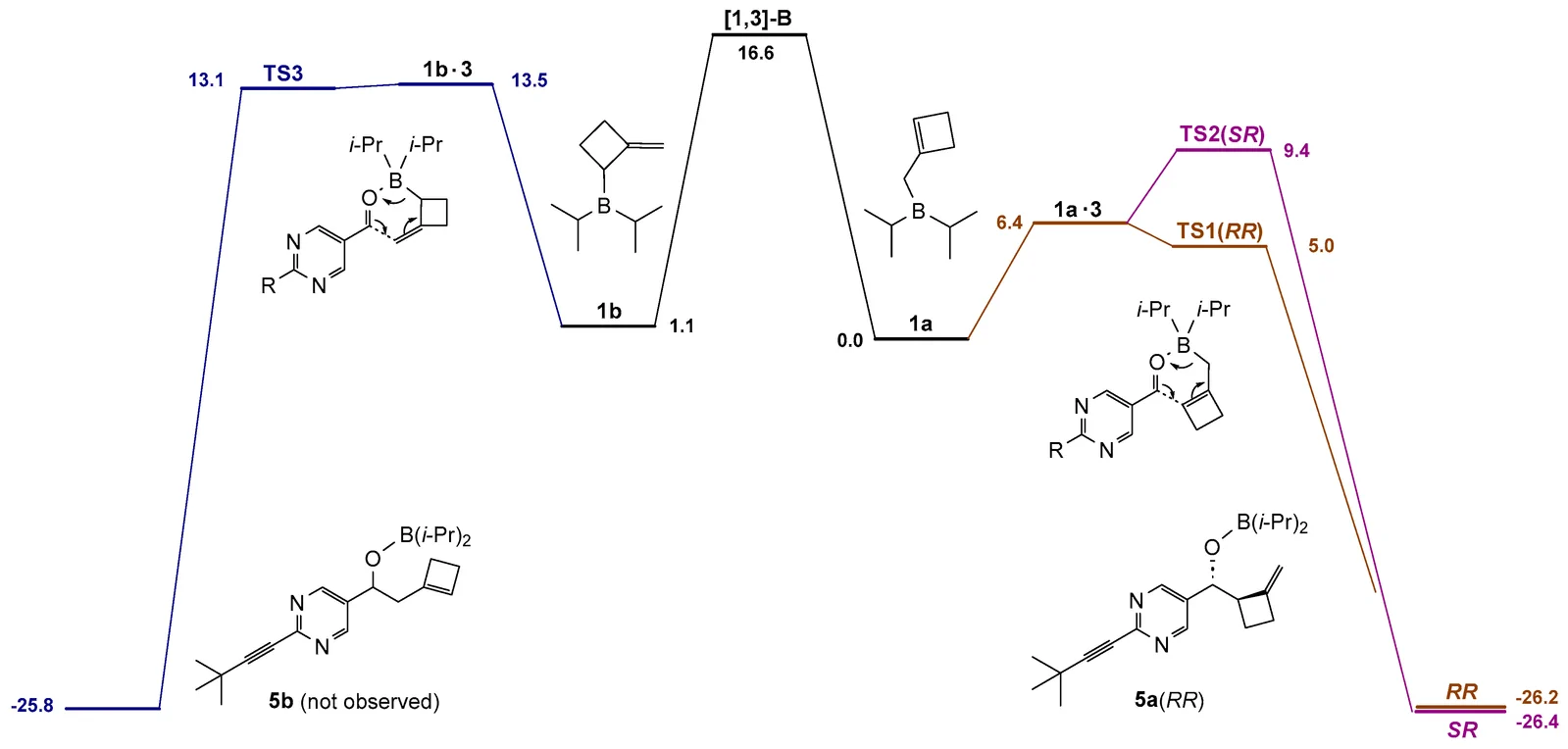
Computed profile of relative Gibbs free energy changes for the reaction of equilibrating boranes **1a**,**b** with heterocyclic aldehyde **3.** The computations were carried out at the TPSSh/def2-SVP level of theory, taking into account the solvent effects by applying SMD continuum solvation model for diethyl ether.

To elucidate the origin of regio- and diastereoselectivity in allylboration of **3** with equilibrium mixture of **1a** and **1b**, DFT calculations of different possible reaction pathways were carried out (Figure 2).

After endergonic formation of adduct **1a** ∙ **3**, either of the two prochiral planes of the aldehyde, **TS1**(*RR*) or **TS2**(*SR*), can be used to form a six-membered state that leads to the *RR* and *SR* diastereomers of boronate **5**, respectively. Computations indicate that the formation of the *RR* isomer is barrierless and the **TS1**(*RR*) is 4.4 kcal/mol more stable than the **TS2**(*SR*) that corresponds to the exclusive formation of **5**(*RR*) observed experimentally.

On the other hand, the transition state of the reaction of **1b** with **3** (**TS3**) is significantly higher in energy than either **TS1**(*RR*) or **TS2**(*SR*), in accord with the experimental observations.

**Figure 3:**
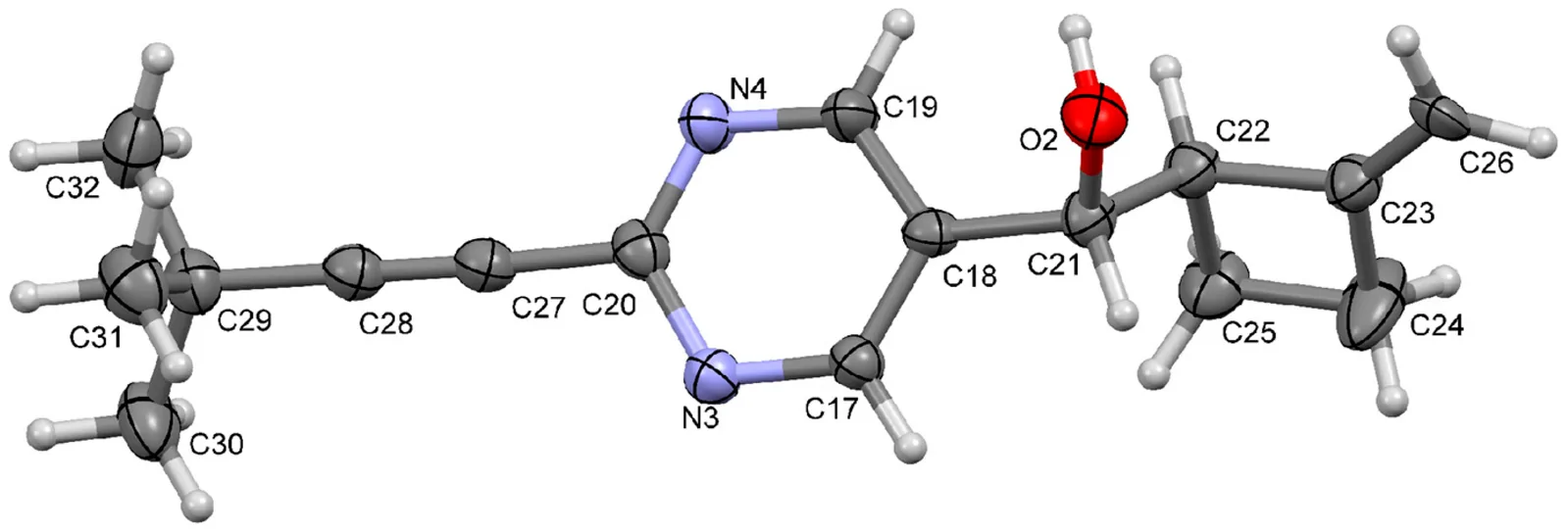
The solid-state structure of racemic alcohol **4**.

Single crystals of racemic **4** suitable for X-ray diffraction were grown. The solid-state structure (Figure 3) unambiguously verifies the connectivity of the product, confirming the intact cyclobutane ring alongside the exocyclic methylene group, and establishes the relative configuration of the two contiguous stereocenters as *anti*, the racemate consisting of the (*R*,*R*) and (*S*,*S*) enantiomers. The asymmetric unit contains one (*R*,*R*) and one (*S*,*S*) molecule. A comparably high level of diastereocontrol is characteristic of allylboration-based routes to the methylenecyclobutane motif, where boron-homologation/allylboration sequences similarly afford the strained carbocycle as a single diastereomer [14,15,16].

### 2.3. Enantioselective Variant

**Scheme 4:**
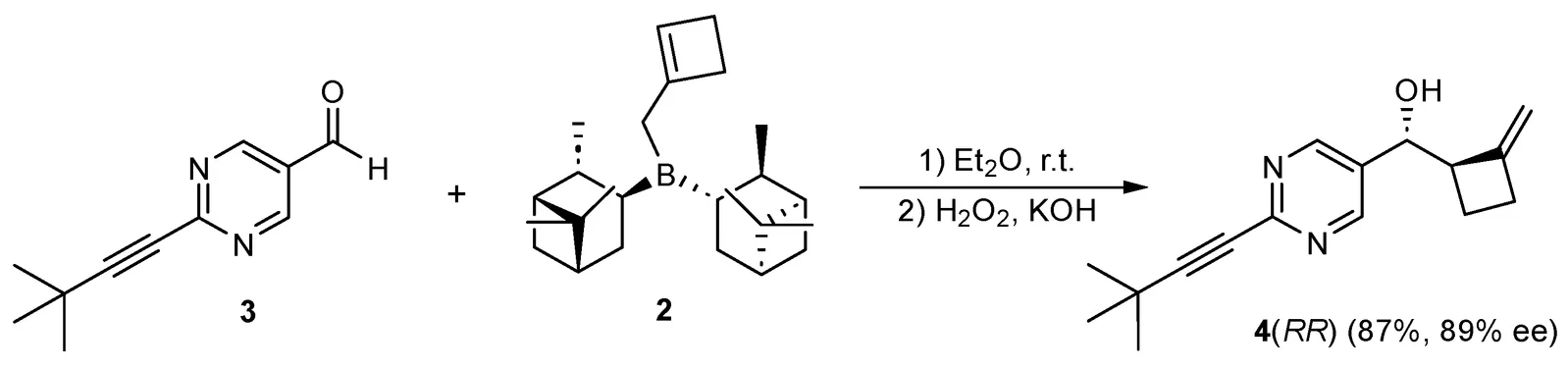
Enantioselective methylenecyclobutylboration of aldehyde **3**.

The reaction of the equilibrium mixture of **2a**,**b** with aldehyde **3**, conducted under the conditions established for the achiral reagent, afforded the chiral alcohol **4** in 87% yield with 89% ee; the alternative regio-isomer was not detected and the product was isolated as a single diastereomer (Scheme 4).

The introduction of bulky isopinocampheyl ligands thus renders the addition enantioselective while preserving its strict regioselectivity and diastereocontrol—a combination of relative and absolute stereocontrol that has similarly been realized in one-pot allylboration approaches to enantioenriched alkylidenecyclobutanes [16]. The absolute configuration of **4** at the newly formed carbinol stereocenter was assigned as (*R*) based on the computed sense of induction (Section 3.1) and by analogy with the products of the allylboration of azole carbaldehydes with (+)-*B*-allyldiisopinocampheylborane, for which the (*R*) configuration was established via X-ray analysis of an enantiopure heavy-atom derivative [4].

### 2.4. Computational Study of the Enantioselective Allylboration

**Figure 4:**
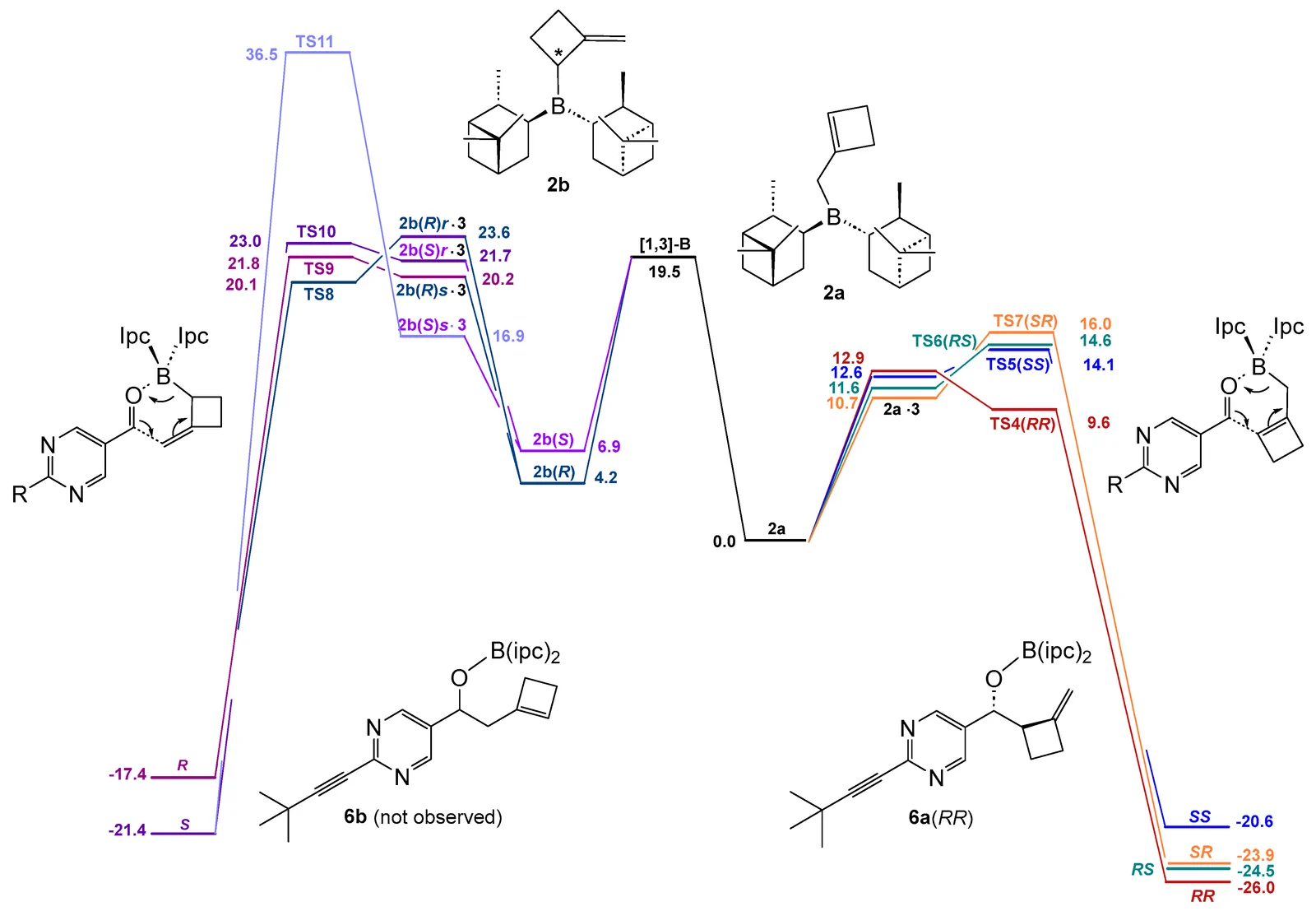
Computed profile of relative Gibbs free energy changes for the reaction of equilibrating boranes **2a**,**b** with heterocyclic aldehyde **3**. The computations were carried out at the TPSSh/def2-SVP level of theory. The solvent effects were modeled by applying SMD continuum solvation model for diethyl ether. Asterics denotes the asymmetric carbon atom.

Although the boranes **2b**(*R*) and **2b**(*S*) are less stable than **2a** by 4.2 and 6.9 kcal/mol respectively, they could be kinetically available via the allylboron rearrangement. However, the computed barriers for the [1,3]-B shift and representative reactions of **2b**(*R*) and **2b**(*S*) with **3** are significantly higher in free energy than any of the four computed pathways of the allylboration of **3** with **2a** (Figure 4). This result is in good agreement with the experimental data, since the structure of the single reaction product **4** implies that it has been formed via the reaction of **2a** with **3** accompanied with allylic rearrangement.

**Figure 5:**
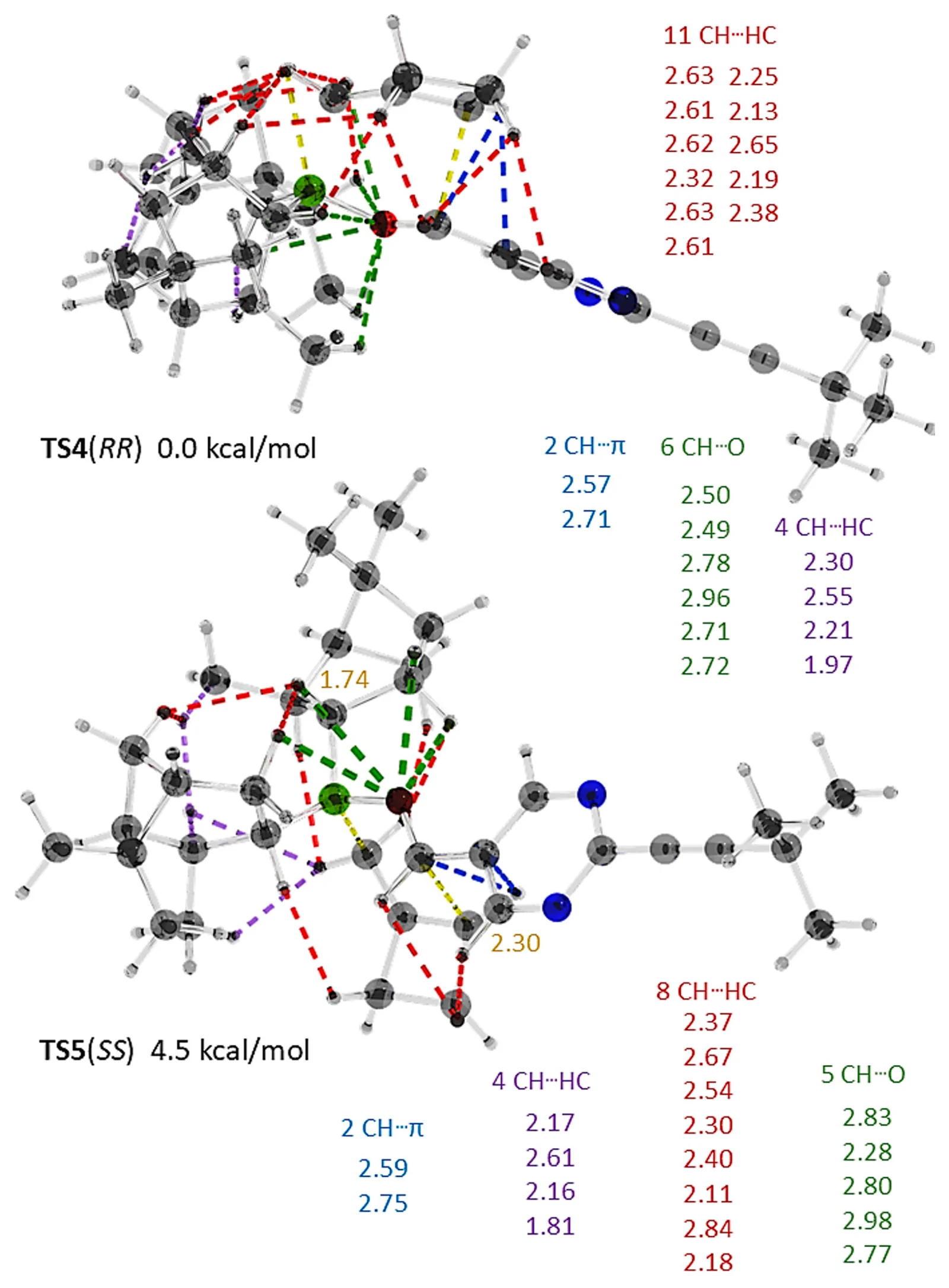
Optimized structures, relative energies and close contacts of the transition states **TS4**(*RR*) and **TS5**(*SS*) of the allylboration of the aldehyde **3** with the borane **2a** (TPSSh/def2-SVP/SMD(diethyl ether)). Atoms: C, black; H, gray; B, green; N, blue; O, red. Non-covalent contacts: CH^…^HC between methylenecyclobutane unit and isopinocampheyl groups, red; CH^…^π, blue; CH^…^HC contacts between two isopinocampheyl groups, violet, CH^…^O, green.

The allylboration of **3** with **2a** requires initial endergonic formation of the complexes **2a** ∙ **3** that have approximately equal stabilities for all four competing routes. Nevertheless, only for the pathway leading to **6**(*RR*) does the allylboration proceed via **TS4**(*RR*) that is lower in Gibbs free energy than the corresponding complex **2a** ∙ **3**, whereas three other pathways must overcome additional barriers. As a result, the **TS5**(*SS*) standing next in the order of stability after **TS4**(*RR*) is 4.5 kcal/mol less stable. Optimized geometries of the two most stable transition states are shown in Figure 5.

Looking for the structural reasons of the computed difference in the stabilities of the transition states, one can see from Figure 5 that in **TS4**(*RR*), the aldehyde and the allylborane adopt a well-ordered chair-like arrangement, which places the reacting centers in close proximity appropriate for C–C bond formation while the bulky isopinocampheyl substituents avoid unfavorable steric clashes. Several weak non-covalent interactions between the reacting species including C–H···O contacts between the aldehyde oxygen and the isopinocampheyl C–H bonds, as well as C–H···π interactions involving the pyrimidine ring, further stabilize this transition state.

The transition state **TS5**(*SS*) has one C–H···O and three C–H···HC contacts fewer than **TS4**(*RR*), which accounts for the computed free energy difference between them and is in accord with the high enantioselectivity observed experimentally.

Overall, the computational results provide a clear mechanistic rationale for the observed stereoselectivity. The lowest activation barrier for the *RR* pathway fully accounts for the exclusive formation of this diastereomer. The computed effective barrier height (12.9 kcal/mol) is consistent with the reaction proceeding smoothly at room temperature, as observed experimentally.

### 2.5. Probing the Chiral Alcohol ***4*** as a Catalyst in the Soai Reaction

**Scheme 5:**
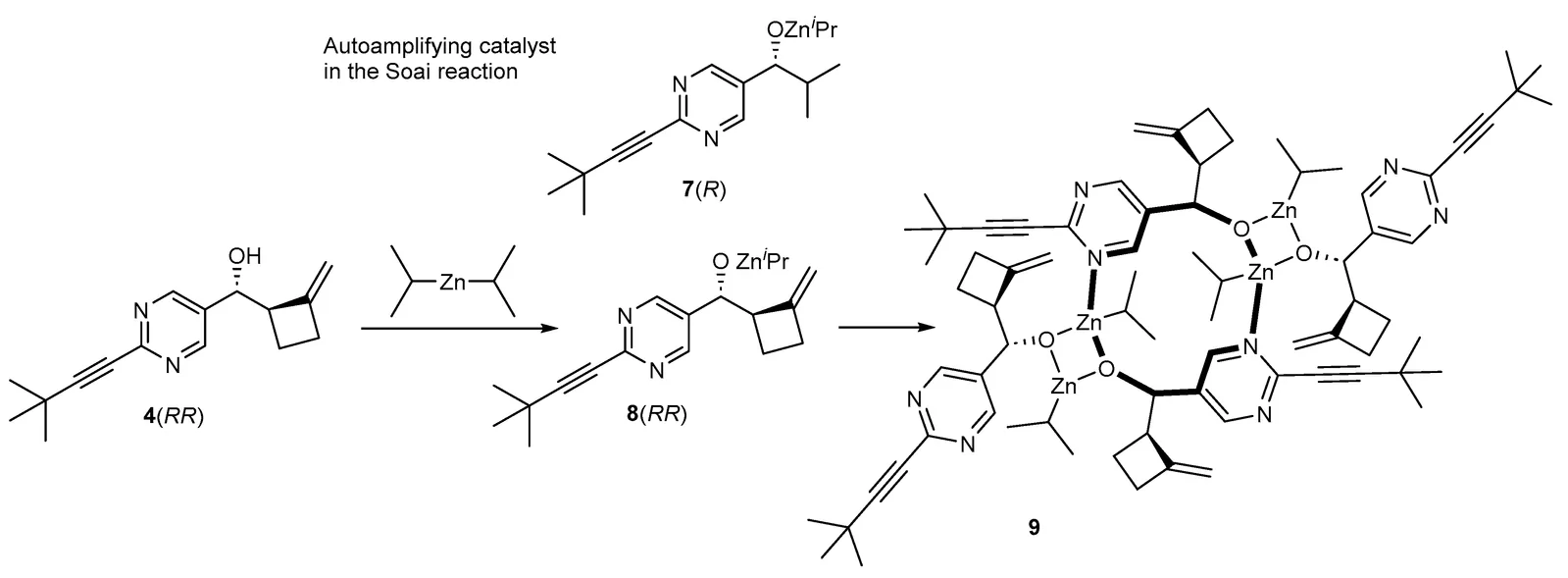
Formation of the Zn alcoholate **8** and the tetramer **9** with four methylenecyclobutane units.

The structure of the alcohol **4** is very close to the product of the Soai reaction (**7**) that can increase its own chirality via Asymmetric Autocatalytic Amplification (AAA) [17,20,21,22]. The only difference between **4** and **7** is the structure of the substituent connected to the asymmetric center. Accordingly, in the possible catalyst **9**, four alcoholates constituting the tetrameric structure would be methylenecyclobutyls, whereas four isopropyl groups would remain connected to the Zn atoms (Scheme 5). We were therefore interested if the chiral alcohol **4**(*RR*) could work as an asymmetric catalyst in the Soai reaction [23,24,25].

**Scheme 6:**
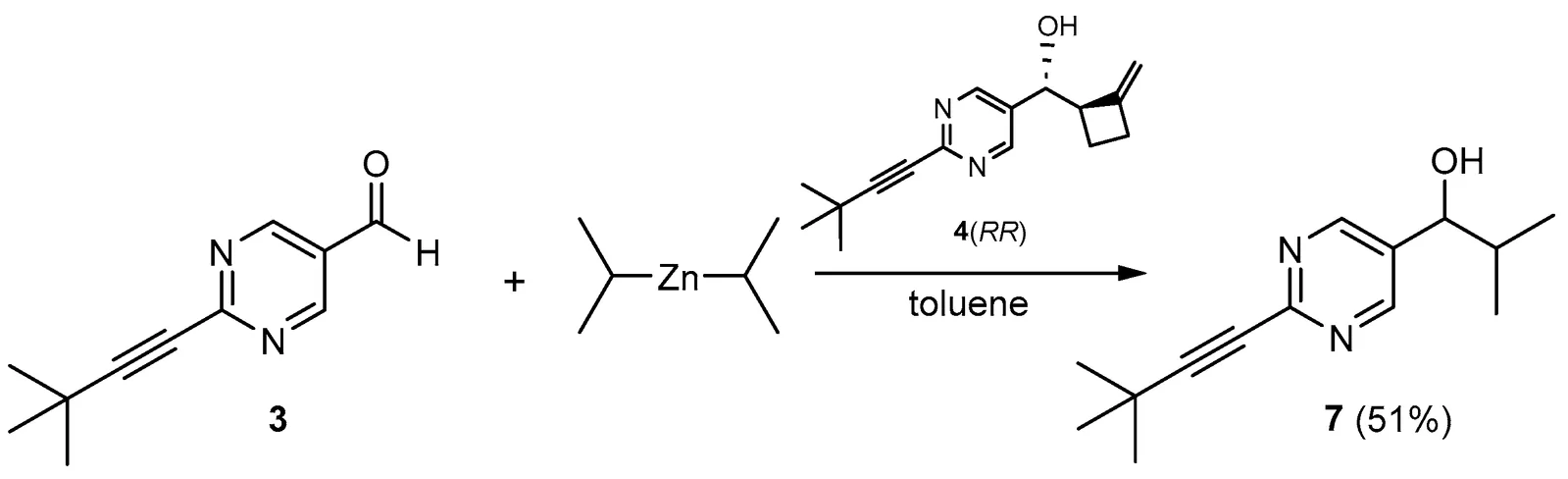
Reaction of **3** with diisopropylzinc in the presence of **4**(*RR*) as a chiral catalyst.

After addition of 11-fold excess of Zn(*i*-Pr)_2_ to a solution of **4** in toluene, solution of 10 equivalents of **3** in toluene was added dropwise at room temperature and the reaction mixture was stirred for 2 h (Scheme 6). Mixture of **4** and **7** obtained after standard work-up and purification was subjected to HPLC analysis, demonstrating that **4** has kept its chirality, whereas **7** was obtained as a racemate.

At first sight this result might have looked disappointing; however, it provides important evidence for the strictness of structural requirements providing the possibility of AAA in the Soai reaction.

**Figure 6:**
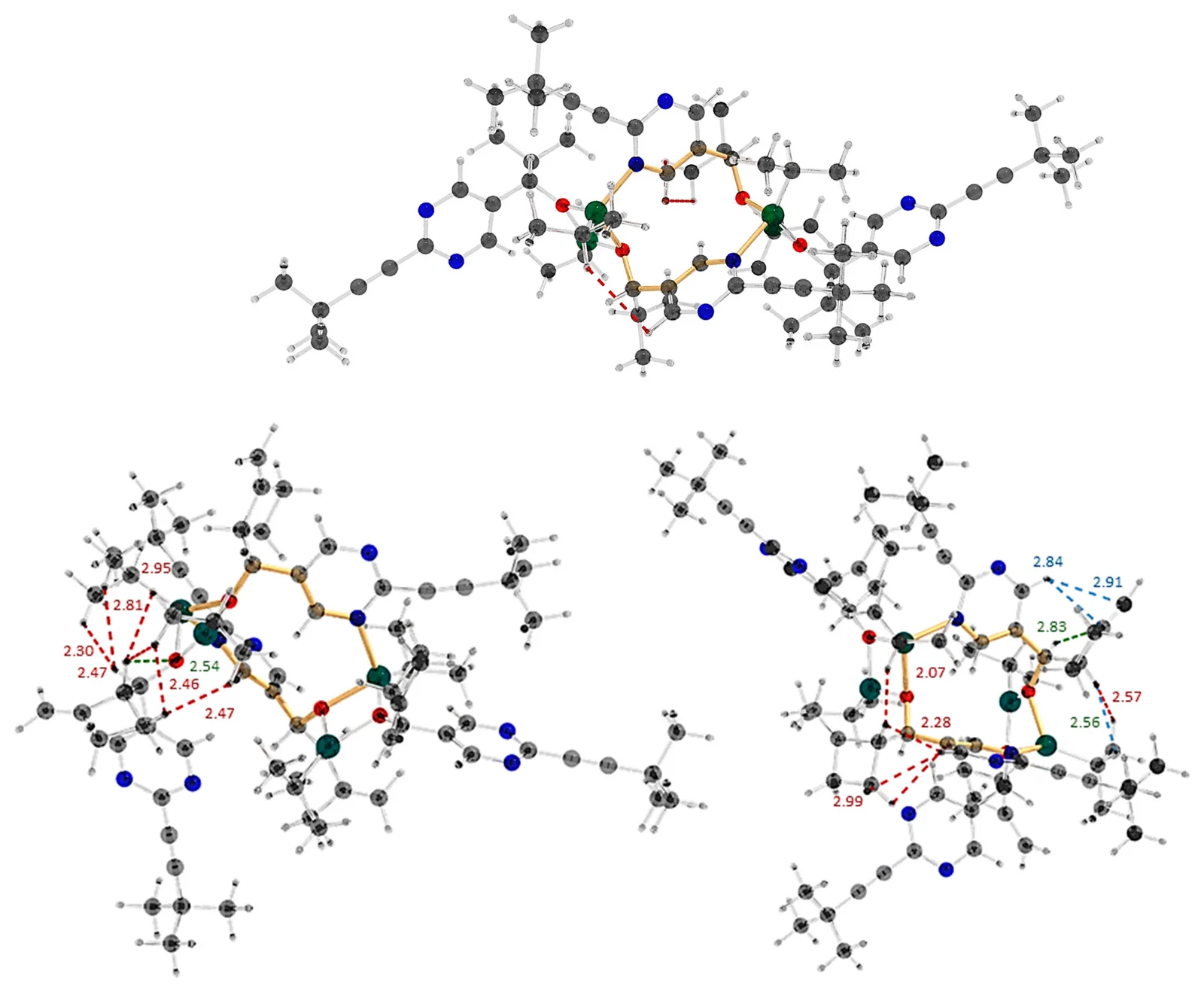
Optimized structures of tetramers (B3LYP/6-31G(d)/CPCM(toluene)). (**Top**): the amplifying catalyst of the Soai reaction [24]. (**Bottom**): structure **9** obtained after optimization of a molecule obtained after replacement of four isopropyl groups with methylenecyclobutyl groups (Scheme 6); left: backward view, right: front view. Atoms: C, black; H, gray; O, red, N, blue, Zn, turquoise. Non-covalent contacts: CH^…^HC, red; CH^…^π, blue; CH^…^O, green.

Figure 6 (top) displays the structure of the catalyst of the Soai reaction from the completely computed catalytic cycle [24]. The molecule is *C*_2_-symmetric and contains two practically orthogonal pyrimidinic rings. This arrangement creates a cavity capable of coordinating and activating the substrate that leads to further enantioselective alkylation and amplification of the enantiomeric excess.

Two alternative views on the tetramer **9** containing four methylenecyclobutane units (Scheme 6) instead of isopropyl groups are shown in Figure 6 (bottom). The structure was completely optimized on the same level of theory taking as the starting point the framework of the previously optimized catalyst (Figure 6 (top)) [24]. The new molecule keeps some important features of the true catalyst: 12-membered cycle with two practically orthogonal pyrimidine rings. However, the *C*_2_ symmetry of the tetrameric catalyst is lost since one of the pending alcoholate groups is rotated sideways, which allows its methylenecyclobutane substituent to become involved in numerous stabilizing non-covalent interactions (Figure 6 (bottom, left)). As a result, this alcoholate group is located directly below one of the orthogonal pyrimidine rings that can prevent successful docking of the substrate.

Two other methylenecyclobutyl groups use the newly acquired configuration to form numerous CH^…^HC and CH^…^π contacts with the orthogonal pyrimidine rings (Figure 6 (bottom, right; compare with only two contacts of that kind in the effective catalyst)) that are responsible for making a cavity for the substrate docking [24]. Presumably this affects the electronic properties of the pyrimidine rings and the docking becomes impossible.

This is a clear demonstration of the utmost importance of the strict fitting of numerous structural features of the substrates for the occurrence of the AAA in the Soai reaction.

## 3. Materials and Methods

### 3.1. Experimental Details

(+)-*B*-Chlorodiisopinocampheylborane 1.7 M solution (Maclin, Shanghai, China), Potassium *tert*-butoxide 98+% (Acros Organics, Geel, Belgium), *n*-Butyllithium 2.5 M solution (Sigma-Aldrich, Schnelldorf, Germany) were used as received without further purification.

All reactions were carried out using standard Schlenk techniques under an argon atmosphere in oven-dried glassware with magnetic stirring. All solvents were dried and distilled prior to use according to standard procedures. Analytical thin-layer chromatography (TLC) was carried out on Merck TLC plates (silica gel 60 F254, 0.25 mm) using UV light (254 nm) as the visualizing agent. Open column chromatography was performed on silica gel 60A (Acros Organics, 230–400 mesh, 0.040–0.063 mm). Flash column chromatography was performed using SepaFlash S-5101-0004 (irregular silica, 40–63 μm, 60 Å, 4 g). Melting points were recorded with a Boetius melting point apparatus and are uncorrected.

NMR spectra were recorded on a Bruker AVANCE 300 spectrometer at 300.13 MHz (^1^H) and 75.47 MHz (^13^C), and a Bruker AVANCE 600 spectrometer at 600.13 MHz (^1^H), 150.90 MHz (^13^C) and 192.55 MHz (^11^B), at 20 °C in CDCl_3_ or CD_2_Cl_2_. Chemical shifts (*δ*) are expressed in parts per million (ppm) and are calibrated using the residual non-deuterated solvent peak as an internal reference (CDCl_3_: *δ*_H_ 7.26, *δ*_C_ 77.16). Coupling constants (*J*) are reported in Hertz (Hz), and multiplicities are indicated as follows: s (singlet), d (doublet), t (triplet), q (quartet), m (multiplet), and br (broad).

High-resolution mass spectra (HRMS) were obtained through electrospray ionization (ESI) with positive (+) ion detection on a Bruker micrOTOF-QIII quadrupole time-of-flight mass spectrometer. Enantiomeric excess measurements were performed via HPLC analysis on an HPLC system equipped with a Daicel CHIRALPAK IC column (250 × 4.6 mm), using hexane/2-propanol (9:1) as the eluent, flow rate 1.0 mL/min, column temperature 30 °C and detection at 254 nm. Optical rotations were measured on a JASCO P-2000 polarimeter.

X-ray diffraction data for **4** were collected at 100 K on a Bruker Quest D8 diffractometer equipped with a Photon-III area-detector (shutterless φ- and ω-scan technique), using graphite-monochromatized Mo Kα radiation. The intensity data were integrated by the SAINT program (APEX3 suite) [26] and were corrected for absorption and decay using SADABS [27]. The structure was solved by direct methods using SHELXT [28] and refined on *F*^2^ using SHELXL-2019/2 [29] in the ShelXle program [30]. All non-hydrogen atoms were refined with anisotropic displacement parameters. Positions of hydrogen atoms H2 and H1B were found from the electron density-difference map; these atoms were refined with individual isotropic displacement parameters. All other hydrogen atoms were placed in ideal calculated positions and refined as riding atoms with relative isotropic displacement parameters. The Mercury program suite [31] was used for molecular graphics. Fragments of two independent molecules are disordered over two positions with the contributions of the main components 0.496(3) and 0.581(5); the disordered fragments were treated with EADP, FLAT and SAME restraints (56 restraints in total). Owing to the absence of significant anomalous scattering (light atoms only, μ = 0.073 mm^−1^, Mo Kα), the absolute structure could not be determined, and the diffraction experiment establishes the relative configuration only. Crystal data for *rac*-**4**: C_16_H_20_N_2_O, M = 256.34, orthorhombic, space group Pca21, a = 11.9650(4), b = 7.6408(3), c = 31.9709(11) Å, V = 2922.85(18) Å^3^, T = 100(2) K, Z = 8, Z’ = 2, ρcalcd = 1.165 g cm^−3^, F(000) = 1104, crystal size 0.478 × 0.421 × 0.348 mm^3^, λ = 0.71073 Å, θ range 2.548–30.557°, 131999 reflections collected, 8932 independent (Rint = 0.0432), 7901 observed [I > 2σ(I)], completeness to θ = 25.242° 99.9%, semi-empirical absorption correction (Tmax/Tmin = 0.7461/0.7053), 8932 data/56 restraints/429 parameters, GooF = 1.033, R1 = 0.0478, wR2 = 0.1211 [I > 2σ(I)], R1 = 0.0560, wR2 = 0.1279 (all data), largest difference peak/hole 0.388/−0.238 e Å^−3^. Crystallographic data have been deposited with the Cambridge Crystallographic Data Centre (CCDC 2575922) (Table S1).

### 3.2. Chemical Synthesis

2-(3,3-Dimethylbut-1-yn-1-yl)pyrimidine-5-carbaldehyde (**3**) was prepared by a known procedure [21].

**Allylpotassium reagent.** *t*-BuOK (6.4 g, 57.0 mmol) was dried in vacuo at 55 °C for 2 h in a Schlenk flask equipped with a magnetic stir bar. The flask was cooled to 0 °C, and anhydrous pentane (10 mL) and methylenecyclobutane (5.0 mL, 57.0 mmol) were added under argon. *n*-BuLi (2.5 M in hexanes, 23.0 mL, 57.5 mmol) was added dropwise via syringe and the mixture was stirred at 0 °C overnight, during which it turned dark orange with formation of a gray precipitate. The mixture was diluted with anhydrous pentane (50 mL) and filtered under argon into a separate Schlenk flask. The reagent was prepared separately on this scale for each of the two boranes described below.

**Equilibrium mixture of (cyclobut-1-en-1-ylmethyl)diisopropylborane and diisopropyl(2-methylenecyclobutyl)borane** (**1a,b**). The filtrate obtained as described above was cooled to −78 °C and *i*-Pr_2_BCl (7.7 g, 57.0 mmol) was added dropwise. The cooling bath was removed; the mixture was allowed to warm to room temperature and stirred for 2 h. The resulting white suspension was filtered under argon to remove the precipitated salts, the solid was washed with pentane (3 × 10 mL), and the combined filtrates were concentrated. Vacuum distillation of the remaining yellow liquid (bp 57–58 °C/1 mmHg) afforded **1a**,**b** as a colorless oil (4.7 g, 50%). ^11^B NMR (CD_2_Cl_2_, 192.5 MHz, 20 °C): *δ* 82.4 (br). ^1^H NMR (CD_2_Cl_2_, 600 MHz, 188 K): **1a** 5.58 (1H, =CH), 2.40 and 2.30 (4H, CH_2_CH_2_), 2.10 (2H, BCH_2_); **1b** *δ* 4.67 and 4.51 (2H, =CH_2_), 3.35 (1H, BCH), 2.76 and 2.66 (2H, CH_2_), 1.74 and 1.62 (2H, CH_2_); **1a**:**1b** = 2:1. The signals of the isopropyl groups: *δ* 1.74, 1.62, 1.22, 1.15 (CH) and 0.90 (CH_3_) (Figure S5).

**Equilibrium mixture of (cyclobut-1-en-1-ylmethyl)bis((1*****R*****,2*****R*****,3*****S*****,5*****R*****)-2,6,6-trimethylbicyclo[3.1.1]heptan-3-yl)borane and (2-methylenecyclobutyl)bis((1*****R*****,2*****R*****,3*****S*****,5*****R*****)-2,6,6-trimethylbicyclo[3.1.1]heptan-3-yl)borane** (**2a,b**). A second portion of the filtrate, prepared as described above, was cooled to −78 °C and a solution of (+)-Ipc_2_BCl (1.7 M in heptane, 33.0 mL, 56.1 mmol) was added dropwise. The reaction mixture was allowed to warm slowly to room temperature and stirred overnight. The resulting pale-yellow suspension was filtered under argon to remove the precipitated salts and the solid was washed with pentane. The filtrate was concentrated under reduced pressure to give the crude equilibrium mixture **2a**,**b** as a yellowish oil. The borane is thermally labile—attempted distillation results in loss of the isopinocampheyl groups—and the crude material retains residual solvent; it was therefore used directly in the next step without purification and without determination of the yield. Quantities given below are expressed as equivalents based on the (+)-Ipc_2_BCl employed, assuming quantitative formation. ^11^B NMR (CD_2_Cl_2_, 192.5 MHz, 20 °C): *δ* 70.2 (br) (Figure S6); the signals at 42.4 and 24.0 ppm correspond to minor oxidation products, and the broad signal centered near 0 ppm originates from the borosilicate glass of the NMR tube.

**(*****R,R*****)-(2-(3,3-Dimethylbut-1-yn-1-yl)pyrimidin-5-yl)(2-methylenecyclobutyl)me-thanol** (**4**). A 25 mL Schlenk tube equipped with a magnetic stir bar was charged with aldehyde **3** (17 mg, 0.09 mmol, 1.0 eq) and purged with argon through three vacuum-argon cycles. Anhydrous diethyl ether (10 mL) was added via syringe, and the mixture was stirred until complete dissolution. An excess of the crude borane **2a**,**b** (ca. 2 equiv) was then introduced dropwise. The reaction mixture was stirred for 10 min at ambient temperature. Subsequently, a 1% aqueous solution of KOH (7.5 mL) and a 33% aqueous solution of H_2_O_2_ (1 mL) were added carefully, and the resulting mixture was stirred vigorously for 1 h. The organic layer was separated, and the aqueous phase was extracted with ethyl acetate (3 × 10 mL). The combined organic extracts were dried over Na_2_SO_4_ and concentrated under reduced pressure. The residue was purified by consecutive chromatography: initially via automated flash chromatography (SepaBean machine U100, 4 g silica gel cartridge, flow rate 10 mL/min) using a gradient elution from hexanes to hexanes/ethyl acetate (1:1), with the product eluting after 20 column volumes (CV). The obtained material was subsequently further purified by conventional silica gel column chromatography using ethyl acetate and toluene (1:2) to afford **4**. 

**(*****R,R*****)-(2-(3,3-Dimethylbut-1-yn-1-yl)pyrimidin-5-yl)(2-methylenecyclobutyl)me-thanol** (**4**). (20 mg, 87% yield) with 89% enantiomeric excess. *R_f_* 0.32 (ethyl acetate—toluene, 1:2), mp 70–72 °C. ^1^H NMR (CDCl_3_, 300 MHz, ppm) *δ* 1.36 (s, 9H, CH_3_); 1.67–1.75 (m, 1H, CH_2_(cyc)); 1.98–2.02 (m, 1H, CH_2_(cyc)); 2.53–2.66 (m, 2H, CH_2_(cyc)); 3.24–3.30 (m, 1H, CH_cyc_); 4.73 (d, 1H, CH–O, *J* = 7.8 Hz); 4.86–4.93 (m, 2H, CH_2_=); 8.66 (s, 2H, H_Ar_). ^13^C {^1^H} NMR (CDCl_3_, 150 MHz, ppm) *δ* 19.50 (CH_2_(cyc)), 28.03 (C(CH_3_)_3_), 28.98 (CH_2_(cyc)), 30.59 (CH_3_), 50.33 (CH_cyc_), 72.38 (CH–O), 78.66, 98.16 (C≡C), 108.16 (CH_2_=), 133.50 (C_Ar_), 149.42 (C=CH_2_), 152.67 (C_Ar_), 155.68 (2CH_Ar_). The charts of 1H and 13C NMR spectra are shown in the Figure S1) HRMS (ESI) *m*/*z* 257.1648 (calcd for C_16_H_21_N_2_O [M + H]^+^ 257.1649) Figure S2). [α]_D_^20^ + 4.0 (*c* 1.0, CHCl_3_). HPLC (CHIRALPAK IC, 250 × 4.6 mm, hexane/2-PrOH 9:1, 1.0 mL/min, 30 °C, 254 nm): tR = 32.1 min (major) and 43.4 min (minor), 89% ee (Figure S4); racemic sample: tR = 30.9 and 41.5 min (Figure S3).

The racemic alcohol was prepared following the same procedure using the achiral borane **1** (23 mg, 0.14 mmol, 2.0 eq) and aldehyde **3** (13 mg, 0.07 mmol, 1.0 eq). The reaction afforded *rac*-**4** as a colorless solid (15 mg, 83% yield). *R_f_* 0.32 (ethyl acetate—toluene, 1:2); the ^1^H, ^13^C NMR and HRMS data were identical to those given above. Single crystals suitable for X-ray diffraction were grown from hexane/acetone (1:15).

**Soai reaction in the presence of 4(*****RR*****).** A Schlenk tube was charged under argon with **4**(*RR*) (89% ee; 4.1 mg, 0.016 mmol, 1 equiv) and 15 mL anhydrous toluene. Diisopropylzinc (1.0 M in toluene, 0.18 mL, 0.18 mmol, 11 equiv) was added, followed by dropwise addition of a solution of the aldehyde **3** (30 mg, 0.159 mmol, 10 equiv) in toluene at room temperature. The mixture was stirred for 2 h and was then quenched with aqueous HCl solution (0.1 M, 10 mL). After stirring for 15 min the reaction mixture was neutralized with saturated solution of NaHCO_3_ (15 mL). The organic phase was separated and the colorless aqueous layer was extracted with CHCl_3_ (3 × 10 mL). The solvent from the combined organic layers was removed in vacuo. The remaining yellow solid was purified by column chromatography (eluent acetone—chloroform, 1:4) to yield the corresponding alcohol **7** (19 mg, 51%) together with recovered **4**. HPLC analysis under the conditions given above showed that **4** had retained its enantiomeric purity, whereas **7** was obtained as a racemate.

### 3.3. Computational Details

DFT calculations were performed with Gaussian 16 Rev.C01 [32]. In the case of boranes **1a**,**b**, **2a**,**b** and their reactions with **3**, TPSSh DFT functional with def2-SVP basis set was used for geometry optimization, calculations of thermodynamics and kinetics [33,34,35,36]. For the optimization of the tetrameric alcoholate derived from **4**, which is an analog of the catalyst of the Soai reaction previously computed as a part of the whole catalytic cycle [23,24], the same computational level, B3LYP/6-31G(d) [37,38,39,40,41,42,43,44,45,46,47], was applied for consistency. In both cases, dispersion interactions were accounted for by applying the D3 correction with Becke–Johnson damping [48,49].

Cartesian coordinates are given in angstroms; absolute energies for all substances are given in hartrees. Starting coordinates of **3** were taken from CCDC 2092186. Analysis of vibrational frequencies was performed for all optimized structures. All compounds, except transition state structures, were characterized by only real frequencies. Transition states were characterized by one imaginary frequency. Non-specific solvation was introduced by using the SMD continuum model [50] (diethyl ether for boranes **1**, **2** and their reactions with **3**). For the modeling of the tetramer **9**, the CPCM continuum model (toluene) was applied [51,52].

## 4. Conclusions

In this work we have synthesized new allylboranes, diisopropyl(2-methylenecyclobutyl)borane **1** and chiral (+)-diisopinocampheyl(2-methylenecyclobutyl)borane **2**, both capable of introducing a 2-methylenecyclobutyl fragment in the reactions with carbonyl groups and their analogs via regio-, diastereo- and enantioselective allylboration. Boranes **1** and **2** consist of interconverting *endo-* and *exo-* isomers **1a**,**b** and **2a**,**b**, respectively.

As a practical example, a new chiral alcohol, (*R*,*R*)-(2-(3,3-Dimethylbut-1-yn-1-yl)pyrimidin-5-yl)(2-methylenecyclobutyl)methanol (**4**), was prepared with perfect regio- and high diastereo- and enantioselectivities. The mechanism of both reactions studied by state-of-the-art quantum chemical computations revealed the structural reasons stipulating high selectivity, i.e., the best developed network of stabilizing non-covalent interactions observed in the transition states of the preferential reaction pathways.

The alcohol **4** was tried as a catalyst in the Soai reaction but it demonstrated no catalytic activity. Computations suggested that Zn alcoholate **8** can form a tetramer **9**, but it does not keep a proper conformation for promotion of an autocatalytic reaction.

## Data Availability

The crystallographic data for compound **4** have been deposited with the Cambridge Crystallographic Data Centre (CCDC 2575922) and can be obtained free of charge via https://www.ccdc.cam.ac.uk/structures/ (accessed on 24 July 2026). All other data supporting the reported results are available in the Supplementary Materials.

## Supplementary Materials

The following supporting information can be downloaded at: https://www.mdpi.com/article/10.3390/ijms27188277/s1.
