# Supplementary material for: Regiospecific, Diastereo- and Enantioselective Allylboration with Methylenecyclobutylboranes: Experimental and Computational Study
Source: Int J Mol Sci. 2026 Sep 17;27(18):8277. doi: 10.3390/ijms27188277 (PMC13607362; doi:10.3390/ijms27188277)
Supplement: Supplementary file 1 [file ijms-27-08277-s001.zip › ijms-4561004-supplementary.pdf]

Supplementary Materials

Regiospecific, Diastereo- and Enantioselective Allylboration with Methylenecyclobutylboranes: Experimental and Computational Study

Oleg A. Mikhaylov [1], Angelina Kolchina [1], Ivan I. Murygin [1], Albert G. Nigmatov [1], Ivan S. Arteev [1,2], and Ilya D. Gridnev [1*]

[1] N. D. Zelinsky Institute of Organic Chemistry, Russian Academy of Sciences, Leninsky prosp. 47, 119991 Moscow, Russia

[2] Peoples' Friendship University of Russia (RUDN University), Miklukho-Maklay Str. 6, Moscow 117198, Russian Federation

* Correspondence: ilyaiochem@gmail.com

Content

1. Materials and Methods **3**

2. NMR Spectra of alcohol 4 .... **7**

3. HRMS data of alcohol 4 .... **8**

4. HPLC data of alcohol 4 .... **9**

5. Crystallographic data for *rac*-4 .... **10**

6. NMR Spectra of boranes 1,2 .... **11**

5. Cartesian coordinates of optimized structures **13**

1. Materials and Methods

1.1. General Information

(+)-*B*-Chlorodiisopinocampheylborane 1.7 M solution (Maclin, Shanghai, China), Potassium tert-butoxide 98+% (Acros Organics, Geel, Belgium), *n*-Butyllithium 2.5 M solution (Sigma-Aldrich, Schnelldorf, Germany) were used as received without further purification.

All reactions were carried out using standard Schlenk techniques under an argon atmosphere in oven-dried glassware with magnetic stirring. All solvents were dried and distilled prior to use according to standard procedures. Analytical thin-layer chromatography (TLC) was carried out on Merck TLC plates (silica gel 60 F254, 0.25 mm) using UV light (254 nm) as visualizing agents. Open column chromatography was performed on silica gel 60A (Acros Organics, 230–400 mesh, 0.040–0.063 mm). Flash column chromatography was performed using SepaFlash S-5101-0004 (irregular silica, 40–63 μm, 60 Å, 4 g). Melting points were recorded with a Boëtius melting point apparatus and are uncorrected.

NMR spectra were recorded on a Bruker Avance 300 spectrometer at 300.13 MHz (^{1}H) and 75.47 MHz (^{13}C), and a Bruker AVANCE 600 spectrometer at 600.13 MHz (^{1}H), 150.90 MHz (^{13}C) and 192.55 MHz (^{11}B), at 20 °C in $CDCl_3$ or CD_2Cl_2. Chemical shifts (δ) are expressed in parts per million (ppm) and are calibrated using the residual non-deuterated solvent peak as an internal reference ($CDCl_3$: δ_H 7.26, δ_C 77.16). Coupling constants (*J*) are reported in Hertz (Hz), and multiplicities are indicated as follows: s (singlet), d (doublet), t (triplet), q (quartet), m (multiplet), and br (broad).

High-resolution mass spectra (HRMS) were obtained through electrospray ionization (ESI) with positive (+) ion detection on a Bruker micrOTOF-QIII quadrupole time-of-flight mass spectrometer. Enantiomeric excess measurements were performed via HPLC analysis on an HPLC system equipped with a Daicel CHIRALPAK IC column, (250 × 4.6 mm) using hexane/2-propanol (9:1) as the eluent, flow rate 1.0 mL/min, column temperature 30 °C and detection at 254 nm. Optical rotations were measured on a JASCO P-2000 polarimeter.

X-ray diffraction data for **4** were collected at 100 K on a Bruker Quest D8 diffractometer equipped with a Photon-III area-detector (shutterless φ- and ω-scan technique), using graphite-monochromatized Mo Kα radiation. The intensity data were integrated by the SAINT program (APEX3 suite) [1] and were corrected for absorption and decay using SADABS [2]. The structure was solved by direct methods using SHELXT [3] and refined on F^2 using SHELXL-2019/2 [4] in the ShelXle program [5]. All non-hydrogen atoms were refined with anisotropic displacement parameters. Positions of hydrogen atoms H2 and H1B were found from the electron density-difference map; these atoms were refined with individual isotropic displacement parameters. All other hydrogen atoms were placed in ideal calculated positions and refined as riding atoms with relative isotropic displacement parameters. The Mercury program suite [6] was used for molecular graphics. Fragments of two independent molecules are disordered over two positions with the contributions of the main components 0.496(3) and 0.581(5); the disordered fragments were treated with EADP, FLAT and SAME restraints (56 restraints in total). Owing to the absence of significant anomalous scattering (light atoms only, μ = 0.073 mm−1, Mo Kα) the absolute structure could not be determined, and the diffraction experiment establishes the relative configuration only. Crystal data for *rac*-**4**: $C_{16}H_{20}N_2O$, M = 256.34, orthorhombic, space group Pca21, a = 11.9650(4), b = 7.6408(3), c = 31.9709(11) Å, V = 2922.85(18) Å3, T = 100(2) K, Z = 8, Z' = 2, ϱcalcd = 1.165 g cm−3, F(000) = 1104, crystal size 0.478 × 0.421 × 0.348 mm3, λ = 0.71073 Å, θ range 2.548–30.557°, 131999 reflections collected, 8932 independent (Rint = 0.0432), 7901 observed [I > 2σ(I)], completeness to θ = 25.242° 99.9%, semi-empirical absorption correction (Tmax/Tmin = 0.7461/0.7053), 8932 data / 56

restraints / 429 parameters, GooF = 1.033, R1 = 0.0478, wR2 = 0.1211 [$I > 2\sigma(I)$], R1 = 0.0560, wR2 = 0.1279 (all data), largest difference peak/hole 0.388/−0.238 e Å−3. Crystallographic data have been deposited with the Cambridge Crystallographic Data Centre (CCDC 2575922).

1.2. Chemical Synthesis

2-(3,3-Dimethylbut-1-yn-1-yl)pyrimidine-5-carbaldehyde (**3**) was prepared by a known procedure [7].

Allylpotassium reagent. *t*-BuOK (6.4 g, 57.0 mmol) was dried in vacuo at 55 °C for 2 h in a Schlenk flask equipped with a magnetic stir bar. The flask was cooled to 0 °C, and anhydrous pentane (10 mL) and methylenecyclobutane (5.0 mL, 57.0 mmol) were added under argon. *n*-BuLi (2.5 M in hexanes, 23.0 mL, 57.5 mmol) was added dropwise via syringe and the mixture was stirred at 0 °C overnight, during which it turned dark orange with formation of a grey precipitate. The mixture was diluted with anhydrous pentane (50 mL) and filtered under argon into a separate Schlenk flask. The reagent was prepared separately on this scale for each of the two boranes described below.

Equilibrium mixture of (cyclobut-1-en-1-ylmethyl)diisopropylborane and diisopropyl(2-methylenecyclobutyl)borane (1a,b). The filtrate obtained as described above was cooled to −78 °C and *i*-Pr_2BCl (7.7 g, 57.0 mmol) was added dropwise. The cooling bath was removed; the mixture was allowed to warm to room temperature and stirred for 2 h. The resulting white suspension was filtered under argon to remove the precipitated salts, the solid was washed with pentane (3 × 10 mL), and the combined filtrates were concentrated. Vacuum distillation of the remaining yellow liquid (bp 57–58 °C / 1 mmHg) afforded **1a,b** as a colorless oil (4.7 g, 50%). ^{11}B NMR (CD_2Cl_2, 192.5 MHz, 20 °C): δ 82.4 (br). ^{1}H NMR (CD_2Cl_2, 600 MHz, 188 K): **1a** 5.58 (1H, =CH), 2.40 and 2.30 (4H, CH_2CH_2), 2.10 (2H, BCH_2); **1b** δ 4.67 and 4.51 (2H, =CH_2), 3.35 (1H, BCH), 2.76 and 2.66 (2H, CH_2), 1.74 and 1.62 (2H, CH_2); **1a**:**1b** = 2:1. The signals of the isopropyl groups: δ 1.74, 1.62, 1.22, 1.15 (CH) and 0.90 (CH_3).

Equilibrium mixture of (cyclobut-1-en-1-ylmethyl)bis((1*R*,2*R*,3*S*,5*R*)-2,6,6-trimethylbicyclo[3.1.1]heptan-3-yl)borane and (2-methylenecyclobutyl)bis((1*R*,2*R*,3*S*,5*R*)-2,6,6-trimethylbicyclo[3.1.1]heptan-3-yl)borane (2a,b). A second portion of the filtrate, prepared as described above, was cooled to −78 °C and a solution of (+)-Ipc_2BCl (1.7 M in heptane, 33.0 mL, 56.1 mmol) was added dropwise. The reaction mixture was allowed to warm slowly to room temperature and stirred overnight. The resulting pale-yellow suspension was filtered under argon to remove the precipitated salts and the solid was washed with pentane. The filtrate was concentrated under reduced pressure to give the crude equilibrium mixture **2a,b** as a yellowish oil. The borane is thermally labile — attempted distillation results in loss of the isopinocampheyl groups — and the crude material retains residual solvent; it was therefore used directly in the next step without purification and without determination of the yield. Quantities given below are expressed as equivalents based on the (+)-Ipc_2BCl employed, assuming quantitative formation. ^{11}B NMR (CD_2Cl_2, 192.5 MHz, 20 °C): δ 70.2 (br); the signals at 42.4 and 24.0 ppm correspond to minor oxidation products, and the broad signal centred near 0 ppm originates from the borosilicate glass of the NMR tube.

(*R*,*R*)-(2-(3,3-Dimethylbut-1-yn-1-yl)pyrimidin-5-yl)(2-methylenecyclobutyl)me-thanol (**4**) A 25-mL Schlenk tube equipped with a magnetic stir bar was charged with aldehyde **3** (17 mg, 0.09 mmol, 1.0 eq) and purged with argon through three vacuum-argon cycles. Anhydrous diethyl ether (10 mL) was added via syringe, and the mixture was stirred until complete dissolution. An excess of the crude borane **2a,b** (ca. 2 equiv) was then introduced dropwise. The reaction mixture was stirred for 10 min at ambient temperature. Subsequently, a 1% aqueous solution of KOH (7.5 mL) and a 33% aqueous solution of H_2O_2 (1 mL) were added carefully, and the resulting mixture was stirred vigorously for 1 hour. The organic layer was separated, and the aqueous phase was extracted with ethyl acetate (3 × 10 mL). The combined organic extracts were dried over Na_2SO4 and concentrated under reduced pressure. The residue was purified by consecutive

chromatography: initially via automated flash chromatography (SepaBean machine U100, 4 g silica gel cartridge, flow rate 10 mL/min) using a gradient elution from hexanes to hexanes/ethyl acetate (1:1), with the product eluting after 20 column volumes (CV). The obtained material was subsequently further purified by conventional silica gel column chromatography using ethyl acetate and toluene (1:2) to afford **4**.

(*R*,*R*)-(2-(3,3-Dimethylbut-1-yn-1-yl)pyrimidin-5-yl)(2-methylenecyclobutyl)me-thanol (**4**) (20 mg, 87% yield) with 89% enantiomeric excess. R_f 0.32 (ethyl acetate – toluene, 1:2), mp 70–72 °C. ^{1}H NMR ($CDCl_3$, 300 MHz, ppm) δ 1.36 (s, 9H, CH_3); 1.67–1.75 (m, 1H, CH_2(cyc)); 1.98–2.02 (m, 1H, CH_2(cyc)); 2.53–2.66 (m, 2H, CH_2(cyc)); 3.24–3.30 (m, 1H, CH_{cyc}); 4.73 (d, 1H, CH–O, J = 7.8 Hz); 4.86–4.93 (m, 2H, CH_2=); 8.66 (s, 2H, H_{Ar}). ^{13}C {^{1}H} NMR ($CDCl_3$, 150 MHz, ppm) δ 19.50 (CH_2(cyc)), 28.03 ($\underline{C}(CH_3)_3$), 28.98 (CH_2(cyc)), 30.59 (CH_3), 50.33 (CH_{cyc}), 72.38 (CH–O), 78.66, 98.16 (C≡C), 108.16 (CH_2=), 133.50 (C_{Ar}), 149.42 ($\underline{C}=CH_2$), 152.67 (C_{Ar}), 155.68 (2CH_{Ar}). HRMS (ESI) m/z 257.1648 (calcd for $C_{16}H_{21}N_2O$ [M + H]$^+$ 257.1649). $[\alpha]_D^{20}$ +4.0 (c 1.0, $CHCl_3$). HPLC (CHIRALPAK IC, 250 × 4.6 mm, hexane/2-PrOH 9:1, 1.0 mL/min, 30 °C, 254 nm): tR = 32.1 min (major) and 43.4 min (minor), 89% ee; racemic sample: tR = 30.9 and 41.5 min.

The racemic alcohol was prepared following the same procedure using the achiral borane **1** (23 mg, 0.14 mmol, 2.0 eq) and aldehyde **3** (13 mg, 0.07 mmol, 1.0 eq). The reaction afforded *rac*-**4** as a colorless solid (15 mg, 83% yield). Rf 0.32 (ethyl acetate – toluene, 1:2); the ^{1}H, ^{13}C NMR and HRMS data were identical to those given above. Single crystals suitable for X-ray diffraction were grown from hexane/acetone (1:15).

Soai reaction in the presence of 4(*RR*). A Schlenk tube was charged under argon with **4**(*RR*) (89% ee; 4.1 mg, 0.016 mmol, 1 equiv) and 15 ml anhydrous toluene. Diisopropylzinc (1.0 M in toluene, 0.18 mL, 0.18 mmol, 11 equiv) was added, followed by dropwise addition of a solution of the aldehyde **3** (30 mg, 0.159 mmol, 10 equiv) in toluene at room temperature. The mixture was stirred for 2 h and was then quenched with aqueous HCl solution (0.1 M, 10 mL). After stirring for 15 minutes the reaction mixture was neutralized with saturated solution of $NaHCO_3$ (15 mL). The organic phase was separated and the colorless aqueous layer was extracted with $CHCl_3$ (3 × 10 mL). The solvent from the combined organic layers was removed *in vacuo*. The remaining yellow solid was purified by column chromatography (eluent acetone – chloroform, 1 : 4) to yield the corresponding alcohol **7** (19 mg, 51%) together with recovered **4**. HPLC analysis under the conditions given above showed that **4** had retained its enantiomeric purity, whereas **7** was obtained as a racemate.

1.3. Computational Details

DFT calculations were performed with Gaussian 16 Rev.C01 [8]. In the case of boranes **1a**,**b**; **2a**,**b** and their reactions with **3**, TPSSh DFT functional with def2-SVP basis set was used for geometry optimization, calculations of thermodynamics and kinetics [9–12]. For the optimization of the tetrameric alcoholate derived from **4**, that is an analogue of the catalyst of the Soai reaction previously computed as a part of the whole catalytic cycle [13,14], the same computational level, B3LYP/6-31G(d) [15–25] was applied for consistency. In both cases dispersion interactions were accounted for by applying the D3 correction with Becke–Johnson damping [26,27].

Cartesian coordinates are given in angstroms; absolute energies for all substances are given in hartrees. Starting coordinates of **3** were taken from CCDC 2092186. Analysis of vibrational frequencies was performed for all optimized structures. All compounds, except transition state structures, were characterized by only real frequencies. TS were characterized by one imaginary frequency. Non-specific solvation was introduced by using the SMD continuum model [28] (diethyl ether for boranes **1**, **2** and their reactions with **3**). For the modeling of the tetramer **9** the CPCM continuum model (toluene) was applied [29,30].

1. Bruker. APEX-III; Bruker AXS Inc.: Madison, WI, USA, 2019.
2. Krause, L.; Herbst-Irmer, R.; Sheldrick, G.M.; Stalke, D. Comparison of Silver and Molybdenum Microfocus X-ray Sources for Single-Crystal Structure Determination. *J. Appl. Cryst.* **2015**, *48*, 3–10.
3. Sheldrick, G.M. SHELXT—Integrated Space-Group and Crystal-Structure Determination. *Acta Cryst.* **2015**, *A71*, 3–8.
4. Sheldrick, G.M. Crystal Structure Refinement with SHELXL. *Acta Cryst.* **2015**, *C71*, 3–8.

5. Hübschle, C.B.; Sheldrick, G.M.; Dittrich, B. ShelXle: A Qt Graphical User Interface for SHELXL. *J. Appl. Cryst.* **2011**, *44*, 1281–1284.
6. Macrae, C.F.; Sovago, I.; Cottrell, S.J.; Galek, P.T.A.; McCabe, P.; Pidcock, E.; Platings, M.; Shields, G.P.; Stevens, J.S.; Towler, M.; Wood, P.A. Mercury 4.0: From Visualization to Analysis, Design and Prediction. *J. Appl. Cryst.* **2020**, *53*, 226–235.
7. Shibata, T.; Yonekubo, S.; Soai, K. Practically Perfect Asymmetric Autocatalysis with (2-Alkynyl-5-pyrimidyl)alkanols. *Angew. Chem. Int. Ed.* **1999**, *38*, 659–661.
8. Frisch, M.J.; Trucks, G.W.; Schlegel, H.B.; Scuseria, G.E.; Robb, M.A.; Cheeseman, J.R.; Scalmani, G.; Barone, V.; Petersson, G.A.; et al. Gaussian 16, Revision C.01; Gaussian, Inc.: Wallingford, CT, USA, 2019.
9. Tao, J.; Perdew, J.P.; Staroverov, V.N.; Scuseria, G.E. Climbing the Density Functional Ladder: Nonempirical Meta-Generalized Gradient Approximation Designed for Molecules and Solids. *Phys. Rev. Lett.* **2003**, *91*, 146401.
10. Staroverov, V.N.; Scuseria, G.E.; Tao, J.; Perdew, J.P. Comparative Assessment of a New Nonempirical Density Functional: Molecules and Hydrogen-Bonded Complexes. *J. Chem. Phys.* **2003**, *119*, 12129–12137.
11. Rydberg, P.; Olsen, L. The Accuracy of Geometries for Iron Porphyrin Complexes from Density Functional Theory. *J. Phys. Chem. A* **2009**, *113*, 11949–11953.
12. Jensen, K.P. Bioinorganic Chemistry Modeled with the TPSSh Density Functional. *Inorg. Chem.* **2008**, *47*, 10357–10365.
13. Gridnev, I.D.; Serafimov, J.M.; Quiney, H.; Brown, J.M. Reflections on Spontaneous Asymmetric Synthesis by Amplifying Autocatalysis. *Org. Biomol. Chem.* **2003**, *1*, 3811–3819.
14. Gridnev, I.D.; Vorob'ev, A.Kh. Quantification of Sophisticated Equilibria in the Reaction Pool and Amplifying Catalytic Cycle of Soai Reaction. *ACS Catal.* **2012**, *2*, 2137–2149.
15. Becke, A.D. A New Mixing of Hartree–Fock and Local Density-Functional Theories. *J. Chem. Phys.* **1993**, *98*, 1372–1377.
16. Becke, A.D. Density-Functional Thermochemistry. III. The Role of Exact Exchange. *J. Chem. Phys.* **1993**, *98*, 5648–5652.
17. Lee, C.; Yang, W.; Parr, R.G. Development of the Colle–Salvetti Correlation-Energy Formula into a Functional of the Electron Density. *Phys. Rev. B* **1988**, *37*, 785–789.
18. Tirado-Rives, J.; Jorgensen, W.L. Performance of B3LYP Density Functional Methods for a Large Set of Organic Molecules. *J. Chem. Theory Comput.* **2008**, *4*, 297–306.
19. Ditchfield, R.; Hehre, W.J.; Pople, J.A. Self-Consistent Molecular-Orbital Methods. IX. An Extended Gaussian-Type Basis for Molecular-Orbital Studies of Organic Molecules. *J. Chem. Phys.* **1971**, *54*, 724–728.
20. Hehre, W.J.; Ditchfield, R.; Pople, J.A. Self-Consistent Molecular Orbital Methods. XII. Further Extensions of Gaussian-Type Basis Sets for Use in Molecular Orbital Studies of Organic Molecules. *J. Chem. Phys.* **1972**, *56*, 2257–2261.
21. Hariharan, P.C.; Pople, J.A. The Influence of Polarization Functions on Molecular Orbital Hydrogenation Energies. *Theor. Chim. Acta* **1973**, *28*, 213–222.
22. Gordon, M.S. The Isomers of Silacyclopropane. *Chem. Phys. Lett.* **1980**, *76*, 163–168.
23. Francl, M.M.; Pietro, W.J.; Hehre, W.J.; Binkley, J.S.; Gordon, M.S.; DeFrees, D.J.; Pople, J.A. Self-Consistent Molecular Orbital Methods. XXIII. A Polarization-Type Basis Set for Second-Row Elements. *J. Chem. Phys.* **1982**, *77*, 3654–3665.
24. Rassolov, V.A.; Pople, J.A.; Ratner, M.A.; Windus, T.L. 6-31G* Basis Set for Atoms K through Zn. *J. Chem. Phys.* **1998**, *109*, 1223–1229.
25. Hariharan, P.C.; Pople, J.A. Accuracy of AH_n Equilibrium Geometries by Single Determinant Molecular Orbital Theory. *Mol. Phys.* **1974**, *27*, 209–214.
26. Grimme, S.; Antony, J.; Ehrlich, S.; Krieg, H. A Consistent and Accurate ab Initio Parametrization of Density Functional Dispersion Correction (DFT-D) for the 94 Elements H-Pu. *J. Chem. Phys.* **2010**, *132*, 154104.
27. Grimme, S.; Ehrlich, S.; Goerigk, L. Effect of the Damping Function in Dispersion Corrected Density Functional Theory. *J. Comput. Chem.* **2011**, *32*, 1456–1465.
28. Marenich, A.V.; Cramer, C.J.; Truhlar, D.G. Universal Solvation Model Based on Solute Electron Density and on a Continuum Model of the Solvent Defined by the Bulk Dielectric Constant and Atomic Surface Tensions. *J. Phys. Chem. B* **2009**, *113*, 6378–6396.
29. Barone, V.; Cossi, M. Quantum Calculation of Molecular Energies and Energy Gradients in Solution by a Conductor Solvent Model. *J. Phys. Chem. A* **1998**, *102*, 1995–2001.
30. Cossi, M.; Rega, N.; Scalmani, G.; Barone, V. Energies, Structures, and Electronic Properties of Molecules in Solution with the C-PCM Solvation Model. *J. Comput. Chem.* **2003**, *24*, 669–681.

2. NMR Spectra of alcohol 4

a)

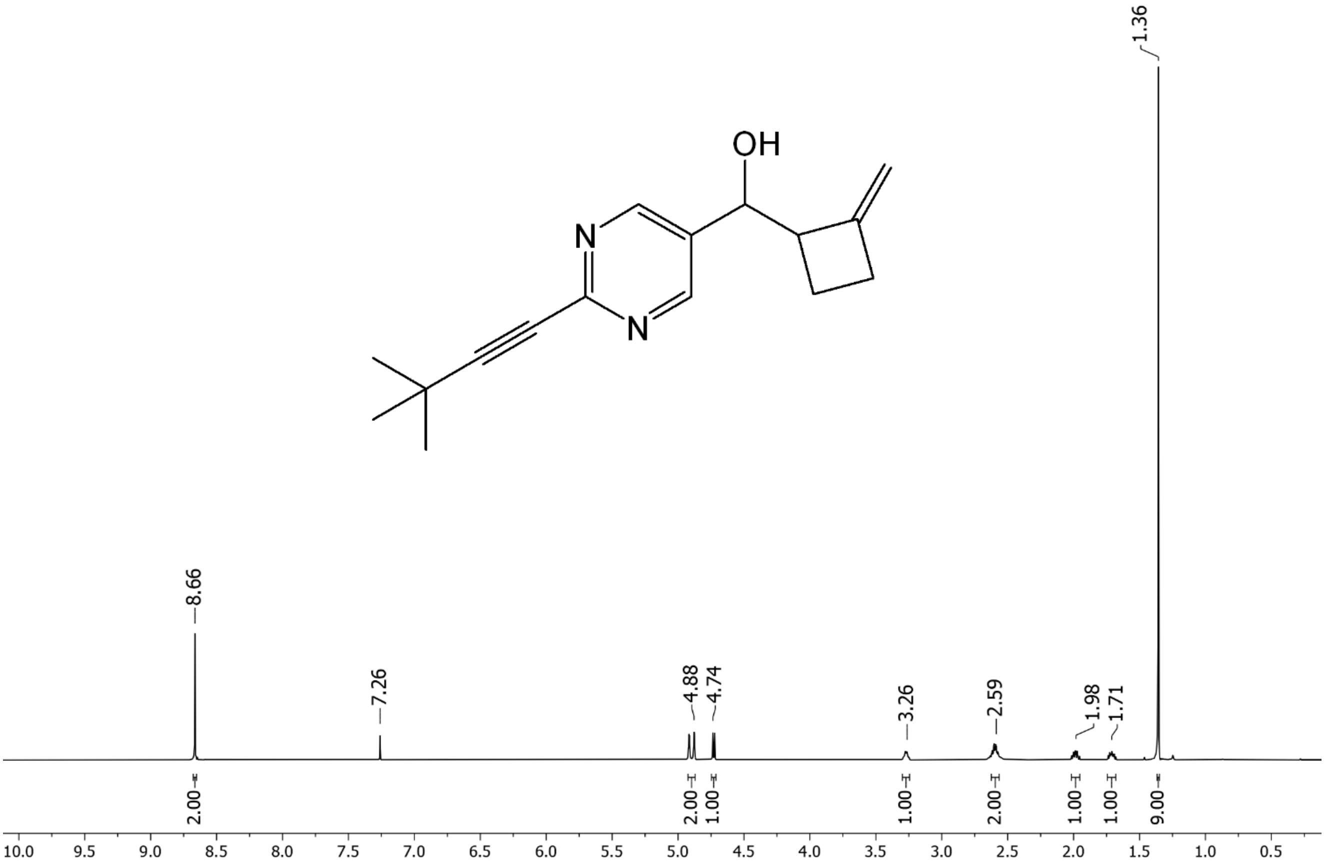

b)

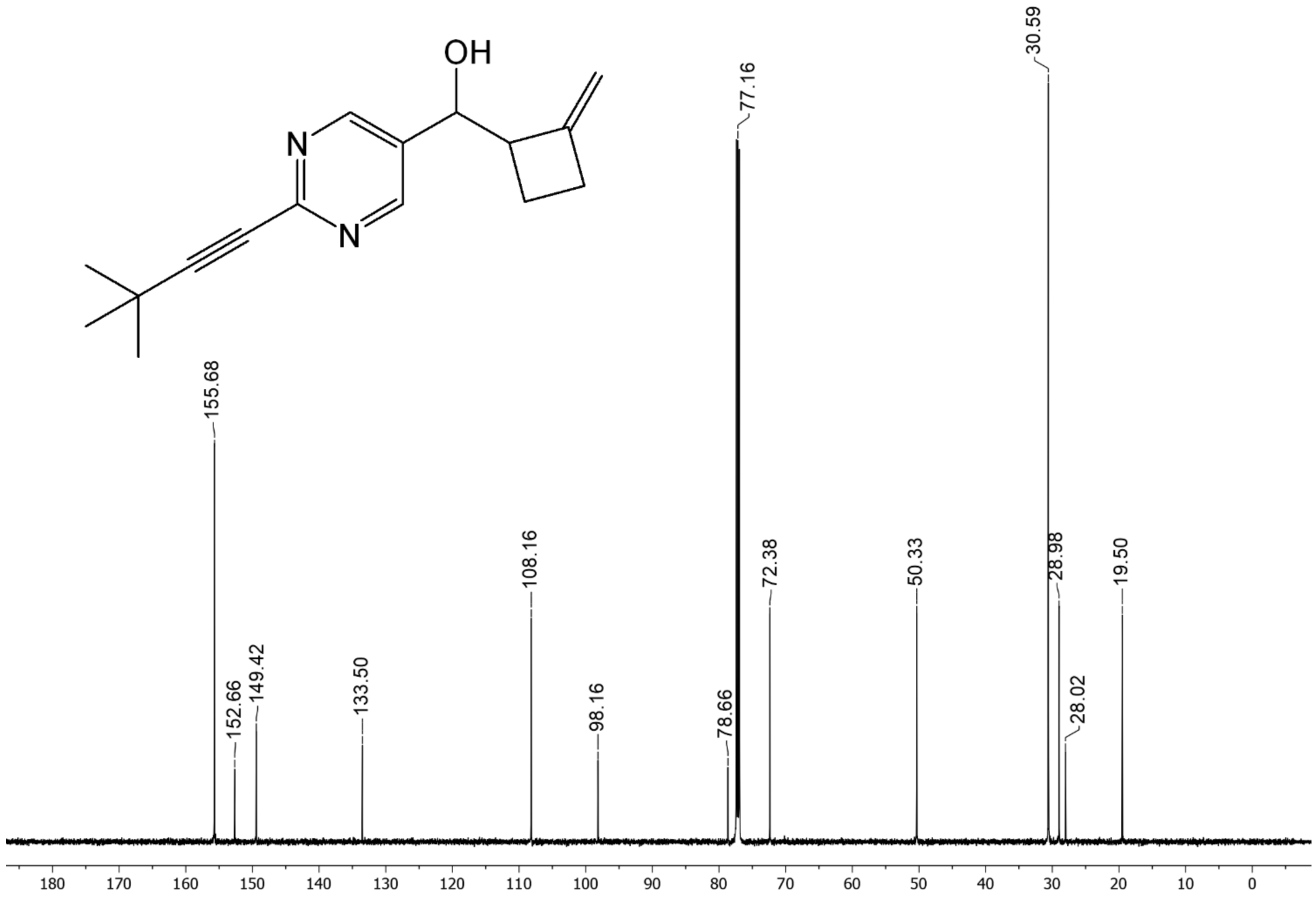

Figure S1. ^{1}H (a) and $^{13}C\{^{1}H\}$ (b) NMR spectra of compound **4** ($CDCl_3$).

3. HRMS data of alcohol 4

Acquisition Parameter

Source Type	ESI	Ion Polarity	Positive	Set Nebulizer	1.0 Bar
Focus	Active			Set Dry Heater	200 °C
Scan Begin	50 m/z	Set Capillary	4500 V	Set Dry Gas	4.0 l/min
Scan End	1800 m/z	Set End Plate Offset	-500 V	Set Divert Valve	Waste

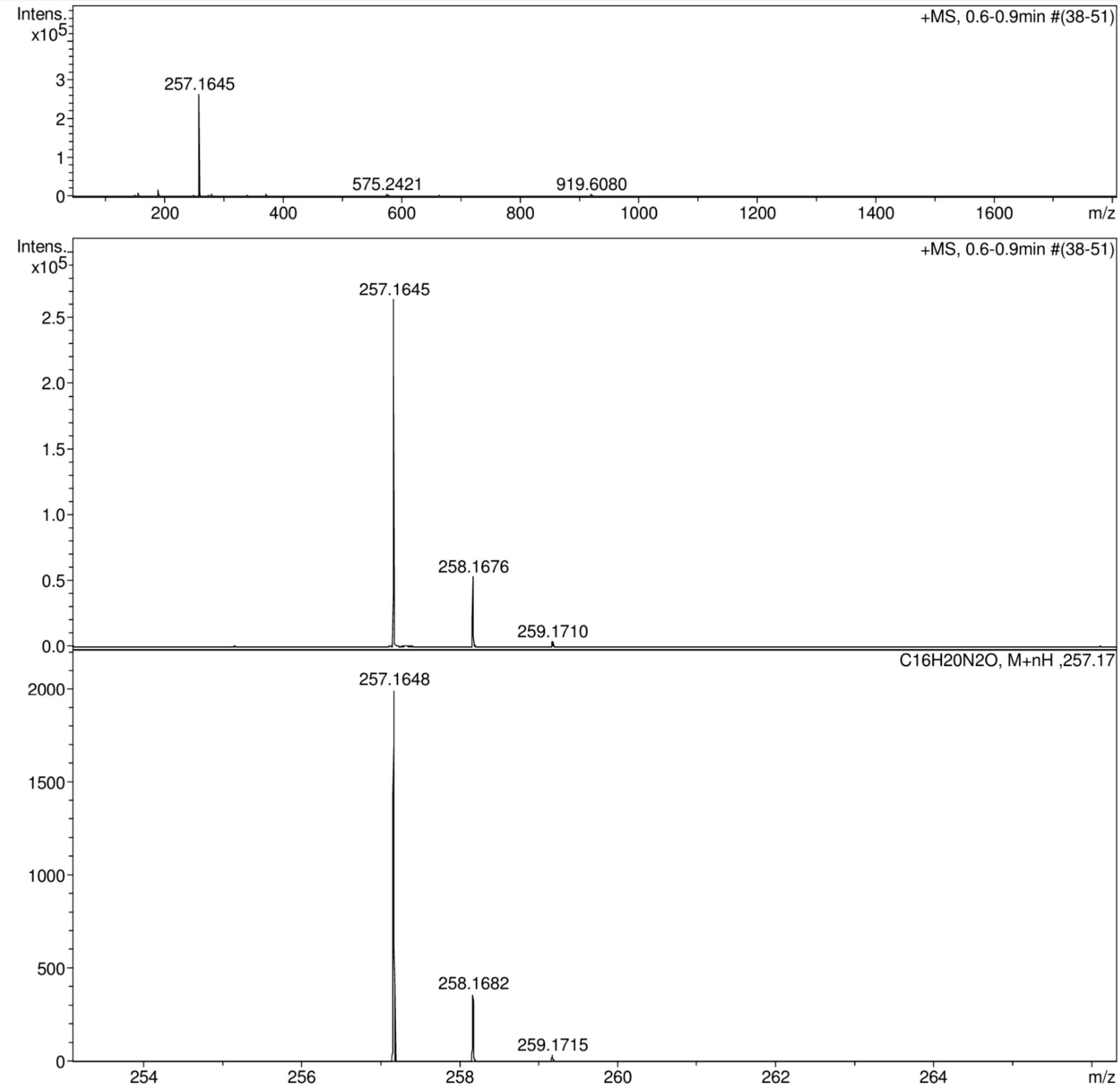

Figure S2. HRMS data of compound **4**.

4. HPLC data of alcohol 4

Conditions: hexane : 2-propanol = 9:1,
Flow rate = 1.0 mL/min, λ = 254 nm, CHIRALPAK IC.

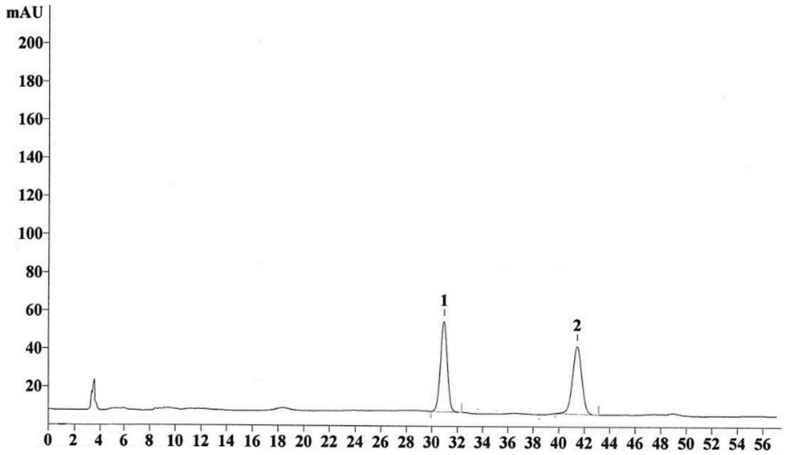

	Peak 1	Peak 2
Retention Time (min)	30.86	41.54
Relative Area (%)	49.22	50.78

Figure S3. HPLC chromatogram for racemic compound *rac*-**4**.

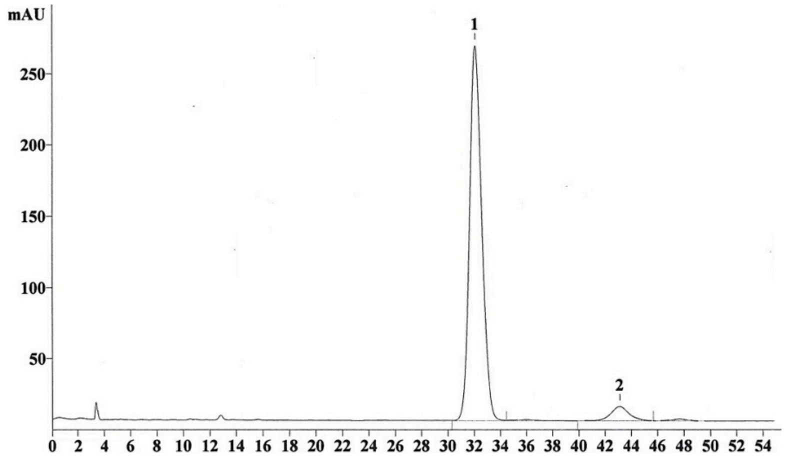

	Peak 1	Peak 2
Retention Time (min)	32.08	43.37
Relative Area (%)	94.64	5.36

Figure S4. HPLC chromatogram for chiral compound **4**(*RR*).

5. Crystallographic data for *rac*-4

The asymmetric unit contains one **4**(*R*,*R*) and one **4**(*S*,*S*) molecule. Fragments of both independent molecules are disordered over two positions with the contributions of the main components 0.496(3) and 0.581(5); the disordered fragments were treated with EADP, FLAT and SAME restraints. Owing to the absence of significant anomalous scattering (light atoms only, $\mu = 0.073\ mm^{-1}$, Mo Kα) the absolute structure could not be determined and the refined Flack parameter, −0.3(3), is not significant; the diffraction experiment establishes the relative configuration only.

Table S1. Crystal data and structure refinement for *rac*-**4**.

Empirical formula	$C_{16}H_{20}N_2O$
Formula weight	256.34
Temperature	100(2) K
Wavelength	0.71073 Å
Crystal system, space group	orthorhombic, $Pca2_1$
Unit cell dimensions	a = 11.9650(4) Å, b = 7.6408(3) Å, c = 31.9709(11) Å; $\alpha = \beta = \gamma = 90°$
Volume	2922.85(18) $Å^3$
Z, Z'	8, 2
Calculated density	1.165 g cm^{-3}
Absorption coefficient	0.073 mm^{-1}
F(000)	1104
Crystal size	0.478 × 0.421 × 0.348 mm^3
θ range for data collection	2.548–30.557°
Index ranges	$-17 \le h \le 17$, $-10 \le k \le 10$, $-45 \le l \le 45$
Reflections collected / independent	131999 / 8932 [R_{int} = 0.0432]
Observed reflections [$I > 2\sigma(I)$]	7901
Completeness to θ = 25.242°	99.9%
Absorption correction	semi-empirical from equivalents
Max. / min. transmission	0.7461 / 0.7053
Refinement method	full-matrix least-squares on F^2
Data / restraints / parameters	8932 / 56 / 429
Goodness-of-fit on F^2	1.033
Final R indices [$I > 2\sigma(I)$]	$R1$ = 0.0478, $wR2$ = 0.1211
R indices (all data)	$R1$ = 0.0560, $wR2$ = 0.1279
Largest diff. peak / hole	0.388 / −0.238 e $Å^{-3}$
CCDC deposition number	2575922

6. NMR Spectra of boranes 1,2

a)

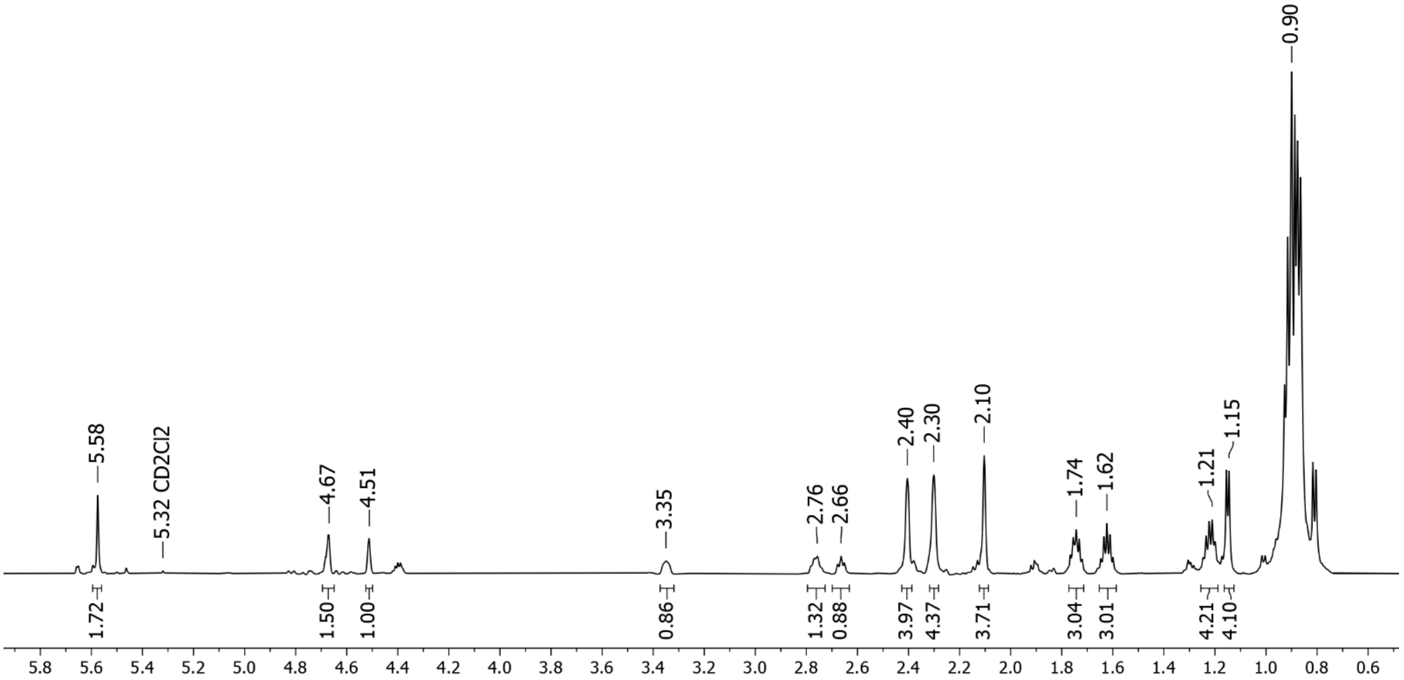

b)

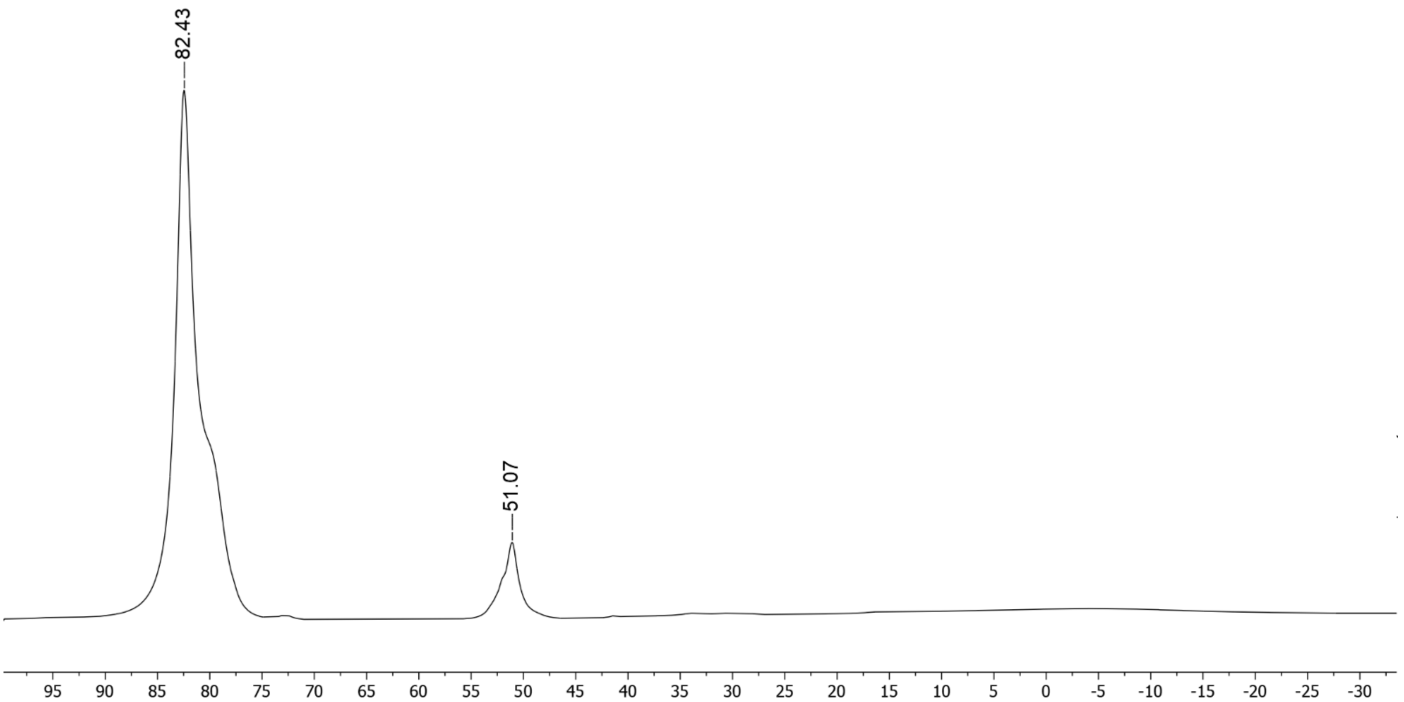

Figure S5. ^{1}H (a) and ^{11}B (b) NMR spectra of borane **1** (CD_2Cl_2).

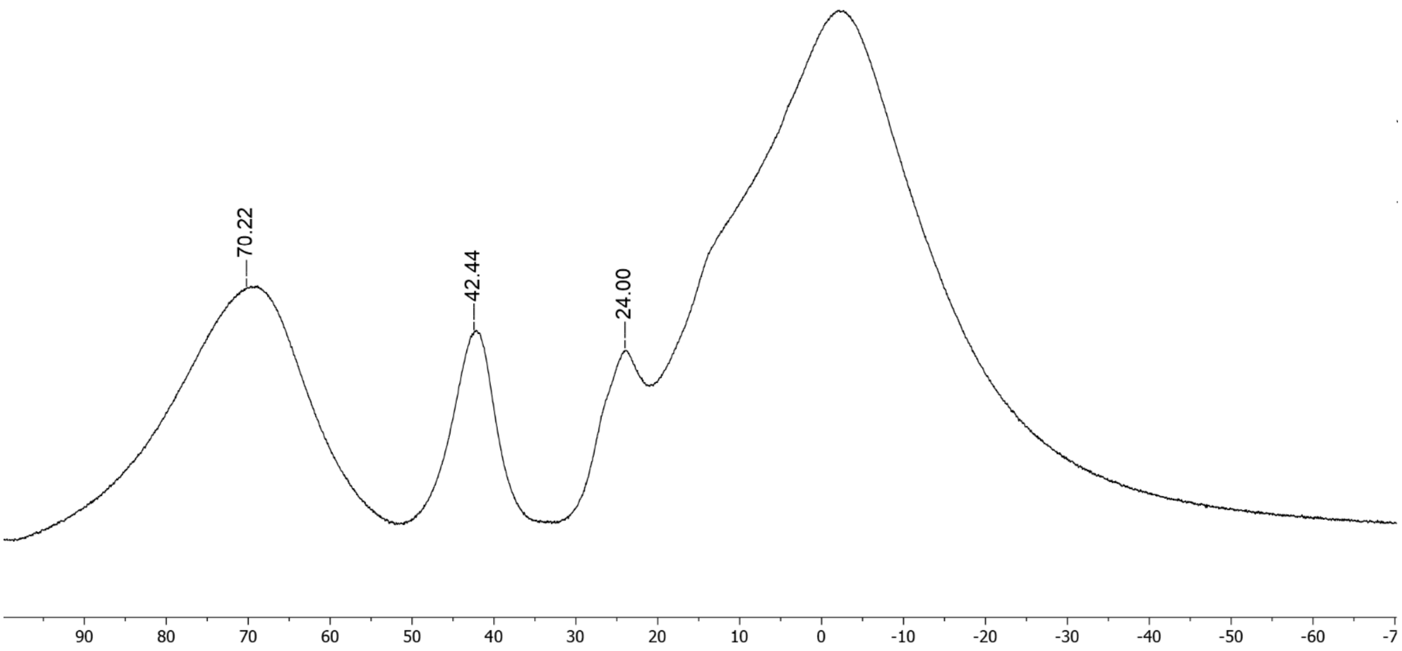

Figure S6. ^{11}B NMR spectra of borane **2** (CD_2Cl_2). The signals at 42.4 and 24.0 ppm correspond to minor oxidation products, and the broad signal centred near 0 ppm originates from the borosilicate glass of the NMR tube.

5. Cartesian coordinates of optimized structures

1a

Sum of electronic and zero-point Energies= -456.065656
Sum of electronic and thermal Energies= -456.050141
Sum of electronic and thermal Enthalpies= -456.049197
Sum of electronic and thermal Free Energies= -456.109451

Standard orientation:

Center Number	Atomic Number	Atomic Type	Coordinates (Angstroms) X	Y	Z
1	5	0	-0.819974	0.008738	0.113806
2	6	0	0.463130	0.184276	1.046647
3	1	0	0.463245	1.163720	1.562355
4	1	0	0.458594	-0.572834	1.856618
5	6	0	1.773775	0.058191	0.321375
6	6	0	3.196305	0.257741	0.825392
7	6	0	2.177955	-0.246829	-0.932666
8	6	0	3.652430	-0.097616	-0.625705
9	1	0	3.525767	-0.449676	1.606579
10	1	0	3.442763	1.282205	1.155191
11	1	0	1.644516	-0.510753	-1.851962
12	1	0	4.259741	-1.016061	-0.708720
13	1	0	4.177849	0.714538	-1.158255
14	6	0	-1.616714	1.305444	-0.362189
15	6	0	-1.290265	-1.438150	-0.361573
16	1	0	-2.287527	1.045690	-1.201806
17	6	0	-0.610809	-2.636034	0.319940
18	1	0	-0.888372	-3.587840	-0.166266
19	1	0	0.487510	-2.558172	0.290961
20	1	0	-0.912164	-2.714781	1.379284
21	6	0	-2.816603	-1.645997	-0.425058
22	1	0	-3.251030	-1.696151	0.588359
23	1	0	-3.330444	-0.837868	-0.968735
24	1	0	-3.066741	-2.594917	-0.930543
25	6	0	-0.729250	2.476582	-0.817203
26	1	0	-0.050795	2.812800	-0.014857
27	1	0	-0.105400	2.205187	-1.684599
28	1	0	-1.343214	3.346887	-1.110621
29	6	0	-2.520363	1.740835	0.817724
30	1	0	-3.132315	2.616323	0.537112
31	1	0	-3.212516	0.942157	1.131599
32	1	0	-1.921955	2.028340	1.699415
33	1	0	-0.935186	-1.413144	-1.420239

1b

Sum of electronic and zero-point Energies= -456.065143
Sum of electronic and thermal Energies= -456.049719
Sum of electronic and thermal Enthalpies= -456.048775
Sum of electronic and thermal Free Energies= -456.107780

Standard orientation:

Center Number	Atomic Number	Atomic Type	Coordinates (Angstroms) X	Y	Z
1	5	0	-0.446383	0.037691	-0.133509
2	6	0	2.279757	-1.224352	1.387525
3	1	0	3.196124	-1.042049	1.959540
4	1	0	1.611387	-2.009664	1.753587
5	6	0	2.012318	-0.527735	0.276349
6	6	0	2.762534	0.549529	-0.485118
7	6	0	0.882585	-0.575505	-0.762479
8	6	0	1.765422	0.359556	-1.658354
9	1	0	3.824017	0.337722	-0.694955
10	1	0	2.702292	1.542500	-0.008007
11	1	0	0.718933	-1.583198	-1.179524
12	1	0	2.207859	-0.180508	-2.508481
13	1	0	1.285091	1.274281	-2.037275
14	6	0	-0.517595	1.568402	0.311834
15	6	0	-1.722551	-0.905725	0.046248
16	1	0	-2.523208	-0.353867	0.572117
17	1	0	0.438187	2.079156	0.097034
18	6	0	-1.426327	-2.177009	0.864319
19	1	0	-1.095472	-1.937509	1.888274
20	1	0	-2.328456	-2.809062	0.948131
21	1	0	-0.640535	-2.792266	0.393883
22	6	0	-2.269373	-1.280124	-1.349901
23	1	0	-3.178719	-1.900756	-1.260125
24	1	0	-2.536215	-0.390533	-1.944449
25	1	0	-1.532381	-1.859623	-1.931678
26	6	0	-1.618516	2.316891	-0.470127
27	1	0	-1.662556	3.377471	-0.164914
28	1	0	-1.441302	2.295695	-1.558523
29	1	0	-2.615553	1.882198	-0.285867
30	6	0	-0.765383	1.683532	1.831217
31	1	0	-1.719026	1.212199	2.124120
32	1	0	0.037657	1.202153	2.413201
33	1	0	-0.813439	2.742452	2.142382

1a,b [1,3]-B

Sum of electronic and zero-point Energies= -456.044668
Sum of electronic and thermal Energies= -456.030994
Sum of electronic and thermal Enthalpies= -456.030049
Sum of electronic and thermal Free Energies= -456.082999

Standard orientation:

Center Number	Atomic Number	Atomic Type	Coordinates (Angstroms) X	Y	Z
1	5	0	-0.223346	0.053591	-0.000864
2	6	0	0.409465	-0.582542	1.819790
3	1	0	1.019801	-0.019417	2.533719
4	1	0	-0.569634	-0.901922	2.173665
5	6	0	1.029033	-1.103833	0.706420
6	6	0	2.447245	-1.030588	0.180075
7	6	0	0.481004	-1.326220	-0.646693
8	6	0	1.888159	-1.264732	-1.253905
9	1	0	2.994103	-1.916346	0.543086
10	1	0	3.048402	-0.137205	0.393440
11	1	0	-0.262820	-2.069078	-0.947746
12	1	0	2.232801	-2.204921	-1.710267
13	1	0	2.056223	-0.445255	-1.969130
14	6	0	0.191484	1.596438	-0.323828
15	6	0	-1.845704	-0.228430	0.121106
16	1	0	-2.231785	0.443969	0.909654
17	1	0	-0.131370	1.754025	-1.373714
18	6	0	-2.343080	-1.641007	0.474220
19	1	0	-1.896099	-2.050820	1.395212
20	1	0	-3.436406	-1.633253	0.626729
21	1	0	-2.145226	-2.365320	-0.334741
22	6	0	-2.513946	0.197651	-1.202869
23	1	0	-3.609453	0.061459	-1.156550
24	1	0	-2.326076	1.253891	-1.450874
25	1	0	-2.145313	-0.410172	-2.048110
26	6	0	-0.613249	2.591030	0.538834
27	1	0	-0.373096	3.635841	0.271325
28	1	0	-1.701912	2.470005	0.429498
29	1	0	-0.375321	2.466110	1.610735
30	6	0	1.669855	2.018337	-0.269177
31	1	0	2.093037	1.893719	0.742809
32	1	0	2.304543	1.461673	-0.973742
33	1	0	1.770738	3.088445	-0.524509

3

Sum of electronic and zero-point Energies= -610.462499
Sum of electronic and thermal Energies= -610.449236
Sum of electronic and thermal Enthalpies= -610.448292
Sum of electronic and thermal Free Energies= -610.502909

Standard orientation:

Center Number	Atomic Number	Atomic Type	Coordinates (Angstroms) X	Y	Z
1	8	0	-5.424461	0.728972	-0.000071
2	6	0	-4.702398	-0.249565	-0.000063
3	1	0	-5.127587	-1.282689	-0.000009
4	6	0	-3.225247	-0.187101	-0.000157
5	6	0	-2.527820	1.034492	-0.000194
6	6	0	-2.434304	-1.345742	-0.000182
7	7	0	-1.200578	1.096685	-0.000318
8	7	0	-1.103468	-1.305348	-0.000313
9	1	0	-2.900256	-2.338666	-0.000072
10	6	0	-0.536774	-0.081340	-0.000466
11	6	0	0.895568	-0.024861	-0.000973
12	6	0	2.115587	0.018387	-0.002392
13	6	0	3.586030	0.060837	-0.001502
14	6	0	4.114302	-0.861732	-1.125053
15	1	0	5.216081	-0.843197	-1.128126
16	1	0	3.783997	-1.900575	-0.971555
17	1	0	3.758965	-0.526389	-2.111630
18	6	0	4.058357	1.512421	-0.241678
19	1	0	3.687998	2.184084	0.548059
20	1	0	5.159708	1.548125	-0.240441
21	1	0	3.700120	1.888886	-1.212250
22	6	0	4.095680	-0.440568	1.370901
23	1	0	3.726284	0.198523	2.187634
24	1	0	3.767003	-1.473753	1.561506
25	1	0	5.197325	-0.417600	1.382544
26	1	0	-3.079512	1.980751	-0.000111

1a · 3

Sum of electronic and zero-point Energies= -1066.538737
Sum of electronic and thermal Energies= -1066.508626
Sum of electronic and thermal Enthalpies= -1066.507682
Sum of electronic and thermal Free Energies= -1066.602146

Standard orientation:

Center Number	Atomic Number	Atomic Type	Coordinates (Angstroms) X	Y	Z
1	6	0	2.932925	-0.166930	-0.223473
2	7	0	2.453904	0.291239	-1.400760
3	6	0	1.135385	0.289494	-1.550439
4	6	0	0.260794	-0.165747	-0.544003
5	6	0	0.870039	-0.624668	0.644493
6	7	0	2.185439	-0.626972	0.807053
7	1	0	0.741478	0.661374	-2.503496
8	1	0	0.259473	-0.994883	1.474491
9	6	0	4.351629	-0.164081	-0.048867
10	6	0	5.563455	-0.156942	0.103635
11	6	0	7.021860	-0.153527	0.287078
12	6	0	7.578814	-1.520084	-0.181380
13	1	0	7.356775	-1.694491	-1.245314
14	1	0	8.672342	-1.531462	-0.047151
15	1	0	7.147436	-2.345514	0.405303
16	6	0	7.638624	0.984021	-0.558746
17	1	0	7.249034	1.964908	-0.245635
18	1	0	8.732656	0.987510	-0.428472
19	1	0	7.415676	0.847079	-1.628014
20	6	0	7.343962	0.060375	1.783973
21	1	0	6.907908	-0.740682	2.400301
22	1	0	8.436079	0.056852	1.929456
23	1	0	6.949933	1.025073	2.138987
24	6	0	-1.174981	-0.151469	-0.744689
25	8	0	-1.959149	-0.588371	0.123618
26	1	0	-1.560084	0.223941	-1.703097
27	5	0	-3.581577	-0.545257	0.045186
28	6	0	-3.975361	0.817512	-0.823127
29	1	0	-3.858733	0.641903	-1.907986
30	1	0	-5.057389	0.966357	-0.652742
31	6	0	-3.245812	2.057617	-0.459205
32	6	0	-3.303438	2.958622	0.769129
33	6	0	-2.262637	2.801326	-1.034341
34	6	0	-2.179733	3.805934	0.093189
35	1	0	-3.021975	2.488348	1.726061
36	1	0	-4.261292	3.488463	0.911574
37	1	0	-1.743997	2.738562	-1.997987
38	1	0	-1.226627	3.840046	0.650943
39	1	0	-2.466377	4.842473	-0.159128
40	6	0	-3.955512	-0.485881	1.650786
41	6	0	-5.367779	0.027009	1.985021
42	6	0	-3.692641	-1.807526	2.392668
43	1	0	-3.254667	0.255221	2.084871
44	1	0	-6.155107	-0.648405	1.609098
45	1	0	-2.680597	-2.198371	2.190952
46	6	0	-3.994833	-1.935887	-0.755326
47	6	0	-5.515645	-2.022468	-0.975773
48	6	0	-3.274473	-2.164515	-2.092863
49	1	0	-6.077555	-1.968190	-0.030613
50	1	0	-2.179221	-2.243131	-1.978995
51	1	0	-5.880536	-1.205221	-1.622440
52	1	0	-3.715240	-2.783829	-0.101529

53	1	0	-5.563487	1.023826	1.557491
54	1	0	-5.514836	0.109476	3.077983
55	1	0	-3.784200	-1.687002	3.488129
56	1	0	-4.411497	-2.591841	2.096770
57	1	0	-3.602333	-3.107989	-2.566472
58	1	0	-3.479178	-1.358300	-2.820224
59	1	0	-5.796041	-2.971818	-1.468488

TS1(*RR*)

Sum of electronic and zero-point Energies= -1066.539011
Sum of electronic and thermal Energies= -1066.509779
Sum of electronic and thermal Enthalpies= -1066.508835
Sum of electronic and thermal Free Energies= -1066.600787

Standard orientation:

Center Number	Atomic Number	Atomic Type	Coordinates (Angstroms) X	Y	Z
1	6	0	2.872819	-0.023300	0.231665
2	7	0	2.363953	0.999532	0.950264
3	6	0	1.039920	1.084781	1.017322
4	6	0	0.186337	0.168582	0.373488
5	6	0	0.826967	-0.868597	-0.335613
6	7	0	2.148828	-0.967266	-0.409479
7	1	0	0.625946	1.909600	1.609198
8	1	0	0.237904	-1.634215	-0.850509
9	6	0	4.299426	-0.120277	0.144545
10	6	0	5.515269	-0.203130	0.069209
11	6	0	6.979723	-0.310368	-0.020636
12	6	0	7.374112	-0.603875	-1.486954
13	1	0	7.044529	0.207028	-2.154701
14	1	0	8.469885	-0.692091	-1.562668
15	1	0	6.924027	-1.545795	-1.836279
16	6	0	7.619263	1.017185	0.444982
17	1	0	7.345247	1.243498	1.486947
18	1	0	8.716782	0.941511	0.382739
19	1	0	7.293066	1.856230	-0.188844
20	6	0	7.448391	-1.470211	0.890525
21	1	0	7.000415	-2.425525	0.576743
22	1	0	8.544748	-1.562924	0.829901
23	1	0	7.173060	-1.284728	1.940152
24	6	0	-1.263035	0.285093	0.479884
25	8	0	-2.009659	-0.651666	0.065364
26	1	0	-1.663412	1.063569	1.143262
27	5	0	-3.573344	-0.582928	-0.025742
28	6	0	-1.683116	3.354343	-0.794937
29	6	0	-3.182186	3.036068	-0.493474
30	6	0	-1.616749	1.961758	-1.383693
31	6	0	-2.949416	1.690168	-1.163526
32	1	0	-3.907900	3.682207	-1.015541
33	1	0	-3.456437	3.001428	0.574310
34	1	0	-0.845457	1.447931	-1.964667
35	6	0	-4.171054	0.032394	1.379421
36	6	0	-5.639128	0.537317	1.300816
37	6	0	-3.989257	-0.942551	2.559222
38	1	0	-3.563521	0.922721	1.631057
39	1	0	-6.119411	0.338104	0.329762
40	1	0	-2.942153	-1.278244	2.659247
41	6	0	-4.025606	-2.110765	-0.423635
42	6	0	-5.543595	-2.296626	-0.585376
43	6	0	-3.304742	-2.682767	-1.656153
44	1	0	-6.098284	-2.049300	0.333474
45	1	0	-2.210209	-2.580030	-1.578605
46	1	0	-5.948315	-1.662319	-1.394654
47	1	0	-3.719324	-2.742701	0.434822
48	1	0	-5.710598	1.625609	1.468036
49	1	0	-1.522930	4.177197	-1.512579
50	1	0	-1.051685	3.548579	0.090006
51	6	0	-3.767645	0.504599	-1.343964
52	1	0	-3.478643	-0.041226	-2.254393

53	1	0	-4.842914	0.738734	-1.377811
54	1	0	-4.277789	-0.480186	3.521187
55	1	0	-4.609075	-1.846994	2.436958
56	1	0	-6.273380	0.060621	2.068131
57	1	0	-3.526085	-3.757335	-1.792075
58	1	0	-3.618288	-2.175865	-2.586412
59	1	0	-5.795470	-3.341903	-0.844592

TS2(*SR*)

Sum of electronic and zero-point Energies= -1066.535822
Sum of electronic and thermal Energies= -1066.506897
Sum of electronic and thermal Enthalpies= -1066.505953
Sum of electronic and thermal Free Energies= -1066.597334

Standard orientation:

Center Number	Atomic Number	Atomic Type	Coordinates (Angstroms) X	Y	Z
1	6	0	-2.738962	-0.255089	-0.228781
2	7	0	-2.280431	-0.298066	-1.497882
3	6	0	-0.961534	-0.286934	-1.659816
4	6	0	-0.060785	-0.236754	-0.579361
5	6	0	-0.650828	-0.205503	0.698600
6	7	0	-1.969812	-0.212462	0.877725
7	1	0	-0.590770	-0.323086	-2.691198
8	1	0	-0.025227	-0.188651	1.596257
9	6	0	-4.161491	-0.257945	-0.043236
10	6	0	-5.371984	-0.259861	0.114360
11	6	0	-6.830465	-0.264171	0.311377
12	6	0	-7.227431	0.977512	1.144001
13	1	0	-6.950399	1.907341	0.623609
14	1	0	-8.317787	0.980697	1.303721
15	1	0	-6.731929	0.969016	2.127003
16	6	0	-7.533478	-0.224637	-1.064196
17	1	0	-7.259798	-1.100315	-1.672816
18	1	0	-8.626300	-0.227822	-0.922577
19	1	0	-7.258608	0.683810	-1.622192
20	6	0	-7.227742	-1.552197	1.070693
21	1	0	-6.732941	-1.600456	2.052887
22	1	0	-8.318243	-1.564217	1.229054
23	1	0	-6.950349	-2.450407	0.497678
24	6	0	1.388870	-0.230998	-0.788657
25	8	0	2.150581	-0.437622	0.238231
26	1	0	1.740961	-0.543332	-1.779143
27	5	0	3.666708	-0.355295	0.188157
28	6	0	3.981875	1.379489	-0.265108
29	1	0	4.540496	1.832980	0.563564
30	1	0	4.604917	1.263703	-1.161744
31	6	0	2.729483	2.003327	-0.518704
32	6	0	1.798465	2.812830	0.360559
33	6	0	1.749691	1.853202	-1.507045
34	6	0	0.788172	2.812608	-0.826560
35	1	0	2.191493	3.795662	0.669914
36	1	0	1.453744	2.276591	1.261212
37	1	0	1.828305	1.556541	-2.557650
38	1	0	0.697996	3.788494	-1.331884
39	1	0	-0.226160	2.442751	-0.605205
40	6	0	4.215871	-1.272367	-1.068319
41	6	0	5.721911	-1.211373	-1.371962
42	6	0	3.781065	-2.743310	-0.919255
43	1	0	3.717263	-0.881676	-1.979960
44	1	0	6.313202	-1.750101	-0.614098
45	1	0	2.692528	-2.845635	-0.774705
46	6	0	4.196266	-0.655063	1.708475
47	6	0	5.699849	-0.428671	1.940649
48	1	0	6.329338	-1.074976	1.311589
49	1	0	5.993981	0.615979	1.733193
50	1	0	4.007978	-1.735177	1.880661
51	1	0	6.107433	-0.178466	-1.413976
52	1	0	5.949883	-1.682126	-2.345584

53	1	0	4.052958	-3.341730	-1.808240
54	1	0	4.269090	-3.216175	-0.049635
55	1	0	5.974864	-0.632395	2.991835
56	6	0	3.402709	0.104254	2.785892
57	1	0	3.552636	1.196112	2.703199
58	1	0	2.320968	-0.087473	2.714856
59	1	0	3.724850	-0.184410	3.803214

5a(*RR*)

Sum of electronic and zero-point Energies= -1066.589277
Sum of electronic and thermal Energies= -1066.559196
Sum of electronic and thermal Enthalpies= -1066.558252
Sum of electronic and thermal Free Energies= -1066.654125

Standard orientation:

Center Number	Atomic Number	Atomic Type	Coordinates (Angstroms) X	Y	Z
1	6	0	2.553922	0.208506	0.057184
2	7	0	1.998985	0.610304	1.214737
3	6	0	0.675160	0.781780	1.217098
4	6	0	-0.124889	0.569187	0.087502
5	6	0	0.562522	0.138582	-1.058838
6	7	0	1.882742	-0.042485	-1.084426
7	1	0	0.225123	1.099450	2.165435
8	1	0	0.020949	-0.074989	-1.986646
9	6	0	3.980670	0.027405	0.034981
10	6	0	5.190863	-0.126986	0.014142
11	6	0	6.651841	-0.310071	-0.010956
12	6	0	7.324697	1.068487	-0.211364
13	1	0	7.067636	1.756668	0.608671
14	1	0	8.419643	0.944810	-0.231259
15	1	0	7.009933	1.527561	-1.161268
16	6	0	7.108350	-0.925156	1.331955
17	1	0	6.636251	-1.905939	1.497658
18	1	0	8.201689	-1.063537	1.323811
19	1	0	6.847275	-0.268536	2.176242
20	6	0	7.027215	-1.251703	-1.177689
21	1	0	6.706494	-0.831101	-2.143289
22	1	0	8.119965	-1.392093	-1.205139
23	1	0	6.553884	-2.238375	-1.056488
24	6	0	-1.621355	0.796742	0.099588
25	8	0	-2.260278	-0.330483	-0.499304
26	1	0	-1.948271	0.915951	1.144834
27	5	0	-3.107160	-1.230037	0.101113
28	6	0	-4.616312	2.128399	-1.059129
29	1	0	-4.663206	1.305257	-1.777750
30	1	0	-5.553650	2.645251	-0.826637
31	6	0	-3.461651	2.506313	-0.502402
32	6	0	-3.056639	3.647350	0.413918
33	6	0	-2.014497	2.063213	-0.689463
34	6	0	-1.582615	3.399003	-0.009876
35	1	0	-3.239105	3.430706	1.480529
36	1	0	-3.486816	4.635724	0.187276
37	1	0	-1.696654	1.929243	-1.736447
38	1	0	-0.844599	3.318460	0.802537
39	1	0	-1.219839	4.134031	-0.741976
40	6	0	-3.567131	-1.143686	1.627622
41	6	0	-5.100095	-1.004034	1.725847
42	6	0	-3.082119	-2.383012	2.408813
43	1	0	-3.129165	-0.257160	2.120485
44	1	0	-5.615878	-1.862834	1.263662
45	1	0	-3.493363	-3.315361	1.986150
46	6	0	-3.577309	-2.421325	-0.858716
47	6	0	-4.281415	-1.907998	-2.128293
48	6	0	-2.375953	-3.315981	-1.228859
49	1	0	-5.189387	-1.330048	-1.887684
50	1	0	-1.875886	-3.727932	-0.336043
51	1	0	-3.615371	-1.255351	-2.716910
52	1	0	-4.299575	-3.052307	-0.308773

53	1	0	-5.459402	-0.090114	1.224430
54	1	0	-5.424786	-0.955851	2.780426
55	1	0	-3.403322	-2.333465	3.464333
56	1	0	-1.982713	-2.470576	2.401579
57	1	0	-4.587661	-2.745149	-2.780896
58	1	0	-2.693952	-4.170793	-1.852173
59	1	0	-1.619860	-2.752366	-1.800375

5a(*SR*)

Sum of electronic and zero-point Energies= -1066.587505
Sum of electronic and thermal Energies= -1066.557294
Sum of electronic and thermal Enthalpies= -1066.556350
Sum of electronic and thermal Free Energies= -1066.654333

Standard orientation:

Center Number	Atomic Number	Atomic Type	Coordinates (Angstroms) X	Y	Z
1	6	0	-2.381616	-0.077242	-0.259901
2	7	0	-1.898216	0.103065	-1.502604
3	6	0	-0.584516	0.313262	-1.608487
4	6	0	0.275464	0.358754	-0.503223
5	6	0	-0.336898	0.127976	0.739931
6	7	0	-1.647420	-0.085335	0.869206
7	1	0	-0.191876	0.442545	-2.624368
8	1	0	0.259773	0.105888	1.655970
9	6	0	-3.798338	-0.289122	-0.126276
10	6	0	-4.999676	-0.468686	-0.010790
11	6	0	-6.449790	-0.683368	0.130446
12	6	0	-7.135362	0.681393	0.376841
13	1	0	-6.960249	1.369320	-0.464751
14	1	0	-8.222223	0.533960	0.484907
15	1	0	-6.755930	1.154004	1.295962
16	6	0	-6.998508	-1.318955	-1.167322
17	1	0	-6.519082	-2.290350	-1.364385
18	1	0	-8.084377	-1.481219	-1.071462
19	1	0	-6.820241	-0.663576	-2.033866
20	6	0	-6.708823	-1.623870	1.329599
21	1	0	-6.321300	-1.188525	2.263623
22	1	0	-7.792472	-1.788064	1.445468
23	1	0	-6.224630	-2.600840	1.176793
24	6	0	1.755931	0.631651	-0.650829
25	8	0	2.460686	-0.197784	0.275499
26	1	0	2.045974	0.384940	-1.685089
27	5	0	3.553321	-0.984260	0.001774
28	6	0	1.888476	2.715631	2.136588
29	1	0	1.393165	3.367443	2.864340
30	1	0	2.621850	2.001621	2.522716
31	6	0	1.609115	2.798418	0.832537
32	6	0	0.714491	3.704503	0.004565
33	6	0	2.161667	2.104300	-0.409790
34	6	0	1.420536	3.171147	-1.272150
35	1	0	0.798005	4.784988	0.203753
36	1	0	-0.352156	3.427176	0.059712
37	1	0	3.260672	2.135740	-0.492677
38	1	0	2.114197	3.896506	-1.720656
39	1	0	0.760089	2.784688	-2.062720
40	6	0	4.197898	-1.105214	-1.455331
41	6	0	5.736718	-1.069697	-1.447743
42	6	0	3.687259	-2.415802	-2.098045
43	1	0	3.858471	-0.274365	-2.100189
44	1	0	6.161913	-1.879510	-0.831497
45	1	0	2.587607	-2.435304	-2.181535
46	6	0	4.109402	-1.809997	1.252589
47	6	0	4.987691	-0.900687	2.140083
48	6	0	2.995272	-2.466563	2.085249
49	1	0	5.822206	-0.449565	1.578026
50	1	0	2.382853	-3.155086	1.478864
51	1	0	4.396674	-0.077064	2.575160
52	1	0	4.763645	-2.615211	0.869153

53	1	0	6.123258	-0.114240	-1.056044
54	1	0	3.414323	-3.048624	2.925318
55	1	0	2.316058	-1.710256	2.511448
56	1	0	5.425636	-1.470210	2.979094
57	1	0	4.100247	-2.543639	-3.114315
58	1	0	3.989242	-3.297897	-1.507738
59	1	0	6.137681	-1.192814	-2.469343

1b · 3

Sum of electronic and zero-point Energies= -1066.528227
Sum of electronic and thermal Energies= -1066.498332
Sum of electronic and thermal Enthalpies= -1066.497388
Sum of electronic and thermal Free Energies= -1066.590888

Standard orientation:

Center Number	Atomic Number	Atomic Type	Coordinates (Angstroms) X	Y	Z
1	5	0	-3.423121	0.543765	0.172129
2	6	0	-1.811834	-2.146544	-1.566830
3	1	0	-1.599108	-3.211073	-1.717325
4	1	0	-0.962118	-1.462035	-1.639853
5	6	0	-3.072647	-1.708425	-1.392748
6	6	0	-4.413124	-2.423296	-1.437734
7	6	0	-3.710887	-0.350548	-1.219349
8	6	0	-5.110989	-1.036926	-1.363838
9	1	0	-4.585232	-2.984064	-2.371860
10	1	0	-4.615319	-3.103721	-0.594961
11	1	0	-3.464757	0.322628	-2.059829
12	1	0	-5.648382	-0.764880	-2.285243
13	1	0	-5.798410	-0.906082	-0.515421
14	6	0	-3.891562	-0.052635	1.644573
15	6	0	-5.219228	0.511658	2.187625
16	6	0	-3.927384	-1.583192	1.836955
17	1	0	-3.054981	-2.110051	1.417435
18	6	0	-3.798696	2.134824	-0.072149
19	6	0	-5.207360	2.320864	-0.666213
20	6	0	-2.790468	2.904041	-0.943124
21	1	0	-5.988945	1.802010	-0.090289
22	1	0	-2.647513	2.421718	-1.927578
23	1	0	-4.817792	-2.018511	1.355306
24	1	0	-5.256119	1.938606	-1.700876
25	1	0	-3.797181	2.630903	0.919940
26	8	0	-1.807276	0.541637	0.155775
27	6	0	-1.027632	-0.216552	0.769900
28	1	0	-1.413461	-0.934983	1.507767
29	6	0	0.408054	-0.143673	0.582705
30	6	0	1.271253	-0.973630	1.325595
31	6	0	1.030754	0.742477	-0.323727
32	7	0	2.590738	-0.932100	1.190363
33	1	0	0.866356	-1.690390	2.049738
34	7	0	2.347364	0.792738	-0.468993
35	1	0	0.430314	1.419378	-0.940121
36	6	0	3.083021	-0.048302	0.295026
37	6	0	4.503157	0.003702	0.139310
38	6	0	5.716425	0.046569	0.005373
39	6	0	7.175540	0.100935	-0.164728
40	6	0	7.525412	-0.400461	-1.586691
41	1	0	8.617257	-0.360084	-1.728985
42	1	0	7.050271	0.227646	-2.355663
43	1	0	7.194295	-1.440165	-1.732201
44	6	0	7.644080	1.565600	0.004547
45	1	0	7.398436	1.945864	1.008016
46	1	0	7.170247	2.221221	-0.741932
47	1	0	8.736543	1.617394	-0.128820
48	6	0	7.850510	-0.798737	0.894919
49	1	0	7.523361	-1.844746	0.791173
50	1	0	7.607911	-0.459191	1.913669
51	1	0	8.944311	-0.762342	0.767882
52	1	0	-3.112598	0.337338	2.337913

53	1	0	-6.082345	0.176221	1.587133
54	1	0	-5.399095	0.164250	3.222003
55	1	0	-5.235024	1.612300	2.207767
56	1	0	-3.989830	-1.848911	2.908411
57	1	0	-5.487097	3.389908	-0.708233
58	1	0	-3.136007	3.935815	-1.139590
59	1	0	-1.799248	2.975125	-0.468201

TS3

Sum of electronic and zero-point Energies= -1066.531450
Sum of electronic and thermal Energies= -1066.502657
Sum of electronic and thermal Enthalpies= -1066.501713
Sum of electronic and thermal Free Energies= -1066.591440

Standard orientation:

Center Number	Atomic Number	Atomic Type	Coordinates (Angstroms) X	Y	Z
1	5	0	3.494608	0.557465	0.087003
2	6	0	1.570343	-1.676917	-1.600812
3	1	0	0.937066	-2.570220	-1.618263
4	1	0	1.201509	-0.802343	-2.142371
5	6	0	2.880216	-1.756095	-1.209300
6	6	0	3.735724	-2.880273	-0.669194
7	6	0	3.924009	-0.740461	-1.019672
8	6	0	4.914222	-1.866820	-0.599838
9	1	0	3.867082	-3.713322	-1.380066
10	1	0	3.402024	-3.309841	0.290826
11	1	0	4.189433	-0.163975	-1.920051
12	1	0	5.694578	-2.068548	-1.349501
13	1	0	5.396103	-1.759177	0.382263
14	6	0	4.131437	0.356978	1.598628
15	6	0	4.012851	1.626472	2.465621
16	6	0	3.879818	1.997926	-0.605603
17	6	0	5.396513	2.175027	-0.787489
18	6	0	3.160209	2.278199	-1.935204
19	1	0	3.444672	1.554585	-2.721818
20	1	0	2.956434	1.910660	2.619776
21	1	0	5.811403	1.417384	-1.477414
22	1	0	3.543072	2.786663	0.095753
23	1	0	5.213343	0.185831	1.432343
24	8	0	1.945544	0.531345	0.109977
25	6	0	1.206125	-0.484918	0.341256
26	1	0	1.580498	-1.317788	0.945517
27	6	0	-0.251311	-0.341913	0.257430
28	6	0	-1.109149	-1.356489	0.719027
29	6	0	-0.884741	0.811045	-0.244178
30	7	0	-2.433071	-1.253659	0.671916
31	1	0	-0.698731	-2.281866	1.140639
32	7	0	-2.207948	0.928520	-0.299198
33	1	0	-0.291470	1.658700	-0.601261
34	6	0	-2.936449	-0.113101	0.154636
35	6	0	-4.363752	0.002523	0.084694
36	6	0	-5.579199	0.098422	0.022562
37	6	0	-7.043753	0.220469	-0.050523
38	6	0	-7.433820	0.781836	-1.437701
39	1	0	-6.983225	1.772366	-1.604008
40	1	0	-7.101215	0.109656	-2.243740
41	1	0	-8.529403	0.882612	-1.499704
42	6	0	-7.517034	1.189596	1.059138
43	1	0	-8.613328	1.291636	1.013539
44	1	0	-7.244536	0.811935	2.056675
45	1	0	-7.069083	2.186899	0.930505
46	6	0	-7.683770	-1.170835	0.156810
47	1	0	-7.413254	-1.587441	1.139318
48	1	0	-8.781167	-1.085471	0.105532
49	1	0	-7.354209	-1.876879	-0.621050
50	6	0	3.622373	-0.832097	2.433660
51	1	0	3.652554	-1.790739	1.892555
52	1	0	4.227241	-0.961207	3.349836

53	1	0	2.583946	-0.672078	2.774656
54	1	0	4.453897	1.468807	3.467546
55	1	0	4.523806	2.493715	2.019872
56	1	0	5.946566	2.080924	0.163411
57	1	0	5.646761	3.164039	-1.213956
58	1	0	3.407531	3.281502	-2.328511
59	1	0	2.064684	2.229434	-1.826399

5b

Sum of electronic and zero-point Energies= -1066.586451
Sum of electronic and thermal Energies= -1066.556281
Sum of electronic and thermal Enthalpies= -1066.555337
Sum of electronic and thermal Free Energies= -1066.653392

Standard orientation:

Center Number	Atomic Number	Atomic Type	Coordinates (Angstroms) X	Y	Z
1	5	0	-2.793653	-1.506496	0.090326
2	6	0	-1.921561	1.954840	-0.596842
3	1	0	-1.319982	2.786346	-0.191332
4	1	0	-1.635718	1.823710	-1.652978
5	6	0	-3.374102	2.294168	-0.497493
6	6	0	-4.199226	2.902514	0.627146
7	6	0	-4.424792	2.221774	-1.342278
8	6	0	-5.410160	2.810159	-0.356594
9	1	0	-3.914414	3.929103	0.916625
10	1	0	-4.298256	2.296191	1.543231
11	1	0	-4.530107	1.875043	-2.375374
12	1	0	-5.852442	3.781615	-0.638244
13	1	0	-6.224124	2.140025	-0.029444
14	6	0	-2.933340	-1.798387	1.662441
15	6	0	-1.567414	-2.041277	2.337880
16	1	0	-0.974476	-2.802251	1.803218
17	6	0	-3.429053	-2.531818	-0.958140
18	6	0	-4.971267	-2.481112	-0.861654
19	1	0	-5.330466	-2.663439	0.164755
20	1	0	-0.957789	-1.123882	2.385030
21	1	0	-5.358913	-1.498219	-1.181214
22	1	0	-3.127231	-3.543734	-0.617425
23	1	0	-3.481520	-2.757202	1.723757
24	8	0	-2.146084	-0.436036	-0.470482
25	6	0	-1.509857	0.669564	0.163223
26	1	0	-1.827823	0.753782	1.214010
27	6	0	-0.006332	0.492326	0.123917
28	6	0	0.826915	1.024487	1.117742
29	6	0	0.655990	-0.170376	-0.919559
30	7	0	2.156149	0.924473	1.082392
31	1	0	0.399043	1.549494	1.980659
32	7	0	1.984532	-0.282714	-0.975611
33	1	0	0.087661	-0.633047	-1.732755
34	6	0	2.686341	0.275968	0.028160
35	6	0	4.119835	0.169612	-0.028610
36	6	0	5.335908	0.083661	-0.079772
37	6	0	6.803811	-0.020206	-0.137794
38	6	0	7.233182	-0.382118	-1.577667
39	1	0	6.798660	-1.344670	-1.888895
40	1	0	6.908926	0.390219	-2.292248
41	1	0	8.331071	-0.463424	-1.628955
42	6	0	7.266388	-1.124528	0.841732
43	1	0	8.364623	-1.210816	0.808366
44	1	0	6.967797	-0.887138	1.874495
45	1	0	6.833193	-2.099787	0.570884
46	6	0	7.422646	1.335920	0.272626
47	1	0	7.124820	1.612447	1.295840
48	1	0	8.522058	1.268032	0.236677
49	1	0	7.102522	2.138587	-0.409694
50	1	0	-1.700662	-2.396846	3.375026
51	6	0	-3.773000	-0.776192	2.454818
52	1	0	-4.754012	-0.595806	1.985322

53	1	0	-3.959307	-1.138448	3.481340
54	1	0	-3.266226	0.198347	2.547767
55	6	0	-2.965351	-2.356328	-2.409442
56	1	0	-3.228732	-1.357531	-2.795596
57	1	0	-1.872527	-2.466198	-2.504103
58	1	0	-3.432340	-3.104159	-3.074773
59	1	0	-5.434520	-3.243784	-1.512259

2a

Sum of electronic and zero-point Energies= -1000.923490
Sum of electronic and thermal Energies= -1000.895748
Sum of electronic and thermal Enthalpies= -1000.894804
Sum of electronic and thermal Free Energies= -1000.981272

Standard orientation:

Center Number	Atomic Number	Atomic Type	Coordinates (Angstroms) X	Y	Z
1	5	0	0.042147	0.686333	0.494108
2	6	0	-2.256097	4.501651	-0.536298
3	6	0	-0.925907	3.871244	-1.059901
4	6	0	-1.980947	3.738681	0.742015
5	6	0	-0.830887	3.183904	0.294573
6	1	0	-0.121733	4.596663	-1.275865
7	1	0	-1.035175	3.204468	-1.932933
8	1	0	-2.522365	3.674064	1.691936
9	6	0	-1.419780	0.062168	0.452699
10	6	0	-1.853351	0.110330	-1.065726
11	6	0	-1.612666	-1.337789	1.123645
12	1	0	-2.096976	0.758283	0.974053
13	6	0	-2.613606	-1.144127	-1.517494
14	1	0	-0.981705	0.217561	-1.737978
15	6	0	-2.472582	-2.281534	0.258356
16	1	0	-0.629263	-1.838820	1.188784
17	6	0	-1.790432	-2.393612	-1.128312
18	6	0	-3.686440	-1.598069	-0.472489
19	1	0	-2.688326	-3.216180	0.805868
20	1	0	-2.074330	-3.294622	-1.689026
21	1	0	-2.941439	-1.038432	-2.566614
22	6	0	-4.548602	-0.537947	0.218230
23	1	0	-5.255454	-0.100669	-0.508715
24	1	0	-5.151729	-0.989921	1.024105
25	1	0	-3.969980	0.286740	0.653306
26	6	0	-4.637356	-2.655291	-1.058125
27	1	0	-5.235789	-3.119288	-0.255358
28	1	0	-5.340159	-2.189480	-1.770204
29	1	0	-4.111760	-3.462875	-1.589131
30	6	0	1.357376	-0.167416	0.201733
31	6	0	1.889998	0.201731	-1.231877
32	6	0	2.455203	-0.052532	1.320712
33	6	0	3.422106	0.265790	-1.321502
34	1	0	1.496889	-0.535721	-1.951560
35	6	0	3.878366	0.010053	0.724880
36	1	0	2.332397	0.911701	1.847659
37	6	0	3.937444	1.229453	-0.228002
38	6	0	4.119430	-0.896800	-0.538015
39	1	0	4.631118	-0.053506	1.530596
40	1	0	3.741723	0.437044	-2.364416
41	1	0	-0.691854	-2.295346	-1.149163
42	1	0	1.503913	1.183528	-1.563846
43	1	0	1.080205	-1.232308	0.160714
44	6	0	2.293000	-1.149047	2.380684
45	1	0	1.293239	-1.109370	2.844641
46	1	0	2.421967	-2.157063	1.955563
47	1	0	3.036128	-1.026902	3.186752
48	6	0	-2.121474	-1.198476	2.564138
49	1	0	-1.445377	-0.563874	3.162311
50	1	0	-3.123556	-0.743214	2.607596
51	1	0	-2.177552	-2.182491	3.059834
52	1	0	-2.468063	1.010728	-1.224767

53	6	0	5.619468	-0.982925	-0.868964
54	1	0	6.131757	-1.648681	-0.153356
55	1	0	5.766897	-1.403443	-1.878572
56	1	0	6.127482	-0.007834	-0.837074
57	6	0	3.554575	-2.317122	-0.634883
58	1	0	4.065996	-2.988275	0.076082
59	1	0	2.477005	-2.381122	-0.439750
60	1	0	3.733546	-2.721054	-1.646800
61	1	0	3.294327	2.088688	0.025856
62	1	0	4.956270	1.596142	-0.412704
63	1	0	-2.249260	5.603406	-0.457722
64	1	0	-3.159126	4.201941	-1.097406
65	6	0	0.195936	2.244656	0.831146
66	1	0	0.126706	2.237721	1.941004
67	1	0	1.206017	2.618296	0.588416

2b(*R*)

Sum of electronic and zero-point Energies= -1000.917318
Sum of electronic and thermal Energies= -1000.889563
Sum of electronic and thermal Enthalpies= -1000.888619
Sum of electronic and thermal Free Energies= -1000.974656

Standard orientation:

Center Number	Atomic Number	Atomic Type	Coordinates (Angstroms) X	Y	Z
1	5	0	0.098128	0.521644	0.096165
2	6	0	-0.622759	3.638086	-1.603408
3	1	0	-1.267037	4.476438	-1.890047
4	1	0	0.022697	3.218321	-2.382353
5	6	0	-0.621335	3.157211	-0.354860
6	6	0	-1.339248	3.574153	0.917309
7	6	0	0.208483	2.093420	0.356923
8	6	0	-0.472052	2.559124	1.708940
9	1	0	-2.420129	3.353889	0.925347
10	1	0	-1.209762	4.632152	1.199683
11	1	0	-1.042844	1.786706	2.244682
12	1	0	0.235079	3.017356	2.415444
13	6	0	1.426600	-0.354108	0.219894
14	6	0	2.505841	0.030163	1.281013
15	6	0	1.953791	-0.320254	-1.268911
16	6	0	3.929834	0.026707	0.685239
17	6	0	3.480913	-0.195263	-1.368925
18	1	0	1.604137	-1.225963	-1.790321
19	6	0	4.220109	-1.124007	-0.348186
20	6	0	3.939210	1.008022	-0.513612
21	1	0	3.803581	-0.241840	-2.423803
22	1	0	4.680994	0.176157	1.480930
23	6	0	-1.222248	-0.304219	-0.255661
24	6	0	-1.586866	-1.104822	1.057893
25	6	0	-2.447009	0.396505	-0.922454
26	6	0	-3.097655	-1.294319	1.251125
27	1	0	-1.079537	-2.082799	1.034044
28	6	0	-3.788542	-0.134097	-0.373847
29	1	0	-2.434324	1.466037	-0.654091
30	6	0	-3.785071	0.087350	1.159414
31	6	0	-3.831263	-1.678662	-0.077127
32	1	0	-4.632261	0.279904	-0.953944
33	1	0	-3.296681	-1.920554	2.138677
34	1	0	1.522595	0.532560	-1.831441
35	1	0	-1.207380	-0.592306	1.961468
36	1	0	-0.885170	-1.075603	-0.971063
37	6	0	-2.355134	0.340017	-2.451681
38	1	0	-1.413089	0.791064	-2.805596
39	1	0	-2.391601	-0.694453	-2.830170
40	1	0	-3.184052	0.898559	-2.918522
41	6	0	-3.191078	-2.702017	-1.019816
42	1	0	-3.748042	-2.757570	-1.970675
43	1	0	-2.141539	-2.489251	-1.258823
44	1	0	-3.230751	-3.706367	-0.562561
45	6	0	-5.273348	-2.144833	0.187292
46	1	0	-5.276283	-3.154387	0.633181
47	1	0	-5.826570	-1.479306	0.866402
48	1	0	-5.838010	-2.197828	-0.759361
49	1	0	-3.215150	0.954235	1.533005
50	1	0	-4.788137	0.106162	1.606875
51	1	0	3.254404	1.870516	-0.455160
52	1	0	4.941162	1.372233	-0.778180

53	6	0	3.710219	-2.551519	-0.131317
54	1	0	4.237929	-3.030405	0.711016
55	1	0	2.633309	-2.610348	0.070523
56	1	0	3.914313	-3.160764	-1.029352
57	6	0	5.724614	-1.217509	-0.653906
58	1	0	6.257754	-1.689472	0.189174
59	1	0	5.897990	-1.840907	-1.547911
60	1	0	6.191663	-0.238089	-0.835126
61	1	0	1.146044	-1.400794	0.417042
62	1	0	2.338764	1.075987	1.598789
63	6	0	2.376827	-0.828784	2.545566
64	1	0	1.367097	-0.740733	2.980917
65	1	0	2.557851	-1.896153	2.341710
66	1	0	3.098582	-0.506786	3.315253
67	1	0	1.274765	2.378110	0.342142

2b(*S*)

Sum of electronic and zero-point Energies= -1000.914987
Sum of electronic and thermal Energies= -1000.887586
Sum of electronic and thermal Enthalpies= -1000.886642
Sum of electronic and thermal Free Energies= -1000.970345

Standard orientation:

Center Number	Atomic Number	Atomic Type	Coordinates (Angstroms) X	Y	Z
1	5	0	-0.039458	0.457698	0.196701
2	6	0	-0.130672	3.238794	-1.704681
3	1	0	0.105734	4.182746	-2.207882
4	1	0	-0.449754	2.406362	-2.337160
5	6	0	-0.029778	3.132846	-0.375143
6	6	0	0.491324	4.077118	0.689040
7	6	0	-0.165729	1.988464	0.644667
8	6	0	0.800573	2.826555	1.549197
9	1	0	-0.323363	4.681590	1.124906
10	1	0	1.321382	4.748890	0.416337
11	1	0	0.557448	2.873359	2.621883
12	1	0	1.850252	2.525552	1.434089
13	6	0	1.274827	-0.445759	0.197384
14	6	0	2.342612	-0.348240	1.334758
15	6	0	1.859133	-0.216496	-1.254534
16	6	0	3.780603	-0.395454	0.776574
17	6	0	3.393994	-0.237288	-1.296037
18	1	0	1.444511	-0.983646	-1.928755
19	6	0	4.003388	-1.386458	-0.425713
20	6	0	3.928742	0.761312	-0.244522
21	1	0	3.751999	-0.144770	-2.336485
22	1	0	4.512131	-0.451970	1.602161
23	6	0	-1.356172	-0.298825	-0.290138
24	6	0	-1.790801	-0.938671	1.091077
25	6	0	-2.505561	0.459641	-1.024524
26	6	0	-3.307346	-0.904138	1.326178
27	1	0	-1.414465	-1.972714	1.141038
28	6	0	-3.887278	0.162849	-0.403932
29	1	0	-2.360943	1.546549	-0.888711
30	6	0	-3.820720	0.535918	1.097781
31	6	0	-4.109409	-1.327468	0.050824
32	1	0	-4.688317	0.616852	-1.013591
33	1	0	-3.560715	-1.406966	2.275966
34	1	0	1.539962	0.757386	-1.669992
35	1	0	-1.319844	-0.410420	1.946128
36	1	0	-1.060733	-1.142503	-0.934756
37	6	0	-2.464966	0.200105	-2.536498
38	1	0	-1.474151	0.442903	-2.955781
39	1	0	-2.673098	-0.853818	-2.779568
40	1	0	-3.210133	0.819849	-3.063254
41	6	0	-3.608835	-2.510759	-0.782377
42	1	0	-4.188949	-2.604385	-1.716146
43	1	0	-2.547284	-2.443909	-1.052329
44	1	0	-3.750281	-3.449408	-0.218169
45	6	0	-5.591075	-1.586718	0.371363
46	1	0	-5.706485	-2.537877	0.919038
47	1	0	-6.047466	-0.793331	0.981678
48	1	0	-6.175654	-1.666631	-0.561156
49	1	0	-3.143992	1.361433	1.374802
50	1	0	-4.803926	0.721878	1.550939
51	1	0	3.324898	1.665885	-0.065753
52	1	0	4.970336	1.062786	-0.420479

53	6	0	3.360312	-2.776084	-0.462634
54	1	0	3.805890	-3.432908	0.303851
55	1	0	2.274400	-2.766661	-0.305831
56	1	0	3.550215	-3.247912	-1.442783
57	6	0	5.504463	-1.573734	-0.705942
58	1	0	5.958282	-2.225643	0.060170
59	1	0	5.655820	-2.057416	-1.686319
60	1	0	6.065858	-0.627756	-0.711328
61	1	0	0.927920	-1.492353	0.204024
62	1	0	2.260994	0.634258	1.826925
63	6	0	2.086157	-1.394140	2.427657
64	1	0	1.076504	-1.277348	2.856340
65	1	0	2.165896	-2.423782	2.043764
66	1	0	2.810393	-1.286459	3.252955
67	1	0	-1.180701	2.103599	1.076779

2a,b [1,3]-B

Sum of electronic and zero-point Energies= -1000.897168
Sum of electronic and thermal Energies= -1000.870946
Sum of electronic and thermal Enthalpies= -1000.870002
Sum of electronic and thermal Free Energies= -1000.949644

Standard orientation:

Center Number	Atomic Number	Atomic Type	Coordinates (Angstroms) X	Y	Z
1	5	0	-0.016834	0.957023	0.060918
2	6	0	-0.435644	1.941747	1.737920
3	1	0	0.092236	1.849291	2.691429
4	1	0	-1.505554	1.733951	1.753191
5	6	0	0.146024	2.662777	0.717207
6	6	0	1.473924	3.363136	0.511293
7	6	0	-0.053316	2.478160	-0.719568
8	6	0	1.256424	3.217700	-1.022249
9	1	0	2.376638	2.930592	0.963494
10	1	0	1.382176	4.409361	0.845353
11	1	0	2.023703	2.639120	-1.556333
12	1	0	1.121887	4.182041	-1.535167
13	6	0	-1.425351	0.133190	-0.229489
14	6	0	-2.708072	0.903394	-0.697832
15	6	0	-1.718391	-0.932412	0.888509
16	6	0	-4.006708	0.275665	-0.144042
17	6	0	-3.209711	-1.168278	1.168453
18	1	0	-1.248291	-1.891518	0.612303
19	6	0	-4.039603	-1.299092	-0.149662
20	6	0	-3.898256	0.196229	1.397027
21	1	0	-3.343174	-1.956715	1.930458
22	1	0	-4.886461	0.786806	-0.573998
23	6	0	1.265781	-0.063352	0.085225
24	6	0	1.837039	-0.277666	-1.361889
25	6	0	2.375166	0.051882	1.188661
26	6	0	3.338134	-0.600492	-1.398635
27	1	0	1.271491	-1.089325	-1.852280
28	6	0	3.758742	-0.386421	0.660784
29	1	0	2.506136	1.107016	1.480010
30	6	0	4.091809	0.475724	-0.582091
31	6	0	3.751946	-1.634567	-0.299417
32	1	0	4.493314	-0.408276	1.485922
33	1	0	3.672630	-0.803034	-2.431825
34	1	0	-1.250917	-0.632047	1.842105
35	1	0	1.676116	0.616019	-1.988793
36	1	0	0.782582	-1.022741	0.328108
37	6	0	1.981401	-0.697775	2.469080
38	1	0	1.012091	-0.344811	2.856736
39	1	0	1.893325	-1.782477	2.299083
40	1	0	2.732871	-0.542878	3.262578
41	6	0	2.859302	-2.853676	-0.047629
42	1	0	3.199454	-3.411474	0.841681
43	1	0	1.800670	-2.603803	0.097152
44	1	0	2.924028	-3.544537	-0.906822
45	6	0	5.181946	-2.159009	-0.518106
46	1	0	5.208581	-2.859591	-1.370605
47	1	0	5.910488	-1.360020	-0.720861
48	1	0	5.529400	-2.708013	0.374135
49	1	0	3.679756	1.497268	-0.600525
50	1	0	5.165856	0.524193	-0.809239
51	1	0	-3.310363	0.982722	1.898979
52	1	0	-4.870724	0.115383	1.901994

53	6	0	-3.459321	-2.124972	-1.301374
54	1	0	-4.079941	-2.028670	-2.208539
55	1	0	-2.430199	-1.850249	-1.566490
56	1	0	-3.456648	-3.193736	-1.023160
57	6	0	-5.463849	-1.817539	0.112321
58	1	0	-6.088459	-1.694553	-0.789277
59	1	0	-5.442469	-2.893470	0.357455
60	1	0	-5.969315	-1.297418	0.939599
61	1	0	-1.115867	-0.450826	-1.116256
62	1	0	-2.689286	1.927706	-0.276112
63	6	0	-2.781625	1.048222	-2.226929
64	1	0	-1.859868	1.478919	-2.648971
65	1	0	-2.934575	0.074132	-2.716048
66	1	0	-3.620334	1.703536	-2.518179
67	1	0	-0.975569	2.631560	-1.282295

2a · 3 (*RR*)

Sum of electronic and zero-point Energies= -1611.385504
Sum of electronic and thermal Energies= -1611.342784
Sum of electronic and thermal Enthalpies= -1611.341840
Sum of electronic and thermal Free Energies= -1611.463621

Standard orientation:

Center Number	Atomic Number	Atomic Type	Coordinates (Angstroms) X	Y	Z
1	6	0	-4.523536	-0.309900	0.192387
2	7	0	-4.234312	-0.166724	1.504195
3	6	0	-2.949537	-0.090888	1.827545
4	6	0	-1.922178	-0.154093	0.864850
5	6	0	-2.338585	-0.314124	-0.475529
6	7	0	-3.618037	-0.390211	-0.811279
7	1	0	-2.708582	0.024986	2.890842
8	1	0	-1.601697	-0.378919	-1.282602
9	6	0	-5.904176	-0.382401	-0.169411
10	6	0	-7.084058	-0.441904	-0.479702
11	6	0	-8.503425	-0.504652	-0.856117
12	6	0	-8.968341	0.918916	-1.249455
13	1	0	-8.854794	1.619385	-0.407969
14	1	0	-10.031977	0.887124	-1.535157
15	1	0	-8.389593	1.302279	-2.103647
16	6	0	-9.328464	-1.014855	0.347304
17	1	0	-9.006932	-2.024093	0.647353
18	1	0	-10.394211	-1.057948	0.071262
19	1	0	-9.220221	-0.344129	1.213453
20	6	0	-8.667113	-1.462025	-2.059143
21	1	0	-8.081928	-1.113851	-2.923993
22	1	0	-9.728493	-1.506448	-2.351334
23	1	0	-8.335323	-2.479777	-1.802138
24	6	0	-0.533426	-0.064436	1.266860
25	8	0	0.398711	-0.124811	0.439487
26	1	0	-0.315276	0.057689	2.338184
27	5	0	1.980797	0.020023	0.759880
28	6	0	2.161462	-0.285658	2.362296
29	1	0	1.715548	0.505930	3.001044
30	1	0	3.241032	-0.213296	2.598629
31	6	0	1.674084	-1.592166	2.910250
32	6	0	0.965757	-2.659788	2.462941
33	6	0	1.803914	-2.172868	4.314245
34	6	0	0.994223	-3.397476	3.783811
35	1	0	0.017217	-3.557345	4.274127
36	1	0	1.542782	-4.355842	3.791141
37	6	0	2.714534	-1.145865	-0.184180
38	6	0	4.197734	-0.845759	-0.594956
39	6	0	1.867418	-1.700914	-1.374668
40	1	0	2.799712	-1.995942	0.511929
41	6	0	4.596521	-1.576137	-1.900360
42	1	0	4.299707	0.228388	-0.836872
43	6	0	2.702984	-2.241977	-2.546924
44	1	0	1.200872	-0.926747	-1.791888
45	6	0	3.637649	-1.105085	-3.021108
46	6	0	3.984913	-3.014712	-2.086180
47	1	0	2.058953	-2.736800	-3.296338
48	1	0	4.035380	-1.253773	-4.034650
49	1	0	5.683174	-1.477768	-2.076211
50	6	0	3.897673	-4.023101	-0.935793
51	1	0	4.905037	-4.364707	-0.641121
52	1	0	3.338680	-4.916628	-1.266188

53	1	0	3.399869	-3.634693	-0.039190
54	6	0	4.660014	-3.744765	-3.261866
55	1	0	4.071304	-4.632114	-3.552709
56	1	0	5.664691	-4.095472	-2.968632
57	1	0	4.777236	-3.116210	-4.156873
58	6	0	2.294829	1.600679	0.341149
59	6	0	1.703099	2.673040	1.319598
60	6	0	1.998695	1.908880	-1.166040
61	6	0	1.129729	3.900452	0.578743
62	6	0	1.322203	3.262814	-1.420931
63	1	0	1.343845	1.136152	-1.600354
64	6	0	0.109916	3.400023	-0.472073
65	6	0	2.007814	4.416273	-0.620420
66	1	0	1.161828	3.424349	-2.501850
67	1	0	0.792385	4.661598	1.305334
68	1	0	3.249993	-0.077523	-2.939336
69	1	0	0.825324	2.235094	1.841211
70	1	0	3.392172	1.661329	0.465571
71	1	0	-0.609846	4.168051	-0.788190
72	1	0	-0.442865	2.477809	-0.227982
73	6	0	1.538905	5.803929	-1.087508
74	1	0	1.980617	6.050331	-2.068541
75	1	0	1.862229	6.580570	-0.372954
76	1	0	0.445599	5.879245	-1.186921
77	6	0	3.536566	4.442938	-0.549146
78	1	0	3.949772	4.686637	-1.543908
79	1	0	3.975709	3.487375	-0.233800
80	1	0	3.887289	5.223911	0.147030
81	1	0	0.534054	-2.924857	1.492406
82	1	0	1.310362	-1.596416	5.117031
83	1	0	2.836254	-2.398211	4.635087
84	1	0	1.196675	-2.492076	-0.993083
85	1	0	2.938664	1.861655	-1.741601
86	6	0	2.691076	3.093065	2.418997
87	1	0	3.070445	2.225620	2.979836
88	1	0	3.562621	3.620055	2.001514
89	1	0	2.207353	3.772057	3.142741
90	6	0	5.190545	-1.127975	0.541129
91	1	0	4.981175	-0.505666	1.425780
92	1	0	5.158568	-2.181916	0.862768
93	1	0	6.223871	-0.906429	0.222474

2a · 3 (*RS*)

Sum of electronic and zero-point Energies= -1611.388601
Sum of electronic and thermal Energies= -1611.346464
Sum of electronic and thermal Enthalpies= -1611.345520
Sum of electronic and thermal Free Energies= -1611.464039

Standard orientation:

Center Number	Atomic Number	Atomic Type	Coordinates (Angstroms) X	Y	Z
1	6	0	4.637009	-0.362703	-0.271864
2	7	0	4.190411	-1.626043	-0.102231
3	6	0	2.877765	-1.785537	0.018783
4	6	0	1.974326	-0.704585	-0.027193
5	6	0	2.552699	0.571415	-0.204855
6	7	0	3.862589	0.744495	-0.326298
7	1	0	2.511668	-2.809703	0.156850
8	1	0	1.921803	1.464478	-0.254215
9	6	0	6.049695	-0.178339	-0.406180
10	6	0	7.254477	-0.017306	-0.524059
11	6	0	8.705129	0.181758	-0.659929
12	6	0	9.284869	0.541731	0.729540
13	1	0	9.106396	-0.267260	1.454435
14	1	0	10.372235	0.696715	0.641505
15	1	0	8.832083	1.466018	1.119903
16	6	0	9.353424	-1.121911	-1.178727
17	1	0	8.948673	-1.399455	-2.164207
18	1	0	10.441351	-0.978586	-1.278234
19	1	0	9.174719	-1.956159	-0.483034
20	6	0	8.965966	1.337586	-1.653284
21	1	0	8.507857	2.273834	-1.299286
22	1	0	10.051358	1.497452	-1.755500
23	1	0	8.554681	1.104304	-2.647494
24	6	0	0.546471	-0.920489	0.102282
25	8	0	-0.266495	0.036329	-0.000913
26	1	0	0.183728	-1.952419	0.176868
27	5	0	-1.779866	0.021327	0.493467
28	6	0	-1.529634	0.197140	2.173310
29	1	0	-1.535243	1.267806	2.430363
30	1	0	-2.426371	-0.259735	2.624666
31	6	0	-0.312126	-0.421517	2.715115
32	6	0	0.191945	-1.687637	2.835335
33	6	0	0.946746	0.205271	3.296482
34	6	0	1.490030	-1.247089	3.471780
35	1	0	2.417463	-1.476892	2.916573
36	1	0	1.634701	-1.561655	4.520255
37	6	0	-2.459320	-1.441726	0.120292
38	6	0	-3.720722	-1.752112	0.999955
39	6	0	-2.710132	-1.703521	-1.411881
40	1	0	-1.723408	-2.209461	0.422737
41	6	0	-4.841302	-2.504479	0.268666
42	1	0	-4.168417	-0.823483	1.392165
43	6	0	-4.008745	-2.501435	-1.667325
44	1	0	-2.882428	-0.731193	-1.909128
45	6	0	-5.181710	-1.725428	-1.022398
46	6	0	-4.299530	-3.656309	-0.640736
47	1	0	-4.089568	-2.762272	-2.738001
48	1	0	-6.164236	-1.979893	-1.443556
49	1	0	-5.663841	-2.753896	0.962560
50	6	0	-3.186310	-4.566128	-0.113577
51	1	0	-3.582140	-5.217874	0.685262
52	1	0	-2.809123	-5.227062	-0.912911

53	1	0	-2.330974	-4.017496	0.301010
54	6	0	-5.431655	-4.567666	-1.144681
55	1	0	-5.064682	-5.227759	-1.949641
56	1	0	-5.799762	-5.212715	-0.328098
57	1	0	-6.292801	-4.009331	-1.540787
58	6	0	-2.482405	1.368047	-0.147898
59	6	0	-3.796352	1.777478	0.601701
60	6	0	-1.500379	2.573199	-0.358341
61	6	0	-3.997123	3.293821	0.753557
62	1	0	-4.665813	1.343445	0.078921
63	6	0	-2.162322	3.936613	-0.061867
64	1	0	-0.678587	2.490385	0.379102
65	6	0	-2.719593	3.896809	1.381448
66	6	0	-3.650469	4.075577	-0.555610
67	1	0	-1.476863	4.761636	-0.328190
68	1	0	-4.968956	3.514298	1.230557
69	1	0	-5.085463	-0.628088	-0.983290
70	1	0	-3.822006	1.354878	1.621218
71	1	0	-2.799533	1.083729	-1.167165
72	6	0	-0.846442	2.550322	-1.747322
73	1	0	-0.361718	1.581588	-1.944729
74	1	0	-1.583684	2.719795	-2.548029
75	1	0	-0.075576	3.336024	-1.835138
76	6	0	-1.510897	-2.338379	-2.133569
77	1	0	-1.766572	-2.555560	-3.184971
78	1	0	-0.639572	-1.664842	-2.154256
79	1	0	-1.196599	-3.287640	-1.669329
80	1	0	-3.406306	-2.328607	1.887814
81	1	0	-2.874585	4.891088	1.823259
82	1	0	-2.174073	3.270066	2.106620
83	6	0	-4.115049	5.540524	-0.490300
84	1	0	-3.671341	6.121587	-1.317302
85	1	0	-5.212400	5.602209	-0.592777
86	1	0	-3.840559	6.040776	0.450389
87	6	0	-4.117199	3.510767	-1.900309
88	1	0	-3.690984	4.087545	-2.739116
89	1	0	-3.854248	2.455833	-2.048603
90	1	0	-5.215913	3.593996	-1.976563
91	1	0	-0.225216	-2.680083	2.634153
92	1	0	1.522933	0.844306	2.603956
93	1	0	0.791381	0.766305	4.234041

2a · 3 (*SS*)

Sum of electronic and zero-point Energies= -1611.388226
Sum of electronic and thermal Energies= -1611.345743
Sum of electronic and thermal Enthalpies= -1611.344798
Sum of electronic and thermal Free Energies= -1611.465783

Standard orientation:

Center Number	Atomic Number	Atomic Type	Coordinates (Angstroms) X	Y	Z
1	6	0	4.684166	-0.062273	0.189808
2	7	0	4.362577	0.351779	1.435413
3	6	0	3.070494	0.409738	1.729358
4	6	0	2.065945	0.062274	0.802775
5	6	0	2.516041	-0.359448	-0.468774
6	7	0	3.803953	-0.422016	-0.774059
7	1	0	2.802499	0.744545	2.738651
8	1	0	1.802140	-0.650219	-1.245594
9	6	0	6.072547	-0.126210	-0.140791
10	6	0	7.259223	-0.181773	-0.425108
11	6	0	8.685696	-0.244195	-0.773157
12	6	0	9.037528	0.999826	-1.625102
13	1	0	8.850208	1.928983	-1.065409
14	1	0	10.105332	0.964669	-1.894310
15	1	0	8.444960	1.025073	-2.552337
16	6	0	9.528539	-0.252619	0.522251
17	1	0	9.287222	-1.127938	1.144747
18	1	0	10.599088	-0.293933	0.265295
19	1	0	9.348323	0.656176	1.116733
20	6	0	8.949372	-1.531006	-1.589428
21	1	0	8.354221	-1.540893	-2.515335
22	1	0	10.016281	-1.580745	-1.859848
23	1	0	8.697523	-2.428881	-1.004392
24	6	0	0.671568	0.144646	1.180283
25	8	0	-0.251487	-0.171579	0.402344
26	1	0	0.439997	0.491425	2.198730
27	5	0	-1.847953	-0.016178	0.656214
28	6	0	-2.092311	0.074021	2.285829
29	1	0	-1.526604	0.928379	2.712331
30	1	0	-3.153298	0.335522	2.438014
31	6	0	-1.814190	-1.150531	3.092669
32	6	0	-2.579099	-2.049321	3.757150
33	6	0	-0.519970	-1.876372	3.458084
34	6	0	-1.418048	-2.901849	4.218519
35	1	0	-1.377737	-3.931845	3.820863
36	1	0	-1.247097	-2.948006	5.309169
37	6	0	-2.453659	-1.399550	-0.012354
38	6	0	-4.007401	-1.499296	0.182243
39	6	0	-1.981250	-1.710470	-1.476129
40	1	0	-2.028831	-2.210487	0.607724
41	6	0	-4.765786	-2.003851	-1.052880
42	1	0	-4.439359	-0.521260	0.445957
43	6	0	-3.130656	-2.224392	-2.367814
44	1	0	-1.658449	-0.762714	-1.949841
45	6	0	-4.293082	-1.205120	-2.289984
46	6	0	-4.088451	-3.265439	-1.678359
47	1	0	-2.748385	-2.497093	-3.368290
48	1	0	-4.987919	-1.263125	-3.139657
49	1	0	-5.850495	-2.062701	-0.851138
50	6	0	-3.560322	-4.349218	-0.734867
51	1	0	-4.408509	-4.864208	-0.249680
52	1	0	-2.991570	-5.115285	-1.289477

53	1	0	-2.913560	-3.956300	0.060537
54	6	0	-4.991473	-3.951017	-2.716696
55	1	0	-4.417542	-4.695765	-3.295078
56	1	0	-5.819058	-4.484191	-2.217255
57	1	0	-5.436004	-3.245155	-3.434429
58	6	0	-2.190233	1.417886	-0.149974
59	6	0	-3.640279	1.934758	0.158480
60	6	0	-1.098049	2.531527	0.006792
61	6	0	-3.738910	3.444134	0.420080
62	1	0	-4.307596	1.664003	-0.677016
63	6	0	-1.696858	3.932089	0.253130
64	1	0	-0.509480	2.330455	0.928774
65	6	0	-2.663835	3.839056	1.458126
66	6	0	-2.947033	4.276467	-0.637605
67	1	0	-0.893355	4.689645	0.289584
68	1	0	-4.786537	3.736857	0.611940
69	1	0	-4.015322	-0.150438	-2.129851
70	1	0	-4.060949	1.435886	1.046691
71	1	0	-2.178609	1.149552	-1.220987
72	6	0	-0.094512	2.542272	-1.155660
73	1	0	0.342480	1.545489	-1.323162
74	1	0	-0.571452	2.845937	-2.099564
75	1	0	0.732875	3.245883	-0.958657
76	6	0	-0.767881	-2.650868	-1.521710
77	1	0	-0.397213	-2.766537	-2.555435
78	1	0	0.060820	-2.260621	-0.911613
79	1	0	-1.009581	-3.655588	-1.142144
80	1	0	-4.223035	-2.158925	1.040419
81	1	0	-2.857029	4.806544	1.942235
82	1	0	-2.421124	3.095323	2.235057
83	6	0	-3.278647	5.775633	-0.561377
84	1	0	-2.555516	6.360804	-1.155452
85	1	0	-4.283223	5.969726	-0.975232
86	1	0	-3.260327	6.169785	0.465749
87	6	0	-3.033750	3.855137	-2.106399
88	1	0	-2.335106	4.439831	-2.729048
89	1	0	-2.820595	2.790208	-2.267569
90	1	0	-4.050831	4.052232	-2.488894
91	1	0	-3.660023	-2.134238	3.913297
92	1	0	0.050341	-2.306438	2.615793
93	1	0	0.180367	-1.312116	4.100629

2a · 3 (*SR*)

Sum of electronic and zero-point Energies= -1611.389771
Sum of electronic and thermal Energies= -1611.347403
Sum of electronic and thermal Enthalpies= -1611.346459
Sum of electronic and thermal Free Energies= -1611.467032

Standard orientation:

Center Number	Atomic Number	Atomic Type	Coordinates (Angstroms) X	Y	Z
1	6	0	4.650396	-0.014668	0.053475
2	7	0	4.328343	0.662303	1.177840
3	6	0	3.039206	0.734687	1.482097
4	6	0	2.037056	0.143830	0.685409
5	6	0	2.487441	-0.536089	-0.467664
6	7	0	3.772872	-0.617261	-0.781781
7	1	0	2.772077	1.281992	2.393789
8	1	0	1.775709	-1.019129	-1.144176
9	6	0	6.036599	-0.100341	-0.285030
10	6	0	7.220757	-0.173755	-0.575181
11	6	0	8.644648	-0.256487	-0.930931
12	6	0	8.961623	0.867136	-1.947864
13	1	0	8.761457	1.859743	-1.516150
14	1	0	10.026848	0.816236	-2.224886
15	1	0	8.358367	0.755639	-2.861781
16	6	0	9.500750	-0.071634	0.342639
17	1	0	9.284482	-0.858656	1.081424
18	1	0	10.569240	-0.126866	0.079551
19	1	0	9.307610	0.905985	0.810395
20	6	0	8.928164	-1.636613	-1.567827
21	1	0	8.323488	-1.784816	-2.475611
22	1	0	9.992890	-1.702043	-1.843585
23	1	0	8.701855	-2.451236	-0.862771
24	6	0	0.643172	0.263800	1.059617
25	8	0	-0.275565	-0.250954	0.379307
26	1	0	0.404211	0.840711	1.961806
27	5	0	-1.835914	-0.089936	0.707727
28	6	0	-1.961157	0.038833	2.362616
29	1	0	-1.721706	1.063520	2.703636
30	1	0	-3.034575	-0.107746	2.582065
31	6	0	-1.178395	-0.929842	3.173790
32	6	0	-1.310117	-2.434294	3.383750
33	6	0	-0.096036	-0.849544	3.992661
34	6	0	-0.074983	-2.322925	4.331689
35	1	0	-1.150913	-3.061295	2.490690
36	1	0	-2.246831	-2.756610	3.871057
37	1	0	0.520317	-0.010794	4.336801
38	1	0	0.814606	-2.891028	4.005344
39	1	0	-0.276567	-2.578005	5.387317
40	6	0	-2.511988	-1.469339	0.103684
41	6	0	-4.041338	-1.551873	0.445180
42	6	0	-2.190312	-1.768198	-1.403979
43	1	0	-2.038634	-2.295125	0.662385
44	6	0	-4.924996	-2.029364	-0.714914
45	1	0	-4.429520	-0.569368	0.759906
46	6	0	-3.429039	-2.252629	-2.186213
47	1	0	-1.900405	-0.818927	-1.895536
48	6	0	-4.563723	-1.219341	-1.981536
49	6	0	-4.331540	-3.290611	-1.421473
50	1	0	-3.150233	-2.515282	-3.222853
51	1	0	-5.338987	-1.255903	-2.759746
52	1	0	-5.985478	-2.076065	-0.408469

53	6	0	-3.730887	-4.397080	-0.550598
54	1	0	-4.535129	-4.907575	0.008573
55	1	0	-3.231976	-5.162112	-1.169962
56	1	0	-3.001741	-4.027056	0.182010
57	6	0	-5.342266	-3.947147	-2.375874
58	1	0	-4.839208	-4.691332	-3.017421
59	1	0	-6.125667	-4.475720	-1.805350
60	1	0	-5.843245	-3.224287	-3.037181
61	6	0	-2.291773	1.327879	-0.082121
62	6	0	-3.635655	1.910513	0.475693
63	6	0	-1.173745	2.409648	-0.233682
64	6	0	-3.670209	3.442411	0.582509
65	1	0	-4.468248	1.570798	-0.163402
66	6	0	-1.696780	3.848600	-0.033511
67	1	0	-0.433399	2.289324	0.587468
68	6	0	-2.407530	3.912688	1.339832
69	6	0	-3.094993	4.137135	-0.693584
70	1	0	-0.893242	4.578884	-0.237444
71	1	0	-4.656259	3.785251	0.943034
72	1	0	-4.257597	-0.170444	-1.835610
73	1	0	-3.855120	1.519586	1.482997
74	1	0	-2.503348	0.997993	-1.113605
75	6	0	-0.389527	2.256736	-1.544316
76	1	0	0.008281	1.235602	-1.657982
77	1	0	-1.024629	2.455620	-2.421078
78	1	0	0.461904	2.957989	-1.582025
79	6	0	-0.999761	-2.722292	-1.578678
80	1	0	-0.731972	-2.827665	-2.644726
81	1	0	-0.113343	-2.347892	-1.045217
82	1	0	-1.215645	-3.729647	-1.190510
83	1	0	-4.187375	-2.217977	1.313819
84	1	0	-2.491654	4.930511	1.745246
85	1	0	-2.022598	3.247261	2.130945
86	6	0	-3.391072	5.646058	-0.712335
87	1	0	-2.793101	6.145729	-1.493960
88	1	0	-4.455379	5.826540	-0.941968
89	1	0	-3.168611	6.142665	0.243947
90	6	0	-3.469627	3.576715	-2.067699
91	1	0	-2.893295	4.073087	-2.867119
92	1	0	-3.308718	2.494558	-2.157639
93	1	0	-4.537599	3.771775	-2.269729

TS4(*RR*)

Sum of electronic and zero-point Energies= -1611.394599
Sum of electronic and thermal Energies= -1611.353469
Sum of electronic and thermal Enthalpies= -1611.352525
Sum of electronic and thermal Free Energies= -1611.468818

Standard orientation:

Center Number	Atomic Number	Atomic Type	Coordinates (Angstroms) X	Y	Z
1	6	0	4.654099	-0.301447	-0.111599
2	7	0	4.218791	-1.561352	0.090691
3	6	0	2.914587	-1.720482	0.297496
4	6	0	2.010495	-0.642834	0.317465
5	6	0	2.573182	0.625609	0.073298
6	7	0	3.873716	0.800001	-0.138254
7	1	0	2.560037	-2.747084	0.446424
8	1	0	1.939584	1.517676	0.050773
9	6	0	6.061158	-0.114401	-0.318858
10	6	0	7.258669	0.047945	-0.491411
11	6	0	8.701434	0.251168	-0.699169
12	6	0	9.261496	1.088328	0.475146
13	1	0	9.115887	0.569552	1.435251
14	1	0	10.341507	1.251047	0.328498
15	1	0	8.766336	2.069896	0.532164
16	6	0	9.409934	-1.121055	-0.753483
17	1	0	9.020377	-1.734052	-1.580890
18	1	0	10.490992	-0.974475	-0.908976
19	1	0	9.266460	-1.676962	0.185957
20	6	0	8.914174	1.009209	-2.030495
21	1	0	8.413105	1.989227	-2.011605
22	1	0	9.991831	1.171954	-2.193549
23	1	0	8.517730	0.433173	-2.880834
24	6	0	0.579470	-0.852804	0.562454
25	8	0	-0.247130	0.080241	0.244867
26	1	0	0.235136	-1.895258	0.589245
27	5	0	-1.754731	0.043825	0.526864
28	6	0	0.741603	-2.164910	3.375696
29	6	0	-0.813924	-2.229886	3.250769
30	6	0	0.625830	-0.752943	2.834727
31	6	0	-0.766820	-0.779353	2.801259
32	1	0	-1.349893	-2.384880	4.201788
33	1	0	-1.207293	-2.948348	2.512283
34	1	0	1.330940	0.082596	2.843144
35	6	0	-2.419388	-1.384472	0.032784
36	6	0	-3.786333	-1.702470	0.728444
37	6	0	-2.471465	-1.567990	-1.529323
38	1	0	-1.735249	-2.177895	0.383388
39	6	0	-4.815973	-2.393276	-0.177370
40	1	0	-4.263370	-0.781518	1.102686
41	6	0	-3.739069	-2.321559	-1.988903
42	1	0	-2.558014	-0.570482	-1.998350
43	6	0	-4.971183	-1.547055	-1.462150
44	6	0	-4.182419	-3.514576	-1.065179
45	1	0	-3.685337	-2.531301	-3.072377
46	1	0	-5.895692	-1.756867	-2.017812
47	1	0	-5.726503	-2.654334	0.390995
48	6	0	-3.164893	-4.475984	-0.444315
49	1	0	-3.672936	-5.149907	0.267999
50	1	0	-2.703893	-5.112640	-1.218930
51	1	0	-2.356510	-3.969263	0.098267
52	6	0	-5.256543	-4.373208	-1.754583

53	1	0	-4.800699	-5.003570	-2.537587
54	1	0	-5.738793	-5.046593	-1.024926
55	1	0	-6.048801	-3.775921	-2.229682
56	6	0	-2.399743	1.439941	-0.075884
57	6	0	-3.780864	1.834611	0.550077
58	6	0	-1.387910	2.636998	-0.131974
59	6	0	-3.972503	3.343708	0.767751
60	1	0	-4.595153	1.450801	-0.088094
61	6	0	-2.056398	3.993819	0.176249
62	1	0	-0.638319	2.499732	0.671663
63	6	0	-2.755603	3.884279	1.552886
64	6	0	-3.485267	4.188623	-0.454039
65	1	0	-1.336303	4.818602	0.026564
66	1	0	-4.984015	3.557060	1.157444
67	1	0	-4.860144	-0.455397	-1.356847
68	1	0	-3.926703	1.357174	1.534092
69	1	0	-2.614892	1.205130	-1.134043
70	6	0	-0.606903	2.672369	-1.453273
71	1	0	-0.118458	1.705830	-1.649764
72	1	0	-1.262974	2.893311	-2.309992
73	1	0	0.177737	3.448800	-1.428713
74	6	0	-1.193069	-2.195482	-2.104562
75	1	0	-0.314216	-1.555493	-1.929968
76	1	0	-0.982710	-3.187094	-1.671369
77	1	0	-1.283600	-2.325215	-3.196802
78	1	0	-3.600442	-2.324808	1.621779
79	6	0	-3.933967	5.656363	-0.354530
80	1	0	-3.401427	6.272216	-1.099832
81	1	0	-5.014522	5.744476	-0.562160
82	1	0	-3.748514	6.100132	0.635030
83	6	0	-3.822285	3.704061	-1.866927
84	1	0	-3.307208	4.316165	-2.627058
85	1	0	-3.558953	2.653722	-2.045415
86	1	0	-4.906785	3.810471	-2.047033
87	1	0	-2.295048	3.206786	2.291515
88	1	0	-2.939367	4.855122	2.034118
89	1	0	1.117084	-2.233117	4.409976
90	1	0	1.304222	-2.885992	2.758527
91	6	0	-1.730108	0.148128	2.296465
92	1	0	-1.453549	1.193879	2.491980
93	1	0	-2.760525	-0.061499	2.611104

TS5(*SS*)

Sum of electronic and zero-point Energies= -1611.385446
Sum of electronic and thermal Energies= -1611.344133
Sum of electronic and thermal Enthalpies= -1611.343189
Sum of electronic and thermal Free Energies= -1611.460132

Standard orientation:

Center Number	Atomic Number	Atomic Type	Coordinates (Angstroms) X	Y	Z
1	6	0	-4.684406	-0.318066	-0.133378
2	7	0	-4.253124	-1.594570	-0.089862
3	6	0	-2.950216	-1.782459	0.102087
4	6	0	-2.044893	-0.718322	0.263218
5	6	0	-2.603113	0.572468	0.179308
6	7	0	-3.901853	0.776246	-0.015543
7	1	0	-2.598903	-2.820639	0.119317
8	1	0	-1.966253	1.457791	0.273239
9	6	0	-6.089475	-0.102819	-0.325324
10	6	0	-7.285336	0.081496	-0.486896
11	6	0	-8.725725	0.310311	-0.683783
12	6	0	-9.457975	-1.049420	-0.737198
13	1	0	-9.318992	-1.609792	0.200273
14	1	0	-10.537200	-0.883974	-0.886290
15	1	0	-9.083486	-1.667155	-1.568019
16	6	0	-9.264423	1.155030	0.494785
17	1	0	-8.752041	2.127795	0.550338
18	1	0	-10.342343	1.336504	0.355035
19	1	0	-9.121999	0.632368	1.453266
20	6	0	-8.932588	1.074293	-2.012948
21	1	0	-8.551206	0.492868	-2.866481
22	1	0	-10.008265	1.255773	-2.169127
23	1	0	-8.414673	2.045526	-1.995285
24	6	0	-0.611973	-0.958348	0.480104
25	8	0	0.205872	0.010372	0.277989
26	1	0	-0.276449	-1.998571	0.386339
27	5	0	1.722736	0.085302	0.578081
28	6	0	1.754996	-0.335378	2.297628
29	1	0	2.768162	-0.697668	2.518787
30	1	0	1.582192	0.639693	2.775171
31	6	0	0.723899	-1.264469	2.665680
32	6	0	-0.658880	-1.129964	2.742772
33	6	0	0.655842	-2.758772	2.940729
34	6	0	-0.886739	-2.583391	3.104678
35	6	0	2.048409	1.707375	0.465013
36	6	0	3.218652	2.219122	1.372348
37	6	0	2.116156	2.292887	-0.989628
38	1	0	1.134464	2.150021	0.900961
39	6	0	3.980851	3.426771	0.804120
40	1	0	3.964691	1.427237	1.553705
41	6	0	3.057638	3.515684	-1.089514
42	1	0	2.574792	1.538552	-1.657033
43	6	0	4.463666	3.070427	-0.620791
44	6	0	3.032880	4.486333	0.148552
45	1	0	2.970063	3.985733	-2.085833
46	1	0	5.276514	3.714991	-0.983349
47	1	0	4.725671	3.798895	1.530469
48	6	0	1.728454	4.871212	0.853198
49	1	0	1.954136	5.455107	1.763181
50	1	0	1.108540	5.512995	0.203657
51	1	0	1.119279	4.010036	1.154056
52	6	0	3.775261	5.796553	-0.168727

53	1	0	3.158373	6.438782	-0.820918
54	1	0	3.975152	6.360634	0.758860
55	1	0	4.738711	5.638523	-0.675597
56	6	0	2.645269	-0.909076	-0.367722
57	6	0	4.149490	-0.894667	0.068444
58	6	0	2.119298	-2.349909	-0.698463
59	6	0	4.885864	-2.228006	-0.125677
60	1	0	4.678978	-0.097719	-0.479951
61	6	0	3.257306	-3.389986	-0.787450
62	1	0	1.498336	-2.711281	0.141681
63	6	0	4.050626	-3.343813	0.541906
64	6	0	4.565583	-2.890046	-1.504469
65	1	0	2.857525	-4.365649	-1.118369
66	1	0	5.950279	-2.131664	0.153375
67	1	0	4.735981	2.018901	-0.807435
68	1	0	4.251728	-0.635542	1.136464
69	1	0	2.624023	-0.389653	-1.342687
70	6	0	1.237424	-2.370519	-1.956347
71	1	0	0.428619	-1.624333	-1.906635
72	1	0	1.823197	-2.143828	-2.860373
73	1	0	0.773750	-3.361801	-2.101412
74	1	0	2.814500	2.479358	2.366400
75	6	0	0.726543	2.587663	-1.570514
76	1	0	0.807091	2.970101	-2.602967
77	1	0	0.105987	1.679213	-1.597810
78	1	0	0.185499	3.343581	-0.977614
79	6	0	5.505453	-4.068945	-1.808560
80	1	0	5.127207	-4.650927	-2.666749
81	1	0	6.511453	-3.700895	-2.074373
82	1	0	5.618127	-4.762239	-0.961705
83	6	0	4.522056	-2.006111	-2.753966
84	1	0	4.155356	-2.573376	-3.626615
85	1	0	3.890273	-1.115986	-2.640421
86	1	0	5.542035	-1.660729	-2.998693
87	1	0	4.622150	-4.259645	0.747447
88	1	0	3.486003	-3.067795	1.448253
89	1	0	0.981839	-3.423416	2.124692
90	1	0	1.186013	-3.057796	3.860355
91	1	0	-1.513614	-3.171960	2.413265
92	1	0	-1.294726	-0.248016	2.858147
93	1	0	-1.257602	-2.750968	4.129233

TS6(*RS*)

Sum of electronic and zero-point Energies= -1611.386045
Sum of electronic and thermal Energies= -1611.344755
Sum of electronic and thermal Enthalpies= -1611.343810
Sum of electronic and thermal Free Energies= -1611.460816

Standard orientation:

Center Number	Atomic Number	Atomic Type	Coordinates (Angstroms) X	Y	Z
1	6	0	4.584449	-0.452420	-0.078783
2	7	0	4.179054	-1.642531	0.413150
3	6	0	2.882341	-1.774288	0.671878
4	6	0	1.951163	-0.742094	0.450212
5	6	0	2.484705	0.450517	-0.074401
6	7	0	3.781658	0.599749	-0.335353
7	1	0	2.554382	-2.741881	1.070395
8	1	0	1.830173	1.298137	-0.297667
9	6	0	5.984220	-0.294890	-0.350947
10	6	0	7.175383	-0.156089	-0.578894
11	6	0	8.610194	0.013227	-0.859527
12	6	0	9.420847	-0.965528	0.020138
13	1	0	9.140292	-2.009431	-0.188692
14	1	0	10.496512	-0.846158	-0.187437
15	1	0	9.250438	-0.768747	1.089940
16	6	0	8.863250	-0.291449	-2.355411
17	1	0	8.290034	0.391895	-3.000674
18	1	0	9.934810	-0.164719	-2.579344
19	1	0	8.576486	-1.325240	-2.602428
20	6	0	9.018706	1.470805	-0.544987
21	1	0	8.841024	1.710809	0.514743
22	1	0	10.091413	1.608199	-0.756686
23	1	0	8.448468	2.182516	-1.161640
24	6	0	0.527187	-0.922198	0.743399
25	8	0	-0.309388	-0.061286	0.255658
26	1	0	0.188071	-1.952840	0.899620
27	5	0	-1.798031	-0.038959	0.566071
28	6	0	-1.808141	0.219676	2.376914
29	1	0	-2.328295	1.167212	2.560400
30	1	0	-2.399216	-0.639069	2.719211
31	6	0	-0.471287	0.210805	2.859952
32	6	0	0.525711	1.319473	3.132016
33	6	0	0.516559	-0.773796	2.964877
34	6	0	1.573362	0.207288	3.440891
35	1	0	0.258698	1.985944	3.969013
36	1	0	0.757821	1.942958	2.251622
37	1	0	0.441377	-1.856426	3.105738
38	1	0	1.817123	0.113758	4.512149
39	1	0	2.517311	0.251932	2.874031
40	6	0	-2.470134	-1.526462	0.291763
41	6	0	-3.866048	-1.729848	0.978423
42	6	0	-2.466557	-1.941958	-1.228604
43	1	0	-1.804198	-2.256112	0.788976
44	6	0	-4.889398	-2.494846	0.127989
45	1	0	-4.324624	-0.761711	1.237419
46	6	0	-3.746080	-2.707426	-1.630032
47	1	0	-2.491761	-1.022465	-1.842327
48	6	0	-4.965461	-1.827734	-1.264480
49	6	0	-4.264536	-3.746831	-0.570715
50	1	0	-3.661795	-3.066139	-2.671651
51	1	0	-5.876915	-2.082511	-1.823023
52	1	0	-5.829056	-2.646264	0.688622

53	6	0	-3.302328	-4.635545	0.222074
54	1	0	-3.858525	-5.189803	0.998711
55	1	0	-2.828931	-5.385728	-0.434367
56	1	0	-2.503078	-4.075113	0.724152
57	6	0	-5.340482	-4.661803	-1.178863
58	1	0	-4.877628	-5.408928	-1.846565
59	1	0	-5.873895	-5.211521	-0.384073
60	1	0	-6.092190	-4.113476	-1.765925
61	6	0	-2.456992	1.256320	-0.210410
62	6	0	-3.846653	1.717260	0.348527
63	6	0	-1.458589	2.446685	-0.435863
64	6	0	-4.047782	3.239840	0.375634
65	1	0	-4.653228	1.254295	-0.245087
66	6	0	-2.139719	3.825065	-0.303204
67	1	0	-0.701577	2.423873	0.370472
68	6	0	-2.832545	3.886699	1.079543
69	6	0	-3.572239	3.924103	-0.946899
70	1	0	-1.428134	4.630389	-0.560894
71	1	0	-5.059646	3.493883	0.739107
72	1	0	-4.817465	-0.736283	-1.305334
73	1	0	-4.001581	1.361980	1.382096
74	1	0	-2.667094	0.874022	-1.226342
75	6	0	-0.689479	2.313921	-1.758051
76	1	0	-0.197738	1.332127	-1.831931
77	1	0	-1.354190	2.418386	-2.629987
78	1	0	0.090559	3.090859	-1.842402
79	6	0	-4.034421	5.388212	-1.035757
80	1	0	-5.116221	5.439271	-1.249178
81	1	0	-3.850759	5.956778	-0.111812
82	1	0	-3.509281	5.908087	-1.855691
83	6	0	-3.908410	3.256003	-2.282827
84	1	0	-3.631615	2.194731	-2.323803
85	1	0	-4.994838	3.324498	-2.469503
86	1	0	-3.403806	3.770056	-3.118795
87	1	0	-2.360686	3.314537	1.895938
88	1	0	-3.024353	4.909409	1.433285
89	1	0	-3.725830	-2.259552	1.937371
90	6	0	-1.196905	-2.696073	-1.652548
91	1	0	-0.300380	-2.064558	-1.558934
92	1	0	-1.033941	-3.609478	-1.057728
93	1	0	-1.265603	-2.999546	-2.711455

TS7(*SR*)

Sum of electronic and zero-point Energies= -1611.384351
Sum of electronic and thermal Energies= -1611.343038
Sum of electronic and thermal Enthalpies= -1611.342094
Sum of electronic and thermal Free Energies= -1611.458701

Standard orientation:

Center Number	Atomic Number	Atomic Type	Coordinates (Angstroms) X	Y	Z
1	6	0	-4.636919	-0.476509	-0.067068
2	7	0	-4.236318	-1.681540	0.391168
3	6	0	-2.940222	-1.825066	0.646810
4	6	0	-2.006172	-0.790129	0.455107
5	6	0	-2.534384	0.418667	-0.036557
6	7	0	-3.830456	0.579819	-0.294106
7	1	0	-2.615396	-2.805271	1.016382
8	1	0	-1.876688	1.271383	-0.230295
9	6	0	-6.035911	-0.306573	-0.335802
10	6	0	-7.225968	-0.157567	-0.563138
11	6	0	-8.660996	0.017352	-0.839326
12	6	0	-9.469182	-0.413132	0.407076
13	1	0	-9.204744	0.201644	1.281318
14	1	0	-10.546090	-0.289271	0.208736
15	1	0	-9.280267	-1.468901	0.655346
16	6	0	-8.941977	1.501741	-1.164470
17	1	0	-8.370949	1.827363	-2.047611
18	1	0	-10.015070	1.638937	-1.374288
19	1	0	-8.668479	2.150000	-0.317624
20	6	0	-9.047981	-0.869578	-2.046488
21	1	0	-8.851876	-1.932081	-1.835391
22	1	0	-10.122204	-0.749624	-2.261191
23	1	0	-8.480250	-0.583360	-2.945250
24	6	0	-0.581763	-0.978801	0.753127
25	8	0	0.260108	-0.138414	0.247888
26	1	0	-0.257287	-2.009329	0.944874
27	5	0	1.761174	-0.008744	0.561027
28	6	0	1.862135	-0.356164	2.311581
29	1	0	2.130339	-1.418987	2.398419
30	1	0	2.676708	0.294090	2.654541
31	6	0	0.591818	-0.057713	2.884062
32	6	0	-0.091832	1.242019	3.255615
33	6	0	-0.607077	-0.775436	2.953534
34	6	0	-1.402577	0.418957	3.448754
35	1	0	-0.119458	1.993188	2.448137
36	1	0	0.308631	1.722239	4.163946
37	1	0	-0.796637	-1.846254	3.072185
38	1	0	-2.257501	0.733839	2.827193
39	1	0	-1.744487	0.343255	4.494198
40	6	0	2.099145	1.577064	0.237879
41	6	0	3.312225	2.173330	1.028660
42	6	0	2.132229	1.954099	-1.288703
43	1	0	1.211753	2.104107	0.629573
44	6	0	4.101511	3.251786	0.270554
45	1	0	4.036613	1.388106	1.304076
46	6	0	3.120384	3.104089	-1.589513
47	1	0	2.530659	1.093644	-1.859136
48	6	0	4.521847	2.668761	-1.098370
49	6	0	3.174878	4.244328	-0.507123
50	1	0	3.019524	3.428270	-2.641075
51	1	0	5.347926	3.215147	-1.574401
52	1	0	4.883950	3.692110	0.914450

53	6	0	1.910515	4.780744	0.170560
54	1	0	2.188414	5.477299	0.981417
55	1	0	1.293678	5.349425	-0.546375
56	1	0	1.280003	3.996935	0.608068
57	6	0	3.955780	5.461675	-1.032517
58	1	0	3.343116	6.027862	-1.755256
59	1	0	4.207170	6.145521	-0.203236
60	1	0	4.895526	5.190615	-1.536043
61	6	0	2.688052	-1.143849	-0.214598
62	6	0	4.171143	-1.149632	0.280038
63	6	0	2.088908	-2.582824	-0.359969
64	6	0	4.847797	-2.529274	0.245918
65	1	0	4.756665	-0.435504	-0.322631
66	6	0	3.174716	-3.682104	-0.314266
67	1	0	1.454104	-2.804943	0.520369
68	6	0	3.952937	-3.527724	1.014809
69	6	0	4.515728	-3.327538	-1.057259
70	1	0	2.729867	-4.667375	-0.542786
71	1	0	5.911315	-2.450552	0.533417
72	1	0	4.744164	1.589722	-1.134341
73	1	0	4.246478	-0.791421	1.321437
74	1	0	2.720582	-0.755577	-1.248205
75	6	0	1.198051	-2.715551	-1.603823
76	1	0	0.426574	-1.931064	-1.638605
77	1	0	1.787755	-2.629955	-2.529985
78	1	0	0.688139	-3.694298	-1.624409
79	1	0	2.946074	2.589014	1.984608
80	6	0	0.736141	2.233453	-1.861144
81	1	0	0.796406	2.474538	-2.936689
82	1	0	0.081710	1.356194	-1.751409
83	1	0	0.246714	3.083363	-1.357588
84	6	0	5.402868	-4.575526	-1.206976
85	1	0	5.009489	-5.233173	-2.001278
86	1	0	6.428767	-4.286197	-1.493197
87	1	0	5.470786	-5.172790	-0.285581
88	6	0	4.532982	-2.596825	-2.402943
89	1	0	4.149310	-3.246889	-3.207916
90	1	0	3.945573	-1.670110	-2.410295
91	1	0	5.572082	-2.333418	-2.668185
92	1	0	4.478753	-4.440080	1.328596
93	1	0	3.389770	-3.131606	1.877136

6a(*RR*)

Sum of electronic and zero-point Energies= -1611.444149
Sum of electronic and thermal Energies= -1611.401622
Sum of electronic and thermal Enthalpies= -1611.400678
Sum of electronic and thermal Free Energies= -1611.525512

Standard orientation:

Center Number	Atomic Number	Atomic Type	Coordinates (Angstroms) X	Y	Z
1	6	0	-4.197898	0.352545	0.175823
2	7	0	-3.654861	1.577984	0.062531
3	6	0	-2.398060	1.718404	0.489800
4	6	0	-1.654460	0.665419	1.037999
5	6	0	-2.321598	-0.569015	1.096346
6	7	0	-3.575160	-0.733893	0.674011
7	1	0	-1.956088	2.716801	0.387020
8	1	0	-1.819019	-1.457109	1.493907
9	6	0	-5.553837	0.186258	-0.274291
10	6	0	-6.703832	0.045982	-0.657183
11	6	0	-8.093069	-0.120079	-1.116550
12	6	0	-9.049550	0.257679	0.039251
13	1	0	-8.900827	1.303520	0.349483
14	1	0	-10.093737	0.138618	-0.292657
15	1	0	-8.887273	-0.390347	0.914381
16	6	0	-8.341374	0.809298	-2.326651
17	1	0	-7.666603	0.560734	-3.160414
18	1	0	-9.380519	0.694726	-2.675482
19	1	0	-8.180453	1.863836	-2.054255
20	6	0	-8.321404	-1.592866	-1.526080
21	1	0	-8.144641	-2.270827	-0.676814
22	1	0	-9.360680	-1.726543	-1.867605
23	1	0	-7.646955	-1.883517	-2.346374
24	6	0	-0.238718	0.850919	1.544357
25	8	0	0.576992	-0.191616	1.011740
26	1	0	0.124994	1.834637	1.207087
27	5	0	1.623750	-0.087219	0.123573
28	6	0	2.292442	0.712308	3.966973
29	1	0	2.497300	-0.320079	3.669489
30	1	0	3.082849	1.244075	4.507449
31	6	0	1.120343	1.296898	3.703393
32	6	0	0.501117	2.628656	4.087307
33	6	0	-0.183511	0.801590	3.086330
34	6	0	-0.889247	2.008510	3.779044
35	1	0	0.766695	3.443246	3.391548
36	1	0	0.684178	2.981399	5.114466
37	1	0	-0.507462	-0.200816	3.410353
38	1	0	-1.570323	2.605672	3.154103
39	1	0	-1.425079	1.699682	4.687577
40	6	0	2.197205	1.311933	-0.393264
41	6	0	3.601235	1.576616	0.262125
42	6	0	2.202098	1.438202	-1.957805
43	1	0	1.540952	2.120519	-0.036532
44	6	0	4.613520	2.219509	-0.696579
45	1	0	4.054178	0.640106	0.632398
46	6	0	3.469404	2.154062	-2.471484
47	1	0	2.257437	0.424711	-2.395747
48	6	0	4.696467	1.350560	-1.972836
49	6	0	3.977222	3.345450	-1.578520
50	1	0	3.384152	2.349600	-3.555093
51	1	0	5.607018	1.523439	-2.562570
52	1	0	5.550863	2.461709	-0.165606

53	6	0	3.011525	4.347721	-0.939026
54	1	0	3.566722	5.018453	-0.259998
55	1	0	2.542861	4.983513	-1.709482
56	1	0	2.207542	3.881042	-0.355982
57	6	0	5.051032	4.161267	-2.318807
58	1	0	4.585339	4.793668	-3.094054
59	1	0	5.578771	4.829653	-1.616882
60	1	0	5.807193	3.533604	-2.812965
61	6	0	2.249257	-1.496401	-0.309656
62	6	0	3.161650	-2.067432	0.837722
63	6	0	1.163109	-2.514213	-0.813158
64	6	0	2.999242	-3.576653	1.062151
65	1	0	4.214302	-1.831965	0.606372
66	6	0	1.429301	-3.944536	-0.301042
67	1	0	0.195109	-2.232259	-0.362173
68	6	0	1.495097	-3.884299	1.245694
69	6	0	2.940415	-4.378327	-0.280171
70	1	0	0.718012	-4.653280	-0.761304
71	1	0	3.705877	-3.932606	1.832723
72	1	0	4.557491	0.263664	-1.846357
73	1	0	2.943903	-1.572394	1.799669
74	1	0	2.917408	-1.308865	-1.165980
75	6	0	0.972485	-2.441507	-2.333118
76	1	0	0.683302	-1.424677	-2.648146
77	1	0	1.889009	-2.710881	-2.881771
78	1	0	0.171669	-3.126250	-2.660304
79	6	0	3.072896	-5.892254	-0.044768
80	1	0	2.808208	-6.447962	-0.960815
81	1	0	4.113941	-6.152765	0.213211
82	1	0	2.426848	-6.261737	0.765505
83	6	0	3.899390	-4.005552	-1.414255
84	1	0	3.647707	-4.557318	-2.336119
85	1	0	3.903008	-2.934936	-1.655155
86	1	0	4.931036	-4.287931	-1.139523
87	1	0	0.886141	-3.106041	1.734723
88	1	0	1.301530	-4.849321	1.733965
89	6	0	0.900871	2.062789	-2.476117
90	1	0	0.022733	1.477875	-2.154387
91	1	0	0.764574	3.094704	-2.114406
92	1	0	0.890969	2.090433	-3.578739
93	1	0	3.458708	2.210536	1.152968

6a(*RS*)

Sum of electronic and zero-point Energies= -1611.442676
Sum of electronic and thermal Energies= -1611.400225
Sum of electronic and thermal Enthalpies= -1611.399280
Sum of electronic and thermal Free Energies= -1611.523149

Standard orientation:

Center Number	Atomic Number	Atomic Type	Coordinates (Angstroms) X	Y	Z
1	6	0	4.214417	-0.560519	-0.161919
2	7	0	3.690678	-1.793813	-0.284950
3	6	0	2.421436	-1.945360	0.098927
4	6	0	1.647227	-0.898391	0.616519
5	6	0	2.290454	0.349406	0.670614
6	7	0	3.557326	0.524441	0.290988
7	1	0	1.993255	-2.948267	-0.018099
8	1	0	1.754864	1.234769	1.024174
9	6	0	5.586594	-0.383494	-0.556434
10	6	0	6.750111	-0.233357	-0.891779
11	6	0	8.155505	-0.051690	-1.292684
12	6	0	9.054557	-0.200936	-0.042555
13	1	0	8.941946	-1.199031	0.408354
14	1	0	10.110219	-0.066174	-0.329105
15	1	0	8.802101	0.553943	0.718054
16	6	0	8.529485	-1.126443	-2.338695
17	1	0	7.897134	-1.041066	-3.235986
18	1	0	9.581340	-0.999439	-2.642207
19	1	0	8.407182	-2.139423	-1.925050
20	6	0	8.331836	1.359310	-1.899019
21	1	0	8.066455	2.138956	-1.168401
22	1	0	9.382284	1.506088	-2.198464
23	1	0	7.696399	1.488851	-2.788791
24	6	0	0.220755	-1.107020	1.074914
25	8	0	-0.551187	0.023247	0.665779
26	1	0	-0.160277	-2.021268	0.591403
27	5	0	-1.802725	0.005229	0.093545
28	6	0	0.615871	0.881614	3.945599
29	1	0	1.268827	1.344687	4.693243
30	1	0	-0.211922	1.485745	3.563110
31	6	0	0.821763	-0.372047	3.531535
32	6	0	1.801399	-1.460829	3.932328
33	6	0	0.062192	-1.313636	2.600314
34	6	0	0.887755	-2.487918	3.209669
35	1	0	1.961663	-1.603992	5.012866
36	1	0	2.787538	-1.349513	3.449736
37	1	0	-1.022150	-1.366178	2.791440
38	1	0	0.287737	-3.092453	3.904711
39	1	0	1.381508	-3.163522	2.495111
40	6	0	-2.625612	-1.341779	-0.159152
41	6	0	-3.902009	-1.389249	0.754045
42	6	0	-2.935115	-1.568112	-1.682022
43	1	0	-2.009615	-2.202087	0.143283
44	6	0	-5.135217	-1.976459	0.052175
45	1	0	-4.180717	-0.381613	1.108473
46	6	0	-4.341886	-2.165330	-1.897539
47	1	0	-2.968538	-0.585353	-2.187584
48	6	0	-5.365393	-1.192335	-1.260601
49	6	0	-4.793513	-3.226115	-0.827032
50	1	0	-4.480671	-2.442037	-2.957645
51	1	0	-6.382732	-1.296663	-1.661326
52	1	0	-5.976938	-2.071146	0.760409

53	6	0	-3.834256	-4.290338	-0.285414
54	1	0	-4.319254	-4.844966	0.536962
55	1	0	-3.586414	-5.026144	-1.069376
56	1	0	-2.891474	-3.884414	0.102341
57	6	0	-6.065377	-3.958162	-1.289314
58	1	0	-5.820455	-4.691435	-2.076765
59	1	0	-6.517753	-4.511543	-0.448474
60	1	0	-6.832662	-3.281453	-1.693429
61	6	0	-2.387211	1.452795	-0.254483
62	6	0	-2.921233	2.141851	1.055819
63	6	0	-1.389180	2.348647	-1.070767
64	6	0	-2.603392	3.641539	1.135141
65	1	0	-4.011169	1.984660	1.121401
66	6	0	-1.417190	3.818572	-0.603047
67	1	0	-0.362547	2.004532	-0.852914
68	6	0	-1.085816	3.834257	0.909925
69	6	0	-2.838523	4.376158	-0.226730
70	1	0	-0.802094	4.444646	-1.273585
71	1	0	-3.061332	4.087696	2.035684
72	1	0	-5.094474	-0.123156	-1.256752
73	1	0	-2.491399	1.669920	1.957581
74	1	0	-3.267400	1.302328	-0.900586
75	6	0	-1.590298	2.193077	-2.583085
76	1	0	-1.463028	1.142469	-2.894367
77	1	0	-2.593310	2.515544	-2.905467
78	1	0	-0.849875	2.792056	-3.139968
79	6	0	-1.819869	-2.368436	-2.365865
80	1	0	-0.842893	-1.869427	-2.246122
81	1	0	-1.727957	-3.385906	-1.952876
82	1	0	-2.010659	-2.462191	-3.448153
83	1	0	-3.664507	-1.972082	1.660351
84	6	0	-2.800085	5.904806	-0.060969
85	1	0	-2.743892	6.396248	-1.047511
86	1	0	-3.718221	6.261870	0.436731
87	1	0	-1.942256	6.254050	0.532685
88	6	0	-4.081481	4.039065	-1.055237
89	1	0	-4.036207	4.528932	-2.042925
90	1	0	-4.224327	2.963887	-1.221036
91	1	0	-4.984560	4.419759	-0.546324
92	1	0	-0.422582	3.033470	1.275844
93	1	0	-0.709306	4.801470	1.270026

6a(*SR*)

Sum of electronic and zero-point Energies= -1611.439932
Sum of electronic and thermal Energies= -1611.397339
Sum of electronic and thermal Enthalpies= -1611.396395
Sum of electronic and thermal Free Energies= -1611.522271

Standard orientation:

Center Number	Atomic Number	Atomic Type	Coordinates (Angstroms) X	Y	Z
1	6	0	-4.131153	0.420678	0.240351
2	7	0	-3.906649	-0.589561	1.103094
3	6	0	-2.674151	-0.690953	1.599604
4	6	0	-1.636206	0.190082	1.259076
5	6	0	-1.984699	1.196598	0.347551
6	7	0	-3.213713	1.319106	-0.160234
7	1	0	-2.497206	-1.516878	2.299448
8	1	0	-1.238616	1.922833	0.010909
9	6	0	-5.459415	0.545334	-0.297866
10	6	0	-6.586756	0.650756	-0.752581
11	6	0	-7.945357	0.780780	-1.306013
12	6	0	-8.875558	-0.247649	-0.623952
13	1	0	-8.922407	-0.077492	0.462853
14	1	0	-9.894606	-0.156988	-1.033756
15	1	0	-8.521894	-1.275791	-0.797399
16	6	0	-8.461174	2.214660	-1.042379
17	1	0	-7.808843	2.962857	-1.518556
18	1	0	-9.476695	2.324511	-1.456262
19	1	0	-8.501844	2.427565	0.037109
20	6	0	-7.893916	0.515434	-2.828980
21	1	0	-7.525070	-0.500099	-3.040792
22	1	0	-8.905468	0.613487	-3.255401
23	1	0	-7.232534	1.236650	-3.333434
24	6	0	-0.258058	0.074414	1.881242
25	8	0	0.707845	0.719951	1.062564
26	1	0	-0.011776	-0.992531	2.003107
27	5	0	1.604878	0.188249	0.162414
28	6	0	1.993693	-0.110267	4.369864
29	1	0	2.871010	0.165604	4.964588
30	1	0	1.930834	-1.149672	4.032912
31	6	0	1.045031	0.783916	4.079815
32	6	0	0.860934	2.261098	4.378445
33	6	0	-0.251073	0.736406	3.273840
34	6	0	-0.306564	2.290333	3.354211
35	1	0	1.722865	2.914317	4.166670
36	1	0	0.534431	2.446226	5.415956
37	1	0	-0.013965	2.760325	2.405076
38	1	0	-1.255054	2.725439	3.699450
39	6	0	2.603215	1.301779	-0.408039
40	6	0	3.954935	1.199566	0.391601
41	6	0	2.791479	1.330813	-1.960962
42	1	0	2.157686	2.270590	-0.130852
43	6	0	5.197684	1.452768	-0.473438
44	1	0	4.077995	0.200787	0.848497
45	6	0	4.256216	1.608755	-2.359589
46	1	0	2.579599	0.323282	-2.365170
47	6	0	5.135745	0.515918	-1.702144
48	6	0	5.008933	2.655420	-1.457495
49	1	0	4.337005	1.750054	-3.452056
50	1	0	6.107499	0.374315	-2.194641
51	1	0	6.111965	1.444446	0.145826
52	6	0	4.324248	3.930325	-0.956837

53	1	0	4.983364	4.450011	-0.239282
54	1	0	4.140506	4.628479	-1.791344
55	1	0	3.366086	3.748570	-0.454130
56	6	0	6.340119	3.075029	-2.103869
57	1	0	6.156922	3.765769	-2.944841
58	1	0	6.974272	3.603639	-1.371417
59	1	0	6.919462	2.224785	-2.493446
60	6	0	1.721378	-1.360941	-0.241610
61	6	0	2.437475	-2.229201	0.853204
62	6	0	0.421388	-2.032235	-0.808038
63	6	0	1.998395	-3.703098	0.846120
64	1	0	3.527065	-2.158802	0.697551
65	6	0	0.399724	-3.552187	-0.527603
66	1	0	-0.461383	-1.634409	-0.276548
67	6	0	0.462006	-3.753635	1.006619
68	6	0	1.796511	-4.276338	-0.598507
69	1	0	-0.431421	-4.028218	-1.077137
70	1	0	2.617538	-4.295167	1.542782
71	1	0	4.667747	-0.472045	-1.553337
72	1	0	2.254911	-1.835882	1.868183
73	1	0	2.419058	-1.349613	-1.093301
74	6	0	0.215227	-1.696860	-2.290122
75	1	0	0.150832	-0.607245	-2.447544
76	1	0	1.038498	-2.076486	-2.916491
77	1	0	-0.723307	-2.139296	-2.664089
78	1	0	3.918217	1.909246	1.234868
79	6	0	1.791081	2.283033	-2.628292
80	1	0	0.753454	2.018243	-2.363178
81	1	0	1.951865	3.329448	-2.323799
82	1	0	1.873828	2.236167	-3.727385
83	6	0	1.614393	-5.805352	-0.578107
84	1	0	1.258581	-6.159882	-1.560574
85	1	0	2.577875	-6.303240	-0.374384
86	1	0	0.895085	-6.150512	0.178788
87	6	0	2.823969	-3.974431	-1.694549
88	1	0	3.078004	-2.912632	-1.795485
89	1	0	3.760687	-4.519811	-1.484667
90	1	0	2.458619	-4.326316	-2.674368
91	1	0	0.085978	-4.730035	1.340014
92	1	0	-0.001203	-2.971364	1.629952
93	1	0	-1.058859	0.270601	3.864500

6a(*SS*)

Sum of electronic and zero-point Energies= -1611.439234
Sum of electronic and thermal Energies= -1611.396896
Sum of electronic and thermal Enthalpies= -1611.395952
Sum of electronic and thermal Free Energies= -1611.517028

Standard orientation:

Center Number	Atomic Number	Atomic Type	Coordinates (Angstroms) X	Y	Z
1	6	0	-4.168875	0.223268	0.277326
2	7	0	-3.795882	-1.058677	0.112478
3	6	0	-2.567373	-1.384000	0.520649
4	6	0	-1.685998	-0.461749	1.100335
5	6	0	-2.180877	0.847524	1.211946
6	7	0	-3.402837	1.196127	0.808746
7	1	0	-2.266700	-2.427992	0.374196
8	1	0	-1.557738	1.642574	1.634967
9	6	0	-5.492564	0.587384	-0.151931
10	6	0	-6.615231	0.896443	-0.516514
11	6	0	-7.971564	1.270746	-0.951178
12	6	0	-8.448096	0.283066	-2.040374
13	1	0	-8.470902	-0.748217	-1.655511
14	1	0	-9.464595	0.554314	-2.368685
15	1	0	-7.781988	0.310139	-2.916640
16	6	0	-8.921903	1.206037	0.267933
17	1	0	-8.597810	1.899914	1.058877
18	1	0	-9.941270	1.485907	-0.043955
19	1	0	-8.954697	0.189655	0.689903
20	6	0	-7.943734	2.708831	-1.517706
21	1	0	-7.269291	2.778381	-2.385208
22	1	0	-8.956317	2.999850	-1.841148
23	1	0	-7.602297	3.426690	-0.756009
24	6	0	-0.310406	-0.848253	1.602730
25	8	0	0.616231	0.185779	1.280563
26	1	0	-0.011136	-1.799616	1.137985
27	5	0	1.624264	0.205723	0.344191
28	6	0	2.156831	-1.124565	4.029049
29	1	0	2.917424	-1.736351	4.525987
30	1	0	2.419136	-0.087760	3.799509
31	6	0	0.952734	-1.622158	3.732168
32	6	0	0.261663	-2.947036	4.002601
33	6	0	-0.312993	-1.012073	3.139444
34	6	0	-1.089669	-2.256933	3.668089
35	1	0	0.376091	-3.360656	5.017152
36	1	0	0.535016	-3.734602	3.279470
37	1	0	-0.608522	-0.044187	3.576497
38	1	0	-1.683819	-2.022303	4.562436
39	1	0	-1.736995	-2.775496	2.945219
40	6	0	2.474777	1.563173	0.397737
41	6	0	3.938642	1.298133	0.895636
42	6	0	2.410266	2.395085	-0.930186
43	1	0	1.990713	2.182985	1.168984
44	6	0	4.993772	2.172061	0.201265
45	1	0	4.234454	0.246209	0.734095
46	6	0	3.752305	3.093132	-1.238867
47	1	0	2.258289	1.701223	-1.777749
48	6	0	4.840544	1.994300	-1.327086
49	6	0	4.521814	3.653173	0.013938
50	1	0	3.638346	3.779288	-2.096949
51	1	0	5.730325	2.295837	-1.896586
52	1	0	5.990127	2.011868	0.649778

53	6	0	3.794907	4.362268	1.160529
54	1	0	4.501926	4.555561	1.986476
55	1	0	3.408538	5.341700	0.830244
56	1	0	2.953525	3.791801	1.572637
57	6	0	5.680406	4.566770	-0.421702
58	1	0	5.292425	5.542834	-0.760348
59	1	0	6.361235	4.755719	0.426141
60	1	0	6.279884	4.145544	-1.242244
61	6	0	1.982468	-0.979675	-0.678049
62	6	0	2.761484	-2.152479	0.019344
63	6	0	0.836490	-1.481662	-1.626250
64	6	0	2.564488	-3.512254	-0.671776
65	1	0	3.833555	-1.894227	0.042997
66	6	0	1.041688	-2.960754	-2.028693
67	1	0	-0.126128	-1.466776	-1.084738
68	6	0	1.049870	-3.812067	-0.735130
69	6	0	2.537816	-3.407713	-2.235338
70	1	0	0.329321	-3.239529	-2.825121
71	1	0	3.230203	-4.272565	-0.226941
72	1	0	4.512586	1.002674	-1.682717
73	1	0	2.459282	-2.270315	1.074868
74	1	0	2.715580	-0.513774	-1.355518
75	6	0	0.656839	-0.559055	-2.837668
76	1	0	0.408700	0.468411	-2.524154
77	1	0	1.567417	-0.509327	-3.456263
78	1	0	-0.166702	-0.914791	-3.479347
79	1	0	3.972420	1.454477	1.987162
80	6	0	1.212881	3.353225	-0.933281
81	1	0	1.128022	3.875276	-1.901457
82	1	0	0.268000	2.809442	-0.765729
83	1	0	1.296832	4.118699	-0.145373
84	6	0	2.600105	-4.797401	-2.896101
85	1	0	2.365852	-4.718605	-3.971494
86	1	0	3.616340	-5.218253	-2.807635
87	1	0	1.900070	-5.523194	-2.457191
88	6	0	3.564064	-2.533298	-2.963777
89	1	0	3.310851	-2.450143	-4.034416
90	1	0	3.656804	-1.517065	-2.562803
91	1	0	4.560815	-3.004200	-2.903474
92	1	0	0.818062	-4.872282	-0.902874
93	1	0	0.433188	-3.450707	0.103985

2b(*S*)s · 3

Sum of electronic and zero-point Energies= -1611.381754
Sum of electronic and thermal Energies= -1611.339451
Sum of electronic and thermal Enthalpies= -1611.338507
Sum of electronic and thermal Free Energies= -1611.457191

Standard orientation:

Center Number	Atomic Number	Atomic Type	Coordinates (Angstroms) X	Y	Z
1	5	0	1.836626	0.033810	0.709870
2	6	0	0.753756	1.617876	3.572253
3	1	0	0.062748	1.928967	2.783411
4	6	0	1.607740	0.593595	3.433228
5	6	0	2.730716	0.102535	4.323935
6	6	0	2.016339	-0.351846	2.297442
7	6	0	3.389750	-0.483334	3.049181
8	1	0	3.286194	0.854836	4.908415
9	1	0	2.392595	-0.694350	5.009592
10	1	0	4.152796	0.197266	2.647085
11	1	0	3.821783	-1.492087	3.132106
12	6	0	2.176147	-1.218004	-0.355732
13	6	0	3.637612	-1.757678	-0.157984
14	6	0	1.102843	-2.360313	-0.391362
15	6	0	3.760643	-3.287717	-0.162114
16	6	0	1.724864	-3.772177	-0.400939
17	6	0	2.972405	-3.934567	-1.345007
18	6	0	2.700120	-3.876452	0.796198
19	1	0	0.933246	-4.537488	-0.493641
20	1	0	4.814032	-3.592899	-0.030265
21	6	0	2.397591	1.529738	0.272029
22	6	0	1.768362	2.100516	-1.049801
23	6	0	3.964828	1.619336	0.303715
24	6	0	2.814588	2.776825	-1.959523
25	6	0	4.584274	2.345431	-0.898562
26	1	0	4.421159	0.618827	0.338923
27	6	0	3.969509	1.773850	-2.196742
28	6	0	3.852486	3.690143	-1.208131
29	1	0	5.685151	2.379184	-0.810584
30	1	0	2.323554	3.218563	-2.845646
31	1	0	0.520598	-2.335450	0.555612
32	1	0	1.381890	1.251799	-1.647188
33	1	0	2.047417	2.203616	1.074069
34	6	0	3.444132	4.580058	-0.031249
35	1	0	4.346746	5.006292	0.441791
36	1	0	2.884755	4.044068	0.746885
37	1	0	2.826083	5.428897	-0.370915
38	6	0	4.637980	4.561536	-2.201579
39	1	0	4.008544	5.390930	-2.568292
40	1	0	4.992437	4.001436	-3.080002
41	1	0	5.521308	5.006558	-1.711343
42	1	0	3.703130	0.704100	-2.196804
43	1	0	4.564388	1.990519	-3.095229
44	1	0	2.450332	-3.284693	1.692470
45	1	0	2.912457	-4.910352	1.102203
46	6	0	3.040280	-3.260859	-2.717629
47	1	0	4.056312	-3.373080	-3.135630
48	1	0	2.811609	-2.187334	-2.688610
49	1	0	2.343701	-3.737545	-3.428402
50	6	0	3.326898	-5.418363	-1.535243
51	1	0	4.330770	-5.520904	-1.982369
52	1	0	2.607588	-5.901498	-2.218961

53	1	0	3.322789	-5.987278	-0.593387
54	8	0	0.221605	0.202286	0.503309
55	6	0	-0.689755	-0.240837	1.231478
56	6	0	-2.088645	-0.125786	0.876102
57	6	0	-3.080683	-0.632855	1.739953
58	6	0	-2.553950	0.480245	-0.312499
59	7	0	-4.375519	-0.557550	1.460788
60	1	0	-2.800492	-1.113686	2.684688
61	7	0	-3.844617	0.562575	-0.601966
62	1	0	-1.850161	0.904857	-1.035020
63	6	0	-4.711887	0.037348	0.295047
64	6	0	-6.103063	0.120032	-0.020963
65	6	0	-7.290710	0.189344	-0.297816
66	6	0	-8.720778	0.276198	-0.625677
67	6	0	-9.338740	1.445726	0.178857
68	1	0	-10.412527	1.520470	-0.056401
69	1	0	-8.858373	2.401683	-0.080094
70	1	0	-9.229865	1.282179	1.261906
71	6	0	-8.883319	0.533772	-2.140918
72	1	0	-8.443285	-0.285419	-2.730158
73	1	0	-8.394549	1.474837	-2.436348
74	1	0	-9.954267	0.604311	-2.389869
75	6	0	-9.409415	-1.052002	-0.234182
76	1	0	-9.300488	-1.251565	0.842846
77	1	0	-8.979053	-1.899058	-0.790241
78	1	0	-10.483765	-0.991322	-0.470826
79	1	0	2.145035	-0.760450	-1.360050
80	1	0	4.062221	-1.409095	0.795756
81	1	0	4.288734	-1.338945	-0.943997
82	1	0	4.281515	2.123714	1.233324
83	6	0	0.571071	3.026463	-0.789049
84	1	0	-0.189848	2.531268	-0.167146
85	1	0	0.868721	3.949597	-0.268690
86	1	0	0.087683	3.321224	-1.737135
87	6	0	0.088505	-2.181183	-1.530136
88	1	0	-0.727787	-2.920410	-1.454501
89	1	0	-0.362661	-1.176927	-1.515400
90	1	0	0.560081	-2.306654	-2.516375
91	1	0	1.492158	-1.315085	2.484894
92	1	0	-0.447473	-0.724978	2.189949
93	1	0	0.732348	2.206484	4.496537

2b(*R*)s · 3

Sum of electronic and zero-point Energies= -1611.376598
Sum of electronic and thermal Energies= -1611.334438
Sum of electronic and thermal Enthalpies= -1611.333494
Sum of electronic and thermal Free Energies= -1611.451932

Standard orientation:

Center Number	Atomic Number	Atomic Type	Coordinates (Angstroms) X	Y	Z
1	5	0	1.792321	0.083489	0.418478
2	6	0	-0.104311	1.545229	3.000412
3	1	0	-0.725089	1.688047	3.892345
4	1	0	-0.398320	2.099534	2.106260
5	6	0	0.986628	0.759031	3.055320
6	6	0	1.478445	-0.138851	4.169346
7	6	0	2.014492	0.197053	2.066216
8	6	0	2.171937	-0.991635	3.080528
9	1	0	2.216165	0.380487	4.805266
10	1	0	0.726283	-0.612156	4.822725
11	1	0	2.924224	0.821563	2.176457
12	1	0	3.190131	-1.346761	3.298259
13	1	0	1.553444	-1.858992	2.803435
14	6	0	2.289021	-1.350175	-0.319261
15	6	0	3.689582	-1.838259	0.179202
16	6	0	1.248250	-2.513415	-0.440446
17	6	0	3.871231	-3.365962	0.168887
18	6	0	1.909583	-3.910496	-0.376914
19	6	0	3.290717	-4.024657	-1.125944
20	6	0	2.703177	-4.014827	0.946236
21	1	0	1.163238	-4.694992	-0.597338
22	1	0	4.903471	-3.637529	0.453451
23	6	0	2.307523	1.419867	-0.422062
24	6	0	3.866792	1.537719	-0.601903
25	6	0	1.657609	2.749326	0.096094
26	6	0	4.374612	2.986075	-0.427484
27	6	0	2.603197	3.956373	0.167742
28	1	0	1.264795	2.612831	1.114768
29	6	0	3.874527	3.525831	0.933630
30	6	0	3.485361	4.089725	-1.113299
31	1	0	2.065422	4.859428	0.509086
32	1	0	5.459195	3.033517	-0.633600
33	1	0	0.561860	-2.498282	0.432527
34	1	0	4.362556	0.968435	0.208122
35	1	0	1.909015	1.263646	-1.442489
36	6	0	2.821668	3.870768	-2.475456
37	1	0	2.125209	4.702275	-2.684927
38	1	0	2.249179	2.936007	-2.535476
39	1	0	3.569798	3.864291	-3.286634
40	6	0	4.227523	5.435037	-1.169932
41	1	0	5.000337	5.416082	-1.957914
42	1	0	4.725757	5.693343	-0.223441
43	1	0	3.526805	6.253152	-1.411638
44	1	0	3.744125	2.788345	1.743383
45	1	0	4.465187	4.369734	1.317176
46	1	0	2.305455	-3.460861	1.809429
47	1	0	2.905754	-5.049562	1.255487
48	6	0	3.546604	-3.358893	-2.480998
49	1	0	4.615400	-3.452030	-2.742585
50	1	0	3.292869	-2.291772	-2.503803
51	1	0	2.974088	-3.860228	-3.280130
52	6	0	3.714855	-5.497753	-1.262584

53	1	0	4.777512	-5.565817	-1.552723
54	1	0	3.123443	-5.995360	-2.050475
55	1	0	3.585663	-6.076204	-0.335980
56	8	0	0.202495	0.147815	0.171726
57	6	0	-0.665660	-0.399554	0.888896
58	1	0	-0.374737	-1.035931	1.734488
59	6	0	-2.080090	-0.262954	0.597323
60	6	0	-3.045974	-0.915539	1.388332
61	6	0	-2.577987	0.510381	-0.473695
62	7	0	-4.347526	-0.816985	1.149259
63	1	0	-2.741143	-1.533099	2.241336
64	7	0	-3.875827	0.617111	-0.723314
65	1	0	-1.892314	1.050582	-1.134613
66	6	0	-4.716987	-0.051329	0.099161
67	6	0	-6.117435	0.064295	-0.165491
68	6	0	-7.314052	0.163051	-0.389070
69	6	0	-8.753424	0.288898	-0.660184
70	6	0	-9.193405	1.735160	-0.327566
71	1	0	-10.271003	1.843643	-0.530218
72	1	0	-8.647585	2.466258	-0.943348
73	1	0	-9.013966	1.969295	0.732991
74	6	0	-9.009105	-0.005675	-2.157094
75	1	0	-8.696375	-1.028434	-2.417958
76	1	0	-8.459942	0.700374	-2.798614
77	1	0	-10.085439	0.093498	-2.370792
78	6	0	-9.530879	-0.715991	0.219410
79	1	0	-9.355738	-0.522711	1.288934
80	1	0	-9.226637	-1.751000	-0.000093
81	1	0	-10.610651	-0.622089	0.021274
82	1	0	2.437519	-1.031867	-1.363848
83	1	0	3.891643	-1.494196	1.206394
84	1	0	4.473126	-1.379313	-0.445905
85	1	0	0.783407	2.998176	-0.531526
86	6	0	4.375218	0.952126	-1.929144
87	1	0	5.477884	0.984411	-1.971558
88	1	0	4.071044	-0.096275	-2.063152
89	1	0	3.994025	1.515327	-2.794579
90	6	0	0.372252	-2.373341	-1.694991
91	1	0	-0.434076	-3.127055	-1.702458
92	1	0	-0.094167	-1.378019	-1.760595
93	1	0	0.965084	-2.512448	-2.612301

2b(*S*)r · 3

Sum of electronic and zero-point Energies= -1611.375023
Sum of electronic and thermal Energies= -1611.332911
Sum of electronic and thermal Enthalpies= -1611.331967
Sum of electronic and thermal Free Energies= -1611.449623

Standard orientation:

Center Number	Atomic Number	Atomic Type	Coordinates (Angstroms) X	Y	Z
1	5	0	-1.744438	0.080697	0.356561
2	6	0	0.362949	-0.820932	3.159681
3	1	0	0.743325	-1.557253	3.876577
4	1	0	1.090442	-0.117135	2.745664
5	6	0	-0.956095	-0.716333	2.895521
6	6	0	-2.180289	-1.364559	3.519767
7	6	0	-1.775530	0.213058	2.045158
8	6	0	-3.038623	-0.264778	2.836497
9	1	0	-2.184600	-1.372650	4.622089
10	1	0	-2.392449	-2.389170	3.173136
11	1	0	-1.508707	1.268936	2.222841
12	1	0	-3.406340	0.488734	3.549879
13	1	0	-3.891248	-0.619952	2.241670
14	6	0	-2.365286	-1.255375	-0.428413
15	6	0	-3.900497	-1.188431	-0.797986
16	6	0	-1.959665	-2.660981	0.150866
17	6	0	-4.596837	-2.565467	-0.707533
18	6	0	-3.054238	-3.737488	0.109017
19	1	0	-1.090962	-3.050116	-0.409541
20	6	0	-3.767691	-3.774687	-1.280106
21	6	0	-4.356646	-3.157396	0.700784
22	1	0	-2.673233	-4.694041	0.510061
23	1	0	-5.640914	-2.478162	-1.057637
24	6	0	-2.171698	1.537723	-0.318520
25	6	0	-3.576180	2.029424	0.176178
26	6	0	-1.072011	2.654000	-0.221466
27	6	0	-3.667480	3.537126	0.460527
28	1	0	-4.342967	1.759101	-0.569211
29	6	0	-1.658188	4.048482	0.089415
30	1	0	-0.412603	2.426547	0.639131
31	6	0	-2.497806	3.943187	1.384987
32	6	0	-2.996666	4.389086	-0.665079
33	1	0	-0.862738	4.814761	0.049933
34	1	0	-4.693509	3.815798	0.761056
35	1	0	-1.629405	-2.575181	1.198984
36	1	0	-3.871277	1.513593	1.105159
37	1	0	-2.276255	1.351772	-1.401464
38	6	0	-0.174238	2.687630	-1.466848
39	1	0	0.246741	1.693608	-1.683165
40	1	0	-0.730871	3.008494	-2.361163
41	1	0	0.666666	3.390410	-1.332404
42	6	0	-3.229461	3.990680	-2.124953
43	1	0	-2.598018	4.589579	-2.803529
44	1	0	-3.029540	2.930385	-2.326202
45	1	0	-4.280141	4.189839	-2.401117
46	6	0	-3.334785	5.883908	-0.537427
47	1	0	-4.378143	6.071192	-0.845390
48	1	0	-3.216715	6.268187	0.486768
49	1	0	-2.681883	6.483850	-1.194894
50	1	0	-2.168095	3.203000	2.132888
51	1	0	-2.651969	4.907232	1.889689
52	1	0	-4.259290	-2.443066	1.531817

53	1	0	-5.087401	-3.928073	0.982908
54	6	0	-2.912744	-3.651168	-2.544440
55	1	0	-3.546559	-3.563813	-3.443249
56	1	0	-2.230007	-2.791626	-2.530168
57	1	0	-2.300776	-4.562396	-2.668467
58	6	0	-4.652965	-5.021272	-1.445085
59	1	0	-5.308186	-4.912719	-2.326539
60	1	0	-4.030055	-5.918815	-1.602945
61	1	0	-5.297851	-5.213309	-0.574733
62	8	0	-0.137062	0.031307	0.146689
63	6	0	0.664866	-0.918805	0.298267
64	1	0	0.300427	-1.941190	0.453298
65	6	0	2.100383	-0.730756	0.177084
66	6	0	2.968834	-1.838856	0.236026
67	6	0	2.716762	0.525142	-0.008875
68	7	0	4.285780	-1.721622	0.120727
69	1	0	2.569351	-2.849600	0.381395
70	7	0	4.031897	0.656779	-0.123278
71	1	0	2.115716	1.437893	-0.060140
72	6	0	4.771693	-0.473457	-0.055513
73	6	0	6.189800	-0.333197	-0.179857
74	6	0	7.400318	-0.211272	-0.286014
75	6	0	8.856880	-0.055527	-0.412313
76	6	0	9.378864	0.727787	0.816192
77	1	0	10.469929	0.855522	0.730742
78	1	0	8.915047	1.724231	0.876299
79	1	0	9.164556	0.186677	1.750614
80	6	0	9.164069	0.734141	-1.706706
81	1	0	8.794535	0.197775	-2.594210
82	1	0	8.696540	1.730366	-1.682467
83	1	0	10.253761	0.862623	-1.807240
84	6	0	9.519663	-1.450166	-0.471380
85	1	0	9.306486	-2.027755	0.441254
86	1	0	9.155640	-2.023962	-1.337542
87	1	0	10.611693	-1.336831	-0.564015
88	1	0	-1.853499	-1.186155	-1.408721
89	1	0	-4.421415	-0.562340	-0.052118
90	6	0	-4.169117	-0.556209	-2.173148
91	1	0	-3.751385	0.457438	-2.253423
92	1	0	-3.734280	-1.155220	-2.988131
93	1	0	-5.254689	-0.482620	-2.358316

2b(*R*)r · 3

Sum of electronic and zero-point Energies= -1611.371674
Sum of electronic and thermal Energies= -1611.329250
Sum of electronic and thermal Enthalpies= -1611.328306
Sum of electronic and thermal Free Energies= -1611.446477

Standard orientation:

Center Number	Atomic Number	Atomic Type	Coordinates (Angstroms) X	Y	Z
1	5	0	-1.797743	-0.075928	0.469628
2	6	0	-0.557974	1.895920	3.238805
3	1	0	0.399272	1.731596	2.734961
4	1	0	-0.597559	2.707243	3.974537
5	6	0	-1.635897	1.142968	2.979322
6	6	0	-3.026586	1.131017	3.589878
7	6	0	-1.916799	-0.086938	2.121395
8	6	0	-3.290905	-0.220397	2.875501
9	1	0	-3.668928	1.954664	3.232847
10	1	0	-3.068380	1.124779	4.691965
11	1	0	-4.201692	-0.260352	2.261061
12	1	0	-3.316928	-1.071625	3.573183
13	6	0	-2.127222	-1.533491	-0.251498
14	6	0	-3.666135	-1.829427	-0.439986
15	6	0	-1.340426	-2.743433	0.369174
16	6	0	-4.023555	-3.295710	-0.117938
17	6	0	-2.170019	-4.016407	0.580286
18	1	0	-0.472560	-2.993332	-0.268798
19	6	0	-3.019655	-4.368164	-0.682267
20	6	0	-3.485981	-3.635013	1.293862
21	1	0	-1.550382	-4.821899	1.013711
22	1	0	-5.094998	-3.475207	-0.318494
23	6	0	-2.312876	1.238003	-0.415979
24	6	0	-3.759175	1.687206	-0.005878
25	6	0	-1.322016	2.456759	-0.566588
26	6	0	-4.001144	3.204026	-0.046167
27	1	0	-4.495510	1.183654	-0.655221
28	6	0	-2.050551	3.818628	-0.544819
29	1	0	-0.645208	2.479755	0.305207
30	6	0	-2.880941	3.903201	0.757480
31	6	0	-3.412264	3.863195	-1.334083
32	1	0	-1.335165	4.634151	-0.756029
33	1	0	-5.051843	3.436415	0.205321
34	1	0	-0.914206	-2.470249	1.350727
35	1	0	-3.997246	1.359518	1.016553
36	1	0	-2.406678	0.849795	-1.446144
37	6	0	-0.444196	2.349141	-1.823714
38	1	0	0.103647	1.395259	-1.880409
39	1	0	-1.042591	2.423355	-2.744868
40	1	0	0.306626	3.157757	-1.848878
41	6	0	-3.606956	3.152759	-2.676842
42	1	0	-3.032677	3.651518	-3.476544
43	1	0	-3.314087	2.094951	-2.660492
44	1	0	-4.671413	3.198025	-2.968191
45	6	0	-3.891783	5.313777	-1.512069
46	1	0	-4.949094	5.334853	-1.828682
47	1	0	-3.807907	5.912913	-0.593195
48	1	0	-3.300930	5.820931	-2.294542
49	1	0	-2.475974	3.367307	1.629143
50	1	0	-3.131682	4.931762	1.052914
51	1	0	-3.440310	-2.808475	2.019182
52	1	0	-3.991964	-4.490899	1.762030

53	6	0	-2.367935	-4.224073	-2.060093
54	1	0	-3.105747	-4.376344	-2.866140
55	1	0	-1.894676	-3.245711	-2.218467
56	1	0	-1.588560	-4.996963	-2.184789
57	6	0	-3.621375	-5.780420	-0.599130
58	1	0	-4.383529	-5.920984	-1.384965
59	1	0	-2.839204	-6.543842	-0.754016
60	1	0	-4.101454	-5.987884	0.368885
61	8	0	-0.095699	0.031570	0.403522
62	6	0	0.671858	-0.492212	-0.418999
63	1	0	0.266713	-1.074042	-1.264238
64	6	0	2.115018	-0.376560	-0.323042
65	6	0	2.943616	-0.978798	-1.290218
66	6	0	2.775158	0.319971	0.712830
67	7	0	4.267331	-0.900235	-1.242400
68	1	0	2.506766	-1.539468	-2.125186
69	7	0	4.095803	0.406004	0.772265
70	1	0	2.202449	0.811684	1.505533
71	6	0	4.797211	-0.207612	-0.210451
72	6	0	6.221433	-0.114736	-0.148640
73	6	0	7.439414	-0.037518	-0.097194
74	6	0	8.904242	0.057910	-0.028860
75	6	0	9.383173	-0.607037	1.284116
76	1	0	10.480804	-0.536427	1.348003
77	1	0	8.949576	-0.105537	2.162745
78	1	0	9.101158	-1.670654	1.314854
79	6	0	9.305537	1.552426	-0.036135
80	1	0	8.967330	2.048472	-0.958810
81	1	0	8.870685	2.083022	0.824593
82	1	0	10.402711	1.634956	0.020868
83	6	0	9.523459	-0.664396	-1.246428
84	1	0	9.242753	-1.728792	-1.258965
85	1	0	9.188655	-0.206296	-2.189789
86	1	0	10.621894	-0.595245	-1.197244
87	1	0	-1.751825	-1.428639	-1.289540
88	1	0	-4.234235	-1.233632	0.296258
89	6	0	-4.193174	-1.424653	-1.825275
90	1	0	-3.994395	-0.364594	-2.042546
91	1	0	-3.732473	-2.017173	-2.630503
92	1	0	-5.285001	-1.575245	-1.884422
93	1	0	-1.239135	-0.901606	2.437515

TS8

Sum of electronic and zero-point Energies= -1611.378453
Sum of electronic and thermal Energies= -1611.337159
Sum of electronic and thermal Enthalpies= -1611.336215
Sum of electronic and thermal Free Energies= -1611.452148

Standard orientation:

Center Number	Atomic Number	Atomic Type	X	Y	Z
1	5	0	-1.614678	-0.049925	0.579521
2	6	0	0.420922	-1.829482	2.372181
3	1	0	-0.206294	-2.682521	2.102143
4	1	0	1.443834	-2.060707	2.683886
5	6	0	-0.149140	-0.630006	2.735632
6	6	0	0.479185	0.628570	3.276205
7	6	0	-1.389351	0.035015	2.370827
8	6	0	-0.874189	1.343688	3.018018
9	1	0	1.346538	0.999031	2.702509
10	1	0	0.780240	0.552873	4.334727
11	1	0	-0.811866	2.212159	2.351367
12	1	0	-1.401816	1.641553	3.937379
13	6	0	-2.384833	-1.482972	0.244270
14	6	0	-2.640705	-1.744837	-1.290269
15	6	0	-3.670149	-1.707082	1.114812
16	6	0	-3.992068	-2.447975	-1.548549
17	6	0	-4.837136	-2.380822	0.379986
18	1	0	-3.402469	-2.304806	2.004197
19	6	0	-4.371340	-3.573208	-0.518265
20	6	0	-5.111181	-1.585980	-0.916952
21	1	0	-5.679595	-2.566605	1.069815
22	1	0	-4.083679	-2.708636	-2.618232
23	6	0	-2.335932	1.306984	-0.027067
24	6	0	-3.541412	1.853763	0.805618
25	6	0	-1.392493	2.448583	-0.541501
26	6	0	-3.801469	3.360869	0.625485
27	1	0	-4.451398	1.292739	0.532228
28	6	0	-2.079586	3.835055	-0.495935
29	1	0	-0.519106	2.532094	0.132277
30	6	0	-2.501860	4.125534	0.964172
31	6	0	-3.614333	3.838104	-0.854554
32	1	0	-1.454377	4.587056	-1.010530
33	1	0	-4.733055	3.660739	1.138149
34	1	0	-4.055981	-0.749288	1.504113
35	1	0	-3.398535	1.680580	1.887028
36	1	0	-2.796316	0.919929	-0.952020
37	6	0	-0.840127	2.148069	-1.942156
38	1	0	-0.321532	1.177971	-1.968282
39	1	0	-1.642956	2.120101	-2.696766
40	1	0	-0.118253	2.922493	-2.255023
41	6	0	-4.197309	3.006421	-2.002073
42	1	0	-3.864200	3.400361	-2.977632
43	1	0	-3.933589	1.942791	-1.963173
44	1	0	-5.299432	3.075693	-1.986194
45	6	0	-4.122252	5.277745	-1.057777
46	1	0	-5.225805	5.299817	-1.054003
47	1	0	-3.773483	5.978473	-0.285216
48	1	0	-3.786585	5.666777	-2.034590
49	1	0	-1.867644	3.707410	1.760191
50	1	0	-2.662580	5.192465	1.171464
51	1	0	-4.936901	-0.497954	-0.881780
52	1	0	-6.106144	-1.771003	-1.345056

53	6	0	-3.327641	-4.553248	0.024231
54	1	0	-2.991212	-5.245525	-0.766602
55	1	0	-2.439525	-4.060476	0.440174
56	1	0	-3.772686	-5.168975	0.825712
57	6	0	-5.560456	-4.405821	-1.026382
58	1	0	-5.234319	-5.094812	-1.824579
59	1	0	-5.979762	-5.018293	-0.209432
60	1	0	-6.376637	-3.790363	-1.432883
61	8	0	-0.154635	-0.080191	0.142582
62	6	0	0.639257	-1.091624	0.290533
63	1	0	0.281017	-2.091824	0.028973
64	6	0	2.081862	-0.856755	0.141548
65	6	0	2.991601	-1.924825	0.033740
66	6	0	2.647342	0.429590	0.060765
67	7	0	4.300085	-1.744804	-0.112203
68	1	0	2.636780	-2.961965	0.068407
69	7	0	3.955899	0.627286	-0.083082
70	1	0	2.009594	1.317845	0.102061
71	6	0	4.737653	-0.468556	-0.156836
72	6	0	6.150311	-0.262761	-0.300840
73	6	0	7.352054	-0.088142	-0.424913
74	6	0	8.801272	0.123788	-0.570392
75	6	0	9.417565	-1.076481	-1.324693
76	1	0	10.503825	-0.926838	-1.434225
77	1	0	9.249509	-2.016159	-0.776212
78	1	0	8.978860	-1.178991	-2.329289
79	6	0	9.430635	0.237803	0.838613
80	1	0	9.002490	1.085510	1.395438
81	1	0	9.263898	-0.681082	1.421609
82	1	0	10.516923	0.397159	0.743572
83	6	0	9.046426	1.429056	-1.361496
84	1	0	8.602822	1.370207	-2.367431
85	1	0	8.609170	2.294701	-0.840497
86	1	0	10.129943	1.598552	-1.469463
87	1	0	-1.697790	-2.290213	0.552728
88	1	0	-2.738097	-0.769786	-1.802104
89	6	0	-1.485663	-2.471468	-1.996453
90	1	0	-0.571099	-1.858795	-2.019849
91	1	0	-1.240038	-3.434699	-1.519675
92	1	0	-1.752292	-2.683312	-3.046129
93	1	0	-2.362564	-0.379216	2.668242

TS9

Sum of electronic and zero-point Energies= -1611.375508
Sum of electronic and thermal Energies= -1611.334390
Sum of electronic and thermal Enthalpies= -1611.333446
Sum of electronic and thermal Free Energies= -1611.449422

Standard orientation:

Center Number	Atomic Number	Atomic Type	Coordinates (Angstroms) X	Y	Z
1	5	0	1.882402	0.055025	0.486778
2	6	0	-0.403227	0.057234	3.038252
3	1	0	-0.816132	0.966452	2.595852
4	1	0	-1.062525	-0.499178	3.713538
5	6	0	0.951525	-0.153478	3.042291
6	6	0	1.891995	-1.017382	3.858118
7	6	0	2.022080	0.398950	2.210748
8	6	0	3.069101	-0.338994	3.095998
9	1	0	1.789802	-2.108459	3.749075
10	1	0	1.846781	-0.782406	4.935063
11	1	0	3.763475	-1.015265	2.579008
12	1	0	3.654775	0.338293	3.736385
13	6	0	2.435232	1.406471	-0.303148
14	6	0	3.879963	1.876930	0.106867
15	6	0	1.396235	2.575088	-0.251796
16	6	0	4.017459	3.419088	0.103807
17	6	0	2.005108	3.982797	-0.159180
18	1	0	0.738646	2.506654	-1.135804
19	6	0	3.267043	4.154448	-1.068042
20	6	0	2.954882	4.011824	1.059040
21	1	0	1.220960	4.757970	-0.230963
22	1	0	5.072043	3.704907	0.268045
23	6	0	2.509189	-1.397298	-0.031021
24	6	0	2.279279	-2.637427	0.905317
25	6	0	2.150940	-1.720183	-1.541942
26	6	0	1.929209	-3.951028	0.191868
27	1	0	3.180048	-2.802090	1.519878
28	6	0	1.875803	-3.223573	-1.779782
29	1	0	1.196519	-1.216544	-1.787243
30	6	0	0.775965	-3.696427	-0.804696
31	6	0	2.857634	-4.204591	-1.039104
32	1	0	1.718272	-3.406631	-2.857541
33	1	0	1.820735	-4.773433	0.921846
34	1	0	0.725255	2.462423	0.620296
35	1	0	1.471337	-2.447809	1.630251
36	1	0	3.599199	-1.226381	-0.022205
37	6	0	3.180606	-1.221201	-2.567267
38	1	0	3.302668	-0.130340	-2.540510
39	1	0	4.173473	-1.669855	-2.409864
40	1	0	2.854768	-1.488394	-3.587530
41	6	0	4.352786	-3.905445	-0.901405
42	1	0	4.865175	-4.001492	-1.873692
43	1	0	4.565090	-2.903629	-0.506629
44	1	0	4.816368	-4.638505	-0.217641
45	6	0	2.722444	-5.636465	-1.583385
46	1	0	3.212797	-6.355327	-0.904308
47	1	0	1.677070	-5.956257	-1.707771
48	1	0	3.214530	-5.720796	-2.567615
49	1	0	0.008511	-2.956035	-0.528924
50	1	0	0.268375	-4.616087	-1.127333
51	1	0	2.670422	3.397700	1.930505
52	1	0	3.193382	5.025235	1.410850

53	6	0	3.243972	3.598295	-2.494756
54	1	0	4.240698	3.676482	-2.961933
55	1	0	2.925534	2.550305	-2.554460
56	1	0	2.549505	4.193057	-3.114282
57	6	0	3.707889	5.625453	-1.164685
58	1	0	4.711111	5.696491	-1.619430
59	1	0	3.012322	6.194613	-1.805557
60	1	0	3.750069	6.131900	-0.188991
61	8	0	0.342800	0.084101	0.309697
62	6	0	-0.518820	-0.691133	0.848901
63	1	0	-0.236523	-1.694591	1.179724
64	6	0	-1.943251	-0.459952	0.587203
65	6	0	-2.918786	-1.393005	0.982876
66	6	0	-2.424761	0.673734	-0.094996
67	7	0	-4.215445	-1.217449	0.751238
68	1	0	-2.629239	-2.311885	1.506717
69	7	0	-3.719081	0.864236	-0.332864
70	1	0	-1.730414	1.437519	-0.458804
71	6	0	-4.570619	-0.086030	0.106554
72	6	0	-5.968747	0.123143	-0.133095
73	6	0	-7.159251	0.305723	-0.332272
74	6	0	-8.593200	0.525981	-0.578380
75	6	0	-8.883760	2.044171	-0.556958
76	1	0	-9.955418	2.218304	-0.745458
77	1	0	-8.628180	2.481375	0.420632
78	1	0	-8.304504	2.568130	-1.332746
79	6	0	-9.413884	-0.186355	0.520905
80	1	0	-9.216725	-1.269471	0.521797
81	1	0	-9.167807	0.211309	1.517653
82	1	0	-10.489413	-0.028510	0.340257
83	6	0	-8.953442	-0.058488	-1.965266
84	1	0	-8.376228	0.432886	-2.763528
85	1	0	-8.749933	-1.139706	-2.003751
86	1	0	-10.025821	0.101028	-2.162580
87	1	0	2.507606	1.129885	-1.366413
88	1	0	4.063066	1.585513	1.158591
89	6	0	5.007554	1.233740	-0.712809
90	1	0	4.997733	0.136349	-0.635909
91	1	0	4.944485	1.493596	-1.781313
92	1	0	5.989635	1.579467	-0.346196
93	1	0	2.071028	1.498095	2.182315

TS10

Sum of electronic and zero-point Energies= -1611.373370
Sum of electronic and thermal Energies= -1611.332197
Sum of electronic and thermal Enthalpies= -1611.331253
Sum of electronic and thermal Free Energies= -1611.447428

Standard orientation:

Center Number	Atomic Number	Atomic Type	Coordinates (Angstroms) X	Y	Z
1	5	0	-1.730804	0.074420	0.356368
2	6	0	0.569753	-0.419116	2.861355
3	1	0	1.173132	-1.121533	3.446754
4	1	0	1.062884	0.505886	2.555491
5	6	0	-0.798394	-0.506478	2.845488
6	6	0	-1.815620	-1.385049	3.544442
7	6	0	-1.811371	0.249953	2.110131
8	6	0	-2.919170	-0.480528	2.922063
9	1	0	-1.737358	-1.333689	4.643403
10	1	0	-1.830208	-2.449353	3.259733
11	1	0	-1.745821	1.344919	2.197440
12	1	0	-3.409618	0.162502	3.668680
13	1	0	-3.695990	-0.993826	2.341328
14	6	0	-2.376106	-1.275431	-0.388467
15	6	0	-3.918490	-1.262274	-0.723988
16	6	0	-1.907212	-2.669988	0.161572
17	6	0	-4.543730	-2.677211	-0.693045
18	6	0	-2.937953	-3.805588	0.060556
19	1	0	-0.996291	-2.983868	-0.377112
20	6	0	-3.656511	-3.815093	-1.326231
21	6	0	-4.267354	-3.327164	0.682211
22	1	0	-2.502919	-4.757659	0.414658
23	1	0	-5.592662	-2.627604	-1.035603
24	6	0	-2.216138	1.517451	-0.302871
25	6	0	-3.615107	1.986928	0.223203
26	6	0	-1.136415	2.653810	-0.248952
27	6	0	-3.733928	3.500466	0.467190
28	1	0	-4.398636	1.677990	-0.488469
29	6	0	-1.744763	4.045152	0.034658
30	1	0	-0.460003	2.458645	0.606664
31	6	0	-2.553244	3.961855	1.350961
32	6	0	-3.107803	4.334992	-0.697622
33	1	0	-0.967186	4.826328	-0.045522
34	1	0	-4.758662	3.765666	0.783710
35	1	0	-1.621571	-2.599770	1.223670
36	1	0	-3.868902	1.489088	1.175541
37	1	0	-2.345887	1.315624	-1.380224
38	6	0	-0.258183	2.668171	-1.508215
39	1	0	0.170590	1.673543	-1.703673
40	1	0	-0.833413	2.960099	-2.400882
41	1	0	0.575474	3.384576	-1.404368
42	6	0	-3.364352	3.894419	-2.141445
43	1	0	-2.760058	4.488687	-2.848148
44	1	0	-3.147480	2.833881	-2.322090
45	1	0	-4.424547	4.066098	-2.398925
46	6	0	-3.476748	5.825265	-0.603194
47	1	0	-4.530726	5.980568	-0.892076
48	1	0	-3.344241	6.241886	0.406469
49	1	0	-2.852738	6.420099	-1.292503
50	1	0	-2.188814	3.255814	2.116207
51	1	0	-2.719052	4.937024	1.829743
52	1	0	-4.206324	-2.654335	1.550847

53	1	0	-4.955845	-4.148421	0.924785
54	6	0	-2.817138	-3.597686	-2.588759
55	1	0	-3.460647	-3.501920	-3.479773
56	1	0	-2.173065	-2.710216	-2.542463
57	1	0	-2.165979	-4.474454	-2.754199
58	6	0	-4.479991	-5.096060	-1.544446
59	1	0	-5.141810	-4.981039	-2.420065
60	1	0	-3.813693	-5.953512	-1.742594
61	1	0	-5.112601	-5.358690	-0.683694
62	8	0	-0.182949	0.031193	0.179097
63	6	0	0.631428	-0.860651	0.602236
64	1	0	0.291452	-1.886753	0.767079
65	6	0	2.072620	-0.682656	0.390086
66	6	0	2.964359	-1.754884	0.575410
67	6	0	2.657225	0.529542	-0.023020
68	7	0	4.275482	-1.640488	0.390105
69	1	0	2.591775	-2.738886	0.884797
70	7	0	3.966965	0.660590	-0.211829
71	1	0	2.036479	1.411323	-0.206244
72	6	0	4.731788	-0.429354	0.008095
73	6	0	6.146272	-0.287837	-0.180161
74	6	0	7.350328	-0.162841	-0.337995
75	6	0	8.802508	-0.013628	-0.523395
76	6	0	9.475467	0.063576	0.867916
77	1	0	10.564803	0.173223	0.742696
78	1	0	9.103013	0.927395	1.439830
79	1	0	9.282508	-0.849586	1.451731
80	6	0	9.085409	1.281731	-1.317404
81	1	0	8.609671	1.248373	-2.309693
82	1	0	8.705000	2.164675	-0.781024
83	1	0	10.171926	1.401048	-1.457207
84	6	0	9.340657	-1.238807	-1.298322
85	1	0	9.144949	-2.171763	-0.747629
86	1	0	8.869972	-1.316563	-2.290549
87	1	0	10.429142	-1.138204	-1.437538
88	1	0	-1.891080	-1.182103	-1.379382
89	1	0	-4.455849	-0.695053	0.057468
90	6	0	-4.245056	-0.594168	-2.069986
91	1	0	-3.869016	0.436420	-2.130388
92	1	0	-3.808836	-1.151639	-2.913163
93	1	0	-5.336968	-0.558199	-2.226394

TS11

Sum of electronic and zero-point Energies= -1611.350530
Sum of electronic and thermal Energies= -1611.309136
Sum of electronic and thermal Enthalpies= -1611.308192
Sum of electronic and thermal Free Energies= -1611.425991

Standard orientation:

Center Number	Atomic Number	Atomic Type	Coordinates (Angstroms) X	Y	Z
1	5	0	1.770889	0.018557	0.618157
2	6	0	-0.467221	0.774881	2.739601
3	1	0	-0.472784	1.555494	1.975977
4	6	0	0.760813	0.218433	3.101834
5	6	0	1.511588	-0.038110	4.374013
6	6	0	1.815105	-0.332797	2.236172
7	6	0	2.776373	-0.178948	3.467052
8	1	0	1.471740	0.760259	5.133375
9	1	0	1.227629	-0.990736	4.853523
10	1	0	3.346736	0.759062	3.435352
11	1	0	3.456236	-1.014437	3.682702
12	6	0	2.458257	-1.128673	-0.358419
13	6	0	3.904915	-1.525950	0.100569
14	6	0	1.541164	-2.378989	-0.613764
15	6	0	4.218704	-3.027071	0.012781
16	6	0	2.324897	-3.708723	-0.612947
17	6	0	3.723987	-3.657879	-1.329445
18	6	0	3.092529	-3.808117	0.727650
19	1	0	1.660111	-4.546905	-0.890812
20	1	0	5.264038	-3.228011	0.309049
21	6	0	2.209342	1.596051	0.338764
22	6	0	1.780952	2.138007	-1.071201
23	6	0	3.719781	1.856864	0.677456
24	6	0	2.903807	2.939203	-1.762523
25	6	0	4.477686	2.664395	-0.385152
26	1	0	4.263362	0.908953	0.812226
27	6	0	4.180904	2.064006	-1.778833
28	6	0	3.681019	3.934738	-0.824182
29	1	0	5.533095	2.808717	-0.091160
30	1	0	2.546243	3.349772	-2.724282
31	1	0	0.834418	-2.481119	0.233568
32	1	0	1.601481	1.270722	-1.735174
33	1	0	1.632135	2.195997	1.067106
34	6	0	2.966093	4.749974	0.256549
35	1	0	3.712135	5.253827	0.896833
36	1	0	2.326934	4.139925	0.908787
37	1	0	2.338649	5.539580	-0.191361
38	6	0	4.544820	4.904813	-1.646023
39	1	0	3.913191	5.674035	-2.123387
40	1	0	5.115303	4.403797	-2.442515
41	1	0	5.268314	5.425901	-0.994986
42	1	0	4.031753	0.973488	-1.834295
43	1	0	4.909675	2.361626	-2.545975
44	1	0	2.625125	-3.323840	1.602760
45	1	0	3.374300	-4.834215	1.003098
46	6	0	3.934141	-2.879108	-2.630429
47	1	0	5.009932	-2.851994	-2.879095
48	1	0	3.578246	-1.842237	-2.579758
49	1	0	3.419923	-3.373272	-3.472592
50	6	0	4.285659	-5.073622	-1.541719
51	1	0	5.355060	-5.028279	-1.811518
52	1	0	3.755826	-5.577847	-2.368415

53	1	0	4.194640	-5.713386	-0.651016
54	8	0	0.177213	-0.017429	0.340439
55	6	0	-0.702501	-0.448465	1.205021
56	6	0	-2.138077	-0.328088	0.836005
57	6	0	-3.138370	-0.962710	1.591171
58	6	0	-2.587683	0.377184	-0.291790
59	7	0	-4.430663	-0.889736	1.284777
60	1	0	-2.875140	-1.552614	2.477784
61	7	0	-3.878041	0.462115	-0.614703
62	1	0	-1.873576	0.871769	-0.956077
63	6	0	-4.754154	-0.167148	0.192103
64	6	0	-6.147148	-0.065471	-0.140995
65	6	0	-7.330545	0.024861	-0.425628
66	6	0	-8.758477	0.129419	-0.767493
67	6	0	-9.534761	0.641723	0.468188
68	1	0	-10.606596	0.719995	0.224232
69	1	0	-9.175881	1.636022	0.776032
70	1	0	-9.421343	-0.047079	1.319511
71	6	0	-8.931535	1.114448	-1.945565
72	1	0	-8.381197	0.768101	-2.833935
73	1	0	-8.562121	2.116909	-1.679500
74	1	0	-9.998805	1.195971	-2.207690
75	6	0	-9.279576	-1.270538	-1.169755
76	1	0	-9.161746	-1.988843	-0.343839
77	1	0	-8.737038	-1.657118	-2.046253
78	1	0	-10.349773	-1.205487	-1.424621
79	1	0	2.573475	-0.658391	-1.351485
80	1	0	4.080109	-1.228495	1.149907
81	1	0	4.647392	-0.964996	-0.492758
82	1	0	3.795746	2.382089	1.646128
83	6	0	0.463931	2.923394	-1.026089
84	1	0	-0.323334	2.326541	-0.542716
85	1	0	0.560350	3.865675	-0.464815
86	1	0	0.117233	3.176784	-2.043344
87	6	0	0.678197	-2.217640	-1.872357
88	1	0	-0.024610	-3.062362	-1.983228
89	1	0	0.087189	-1.290937	-1.821097
90	1	0	1.291689	-2.174088	-2.786256
91	1	0	1.537706	-1.418321	2.257038
92	1	0	-0.486047	-1.387879	1.739594
93	1	0	-1.226037	0.915780	3.520848

6b(*S*)

Sum of electronic and zero-point Energies= -1611.438705
Sum of electronic and thermal Energies= -1611.396232
Sum of electronic and thermal Enthalpies= -1611.395288
Sum of electronic and thermal Free Energies= -1611.518253

Standard orientation:

Center Number	Atomic Number	Atomic Type	Coordinates (Angstroms) X	Y	Z
1	5	0	-1.571626	-0.066951	-0.319317
2	6	0	-0.099689	-0.330755	3.011945
3	1	0	0.744239	0.044746	3.615674
4	1	0	-0.024993	-1.429777	2.989077
5	6	0	-1.381089	0.092500	3.655311
6	6	0	-1.821873	1.430035	4.232858
7	6	0	-2.528761	-0.529323	4.001327
8	6	0	-3.150868	0.704006	4.618195
9	1	0	-1.225530	1.787314	5.090477
10	1	0	-1.934979	2.261722	3.517017
11	1	0	-2.882183	-1.559770	3.895403
12	1	0	-3.351653	0.651776	5.702392
13	1	0	-4.054135	1.091317	4.115644
14	6	0	-1.667406	1.461278	-0.805067
15	6	0	-2.349403	2.445633	0.202878
16	6	0	-0.336662	2.025571	-1.419371
17	6	0	-1.866473	3.898492	0.052809
18	6	0	-0.268379	3.564683	-1.291673
19	6	0	-1.642078	4.321850	-1.440138
20	6	0	-0.328942	3.917118	0.215159
21	1	0	0.578604	3.958484	-1.880831
22	1	0	-2.467251	4.576309	0.684333
23	6	0	-2.446481	-1.201434	-1.035783
24	6	0	-3.904969	-1.176443	-0.414037
25	6	0	-1.791988	-2.620102	-1.056199
26	6	0	-4.445844	-2.603366	-0.191269
27	6	0	-2.741933	-3.757870	-0.662946
28	1	0	-0.939513	-2.652016	-0.357949
29	6	0	-3.421835	-3.380130	0.673349
30	6	0	-4.140759	-3.623699	-1.348525
31	1	0	-2.235632	-4.736557	-0.736175
32	1	0	-5.491401	-2.556550	0.162251
33	1	0	0.527109	1.653376	-0.838614
34	1	0	-3.852235	-0.739876	0.601923
35	1	0	-2.573720	-0.883677	-2.084944
36	6	0	-4.203077	-3.212911	-2.822065
37	1	0	-3.817336	-4.032801	-3.453291
38	1	0	-5.244636	-3.026220	-3.134939
39	6	0	-4.983154	-4.899590	-1.186696
40	1	0	-6.030701	-4.709156	-1.477423
41	1	0	-4.989419	-5.284009	-0.155789
42	1	0	-4.597060	-5.701399	-1.839504
43	1	0	-2.823446	-2.786579	1.383595
44	1	0	-3.857518	-4.238564	1.203194
45	1	0	0.109465	3.185721	0.913567
46	1	0	0.076260	4.909435	0.453815
47	6	0	-2.675521	3.952508	-2.510123
48	1	0	-3.590666	4.552400	-2.363276
49	1	0	-2.971993	2.896968	-2.509043
50	1	0	-2.291437	4.191697	-3.516442
51	6	0	-1.409228	5.839188	-1.565966
52	1	0	-2.355511	6.385825	-1.412949

53	1	0	-1.043195	6.085645	-2.577295
54	1	0	-0.677831	6.231533	-0.844555
55	8	0	-0.783973	-0.526932	0.708138
56	6	0	0.103634	0.181883	1.563924
57	1	0	-0.105530	1.262193	1.531319
58	6	0	1.537333	-0.043402	1.124313
59	6	0	2.557294	0.862825	1.451109
60	6	0	1.952452	-1.175154	0.408971
61	7	0	3.833520	0.673311	1.116110
62	1	0	2.330123	1.783243	2.002834
63	7	0	3.224944	-1.386707	0.064817
64	1	0	1.226147	-1.932059	0.097518
65	6	0	4.120087	-0.453288	0.435664
66	6	0	5.494603	-0.674993	0.073725
67	6	0	6.660243	-0.865895	-0.232254
68	6	0	8.065667	-1.101883	-0.603829
69	6	0	8.535515	-2.432422	0.029324
70	1	0	9.586055	-2.621769	-0.244815
71	1	0	7.925471	-3.276615	-0.327384
72	1	0	8.465325	-2.393862	1.127434
73	6	0	8.169879	-1.190681	-2.144360
74	1	0	7.835994	-0.254287	-2.617544
75	1	0	7.553657	-2.015146	-2.534981
76	1	0	9.217710	-1.371755	-2.434079
77	6	0	8.935160	0.064879	-0.083632
78	1	0	8.871216	0.146587	1.012509
79	1	0	8.612760	1.022733	-0.520485
80	1	0	9.988871	-0.104974	-0.358295
81	1	0	-2.354761	1.408309	-1.662038
82	1	0	-2.174456	2.146344	1.250636
83	1	0	-3.616341	-2.314549	-3.053618
84	1	0	-3.441870	2.391376	0.058017
85	1	0	-1.377791	-2.808341	-2.061939
86	6	0	-4.872818	-0.295216	-1.213978
87	1	0	-4.508900	0.742155	-1.281987
88	1	0	-5.014888	-0.665519	-2.240927
89	1	0	-5.862694	-0.265845	-0.727342
90	6	0	-0.136851	1.530025	-2.856918
91	1	0	0.823083	1.886880	-3.266287
92	1	0	-0.125299	0.427698	-2.899333
93	1	0	-0.939613	1.880352	-3.525750

6b(*R*)

Sum of electronic and zero-point Energies= -1611.433597
Sum of electronic and thermal Energies= -1611.391231
Sum of electronic and thermal Enthalpies= -1611.390287
Sum of electronic and thermal Free Energies= -1611.511905

Standard orientation:

Center Number	Atomic Number	Atomic Type	Coordinates (Angstroms) X	Y	Z
1	5	0	-1.664567	-0.047641	0.253794
2	6	0	-0.512323	1.976115	2.505506
3	1	0	-0.378166	2.584333	1.595524
4	1	0	0.206714	2.387079	3.238254
5	6	0	-1.887366	2.179356	3.056366
6	6	0	-2.649375	3.468222	3.313677
7	6	0	-2.874024	1.397034	3.545747
8	6	0	-3.785523	2.557943	3.881281
9	1	0	-2.190889	4.154571	4.046811
10	1	0	-2.921089	4.047709	2.414320
11	1	0	-2.987046	0.316373	3.667284
12	1	0	-4.002844	2.705103	4.953558
13	1	0	-4.737731	2.601563	3.324689
14	6	0	-1.527027	1.293085	-0.617895
15	6	0	-0.072075	1.474350	-1.183870
16	6	0	-2.643954	1.518588	-1.690547
17	6	0	-0.031200	2.152845	-2.560737
18	6	0	-2.105701	2.238192	-2.945532
19	6	0	-1.029934	3.353239	-2.673822
20	6	0	-0.957601	1.371215	-3.520290
21	1	0	-2.938309	2.508264	-3.619140
22	1	0	1.013053	2.332774	-2.870728
23	6	0	-2.528522	-1.311066	-0.218948
24	6	0	-3.053692	-2.242065	0.921315
25	6	0	-1.698606	-2.065642	-1.327391
26	6	0	-2.919994	-3.735356	0.559365
27	6	0	-1.784087	-3.594126	-1.216368
28	1	0	-0.626779	-1.800110	-1.280442
29	6	0	-1.430409	-4.005635	0.231509
30	6	0	-3.241138	-4.092226	-0.938718
31	1	0	-1.235332	-4.073309	-2.046234
32	1	0	-3.401999	-4.363170	1.329996
33	1	0	-2.986315	0.536670	-2.062453
34	1	0	-2.401410	-2.100608	1.800716
35	1	0	-3.420872	-0.916928	-0.732366
36	6	0	-4.405668	-3.441547	-1.690506
37	1	0	-4.367175	-3.726155	-2.756815
38	1	0	-5.373131	-3.796372	-1.295939
39	6	0	-3.366672	-5.613634	-1.124986
40	1	0	-4.326758	-5.972264	-0.715519
41	1	0	-2.563048	-6.177605	-0.628448
42	1	0	-3.344199	-5.874491	-2.197185
43	1	0	-0.688651	-3.383988	0.760129
44	1	0	-1.139740	-5.060862	0.326353
45	1	0	-1.033187	0.282612	-3.363314
46	1	0	-0.748053	1.561145	-4.581769
47	6	0	-1.161161	4.339558	-1.509290
48	1	0	-0.242266	4.946477	-1.428788
49	1	0	-1.326842	3.859723	-0.536489
50	1	0	-1.996476	5.039079	-1.683667
51	6	0	-0.773884	4.185535	-3.942263
52	1	0	0.142284	4.790020	-3.827706

53	1	0	-1.611812	4.881544	-4.119166
54	1	0	-0.656317	3.571360	-4.847119
55	8	0	-1.007392	-0.247872	1.445914
56	6	0	-0.117308	0.514485	2.268667
57	6	0	1.315635	0.324396	1.790721
58	6	0	2.282310	1.337466	1.735401
59	6	0	1.770487	-0.946233	1.398710
60	7	0	3.537409	1.124969	1.329000
61	1	0	2.046540	2.365559	2.027006
62	7	0	3.016715	-1.182777	0.991591
63	1	0	1.090380	-1.804327	1.414272
64	6	0	3.856923	-0.129510	0.968291
65	6	0	5.204136	-0.369226	0.524806
66	6	0	6.346131	-0.574001	0.147038
67	6	0	7.725460	-0.823426	-0.304571
68	6	0	8.535876	-1.417690	0.871621
69	1	0	9.571076	-1.610262	0.546133
70	1	0	8.096909	-2.367547	1.213909
71	1	0	8.563872	-0.721328	1.723957
72	6	0	7.699818	-1.825461	-1.481580
73	1	0	7.123394	-1.424051	-2.329431
74	1	0	7.246243	-2.781169	-1.176663
75	1	0	8.728863	-2.022098	-1.823645
76	6	0	8.361572	0.509563	-0.759634
77	1	0	8.385410	1.237607	0.065935
78	1	0	7.796165	0.952153	-1.594319
79	1	0	9.395758	0.332069	-1.096622
80	1	0	-1.666250	2.108630	0.110696
81	1	0	0.445375	0.503912	-1.283895
82	1	0	-4.405581	-2.345247	-1.640742
83	1	0	0.524649	2.057461	-0.463238
84	1	0	-2.044821	-1.738570	-2.322142
85	6	0	-4.472368	-1.853294	1.356834
86	1	0	-4.515616	-0.794114	1.661528
87	1	0	-5.208527	-1.994762	0.549339
88	1	0	-4.800928	-2.459095	2.218754
89	6	0	-3.871502	2.203052	-1.077603
90	1	0	-4.688412	2.287122	-1.814137
91	1	0	-4.256286	1.628265	-0.217960
92	1	0	-3.638625	3.218215	-0.717201
93	1	0	-0.185506	0.012270	3.249883

9

Sum of electronic and zero-point Energies= -10812.286310
Sum of electronic and thermal Energies= -10812.176415
Sum of electronic and thermal Enthalpies= -10812.175471
Sum of electronic and thermal Free Energies= -10812.458301

Standard orientation:

Center Number	Atomic Number	Atomic Type	Coordinates (Angstroms) X	Y	Z
1	6	0	-4.094695	-4.443206	-0.218844
2	7	0	-3.041084	-3.794747	0.320067
3	6	0	-3.313930	-2.656191	0.962429
4	6	0	-4.602918	-2.126733	1.087350
5	6	0	-5.606762	-2.887530	0.481434
6	7	0	-5.376305	-4.034207	-0.164922
7	6	0	-4.881689	-0.812442	1.797837
8	6	0	-3.827342	-5.670294	-0.911753
9	6	0	-3.614544	-6.712562	-1.494813
10	6	0	-3.370441	-7.984835	-2.195121
11	6	0	-3.328875	-9.130368	-1.154513
12	8	0	-3.885909	0.139577	1.468235
13	30	0	-4.231051	1.593002	0.019380
14	6	0	-6.000748	2.508655	0.071267
15	6	0	-7.170506	1.664110	-0.457079
16	30	0	-2.820868	1.250339	2.636390
17	6	0	-2.234694	1.832258	4.423074
18	6	0	-1.046882	1.045208	4.997049
19	8	0	-2.522441	2.253316	0.950450
20	6	0	-1.917635	3.519131	0.983234
21	6	0	-0.417416	3.418834	0.727825
22	6	0	0.465475	4.464444	1.029179
23	7	0	1.760912	4.442176	0.717636
24	6	0	2.232409	3.338652	0.110651
25	7	0	1.497363	2.224357	-0.115761
26	6	0	0.187754	2.283544	0.190950
27	30	0	2.273950	0.361423	-0.989035
28	6	0	2.888023	0.577670	-2.885635
29	6	0	4.385933	0.668025	-3.203896
30	6	0	3.599151	3.406449	-0.307915
31	6	0	4.743393	3.682517	-0.609307
32	6	0	6.115781	4.094425	-0.952299
33	6	0	6.446902	5.378141	-0.151271
34	8	0	0.600231	-0.887474	-0.723667
35	30	0	1.286107	-1.919732	0.785819
36	8	0	2.810003	-0.695034	0.667249
37	6	0	3.734643	-0.795880	1.732995
38	6	0	5.167485	-0.899821	1.232434
39	6	0	6.224482	-1.362274	2.027659
40	7	0	7.481113	-1.470435	1.589113
41	6	0	7.701457	-1.113013	0.308148
42	7	0	6.764916	-0.681363	-0.556890
43	6	0	5.518872	-0.587440	-0.080713
44	6	0	9.049448	-1.209731	-0.175009
45	6	0	10.189798	-1.289565	-0.579991
46	6	0	11.573918	-1.385284	-1.073702
47	6	0	-0.258841	-1.609459	-1.584477
48	6	0	-1.330787	-0.682944	-2.143134
49	6	0	-1.519054	-0.324804	-3.479509
50	7	0	-2.497753	0.491065	-3.889697
51	6	0	-3.324666	0.984570	-2.957333
52	7	0	-3.220616	0.714564	-1.630342

53	6	0	-2.238065	-0.111584	-1.253699
54	6	0	-4.374052	1.841056	-3.412229
55	6	0	-5.231144	2.551277	-3.896551
56	6	0	-6.260486	3.405759	-4.510888
57	6	0	-5.800630	3.794084	-5.936744
58	6	0	0.652674	-3.253716	2.085335
59	6	0	1.646871	-4.402537	2.314074
60	6	0	0.256354	-2.608390	3.423232
61	6	0	2.150589	1.771188	-3.518690
62	6	0	-3.379350	1.911396	5.444345
63	6	0	-6.317797	3.023970	1.484881
64	6	0	-7.591536	2.618461	-4.587097
65	6	0	-6.453605	4.680576	-3.656035
66	6	0	6.195145	4.396419	-2.468809
67	6	0	7.120298	2.978459	-0.584097
68	6	0	-4.514062	-8.238433	-3.205521
69	6	0	-2.017944	-7.910609	-2.942033
70	1	0	-0.871010	-0.708388	-4.263424
71	1	0	-2.189215	-0.322722	-0.191562
72	1	0	-0.257132	-3.700812	1.656657
73	1	0	-0.138569	-3.354691	4.133343
74	1	0	1.110484	-2.126286	3.918970
75	1	0	-0.517693	-1.838603	3.302977
76	1	0	1.895371	-4.925651	1.382300
77	1	0	1.244066	-5.154902	3.013249
78	1	0	2.593657	-4.049359	2.746729
79	1	0	2.519363	-0.325914	-3.395287
80	1	0	2.360278	1.858724	-4.598919
81	1	0	2.454676	2.722999	-3.062681
82	1	0	1.059473	1.698543	-3.410183
83	1	0	4.931435	-0.246192	-2.940791
84	1	0	4.559462	0.842008	-4.280343
85	1	0	4.869210	1.495391	-2.669121
86	1	0	0.110975	5.361449	1.530824
87	1	0	-0.383265	1.382727	0.003350
88	1	0	-3.036451	2.335249	6.403104
89	1	0	-3.796279	0.921373	5.672977
90	1	0	-4.209221	2.538325	5.093354
91	1	0	-0.715037	1.462011	5.962846
92	1	0	-1.299118	-0.007752	5.179997
93	1	0	-0.177253	1.054316	4.327007
94	1	0	-1.881969	2.864827	4.262427
95	1	0	-5.922051	3.391555	-0.582519
96	1	0	-6.385404	2.201375	2.211678
97	1	0	-7.282002	3.559826	1.528780
98	1	0	-5.549670	3.715897	1.857328
99	1	0	-8.125164	2.219155	-0.437248
100	1	0	-7.329892	0.759693	0.147477
101	1	0	-7.006810	1.332442	-1.489950
102	1	0	-8.358142	3.244304	-5.057929
103	1	0	-7.476448	1.707577	-5.183415
104	1	0	-7.940813	2.336745	-3.588987
105	1	0	-6.770636	4.429288	-2.639055
106	1	0	-5.525033	5.257921	-3.595479
107	1	0	-7.222416	5.314448	-4.112342
108	1	0	-4.857137	4.348815	-5.908679
109	1	0	-5.658364	2.905457	-6.560086
110	1	0	-6.560095	4.428627	-6.407506
111	1	0	5.491877	5.187056	-2.750164
112	1	0	7.208397	4.730625	-2.719221
113	1	0	5.969642	3.506049	-3.062993
114	1	0	7.086075	2.757979	0.488116
115	1	0	6.917789	2.052714	-1.129870
116	1	0	8.135477	3.309369	-0.831579

117	1	0	5.749282	6.185989	-0.394863
118	1	0	6.396060	5.193094	0.926591
119	1	0	7.461798	5.711286	-0.396617
120	1	0	6.051770	-1.659442	3.059821
121	1	0	4.757138	-0.257121	-0.777646
122	1	0	-2.461761	-2.135057	1.392166
123	1	0	-6.643765	-2.556312	0.512726
124	1	0	-5.482023	-8.293711	-2.696946
125	1	0	-4.564010	-7.438061	-3.951098
126	1	0	-4.343467	-9.186669	-3.728400
127	1	0	-2.021961	-7.104062	-3.682697
128	1	0	-1.193179	-7.732115	-2.244396
129	1	0	-1.830415	-8.856287	-3.463718
130	1	0	-2.526885	-8.970977	-0.426437
131	1	0	-4.276827	-9.202401	-0.611531
132	1	0	-3.148968	-10.084348	-1.663650
133	6	0	11.598037	-2.258301	-2.351153
134	6	0	12.092631	0.034999	-1.404182
135	6	0	12.464743	-2.026637	0.015802
136	1	0	13.122510	-0.024135	-1.775316
137	1	0	11.474954	0.509315	-2.173813
138	1	0	12.083151	0.673351	-0.514569
139	1	0	13.497676	-2.100636	-0.344003
140	1	0	12.460349	-1.424931	0.930610
141	1	0	12.114157	-3.033099	0.266846
142	1	0	12.624400	-2.333819	-2.729009
143	1	0	11.232401	-3.269117	-2.142679
144	1	0	10.971880	-1.822388	-3.136437
145	6	0	0.479809	-2.402527	-2.679242
146	6	0	-0.398938	-3.522390	-3.334636
147	6	0	1.378306	-3.512750	-2.144147
148	6	0	0.358819	-4.588754	-2.486780
149	1	0	-0.213534	-3.622562	-4.407549
150	1	0	-1.477880	-3.452108	-3.166064
151	1	0	0.737038	-5.472554	-3.011111
152	1	0	-0.228205	-4.924879	-1.623481
153	6	0	-5.042879	-0.956795	3.326240
154	6	0	-4.096095	-1.853981	4.189546
155	6	0	-6.219721	-1.755406	3.882440
156	6	0	-5.344231	-2.407981	4.941726
157	1	0	-3.351134	-1.330780	4.793835
158	1	0	-3.594339	-2.629971	3.606402
159	1	0	-5.475094	-1.961926	5.935422
160	1	0	-5.419646	-3.496102	5.043089
161	6	0	-2.622655	4.485121	-0.008609
162	6	0	-2.274819	4.380564	-1.529616
163	6	0	-2.178615	5.946882	-0.049026
164	6	0	-1.740872	5.846027	-1.503836
165	1	0	-3.148468	4.234333	-2.168731
166	1	0	-1.529168	3.623369	-1.787646
167	1	0	-2.229640	6.545144	-2.191717
168	1	0	-0.657339	5.929920	-1.652952
169	6	0	3.516170	0.311208	2.798297
170	6	0	4.265871	1.681980	2.751069
171	6	0	4.079554	0.106619	4.206449
172	6	0	4.610291	1.531287	4.263592
173	1	0	3.662497	2.550811	2.481902
174	1	0	5.151430	1.661667	2.110767
175	1	0	4.016831	2.187742	4.911774
176	1	0	5.664503	1.645102	4.540680
177	6	0	2.648438	-3.542242	-1.739590
178	1	0	3.247266	-2.637998	-1.670193
179	1	0	3.141267	-4.479361	-1.488677
180	1	0	0.974266	-1.711250	-3.369129

181	6	0	-7.490514	-1.903457	3.517058
182	1	0	-7.920121	-1.351457	2.684059
183	1	0	-8.156005	-2.583749	4.043951
184	1	0	-5.055905	0.069562	3.720606
185	6	0	4.132498	-0.931186	5.038845
186	1	0	3.700864	-1.897114	4.784603
187	1	0	4.612164	-0.854453	6.012400
188	1	0	2.428895	0.460571	2.843679
189	6	0	-2.238238	6.942890	0.832727
190	1	0	-2.634501	6.796618	1.835624
191	1	0	-1.899145	7.946315	0.583934
192	1	0	-3.693642	4.377729	0.201393
193	1	0	-2.040745	3.973652	1.982263
194	1	0	-5.853950	-0.448060	1.430249
195	1	0	-0.806494	-2.353051	-0.979714
196	1	0	3.529114	-1.739837	2.264169
